# Genome-wide identification of significant aberrations in cancer genome

**DOI:** 10.1186/1471-2164-13-342

**Published:** 2012-07-27

**Authors:** Xiguo Yuan, Guoqiang Yu, Xuchu Hou, Ie-Ming Shih, Robert Clarke, Junying Zhang, Eric P Hoffman, Roger R Wang, Zhen Zhang, Yue Wang

**Affiliations:** 1 School of Computer Science and Technology, Xidian University, Xi'an, P. R. China; 2 Bradley Department of Electrical and Computer Engineering, Virginia Polytechnic Institute and State University, Arlington, VA, USA; 3 Center for Sleep Sciences and Medicine, Stanford University School of Medicine, Palo Alto, CA, 94304, USA; 4 Departments of Gynecology/Obstetrics and Oncology, Johns Hopkins University School of Medicine, Baltimore, MD, 21231, USA; 5 Lombardi Comprehensive Cancer Center and Department of Oncology, Georgetown University, Washington, DC, 20057, USA; 6 Research Center for Genetic Medicine, Children's National Medical Center, Washington, DC, 20010, USA; 7 The International Baccalaureate Magnet Diploma Program, Richard Montgomery High School, Rockville, MD, 20852, USA; 8 Department of Pathology, Johns Hopkins Medical Institutions, Baltimore, MD, 21231, USA

## Abstract

**Background:**

Somatic Copy Number Alterations (CNAs) in human genomes are present in almost all human cancers. Systematic efforts to characterize such structural variants must effectively distinguish significant consensus events from random background aberrations. Here we introduce Significant Aberration in Cancer (SAIC), a new method for characterizing and assessing the statistical significance of recurrent CNA units. Three main features of SAIC include: (1) exploiting the intrinsic correlation among consecutive probes to assign a score to each CNA unit instead of single probes; (2) performing permutations on CNA units that preserve correlations inherent in the copy number data; and (3) iteratively detecting Significant Copy Number Aberrations (SCAs) and estimating an unbiased null distribution by applying an SCA-exclusive permutation scheme.

**Results:**

We test and compare the performance of SAIC against four peer methods (GISTIC, STAC, KC-SMART, CMDS) on a large number of simulation datasets. Experimental results show that SAIC outperforms peer methods in terms of larger area under the *Receiver Operating Characteristics* curve and increased detection power. We then apply SAIC to analyze structural genomic aberrations acquired in four real cancer genome-wide copy number data sets (ovarian cancer, metastatic prostate cancer, lung adenocarcinoma, glioblastoma). When compared with previously reported results, SAIC successfully identifies most SCAs known to be of biological significance and associated with oncogenes (e.g., KRAS, CCNE1, and MYC) or tumor suppressor genes (e.g., CDKN2A/B). Furthermore, SAIC identifies a number of novel SCAs in these copy number data that encompass tumor related genes and may warrant further studies.

**Conclusions:**

Supported by a well-grounded theoretical framework, SAIC has been developed and used to identify SCAs in various cancer copy number data sets, providing useful information to study the landscape of cancer genomes. Open–source and platform-independent SAIC software is implemented using C++, together with R scripts for data formatting and Perl scripts for user interfacing, and it is easy to install and efficient to use. The source code and documentation are freely available at http://www.cbil.ece.vt.edu/software.htm.

## Background

Somatic copy number alterations (CNAs) are common genetic events in the development and progression of various human cancers, and significantly contribute to tumorigenesis [1,2]. The coverage of CNAs in tumors varies from a few hundred to several million nucleotide bases, consisting of both deletions and amplifications with highly complex patterns [3,4]. Recent advances in oligonucleotide-based single nucleotide polymorphism (SNP) arrays have made it possible to detect regional amplifications and deletions with high resolution on a genome-wide scale [5,6]. A critical challenge in the genome-wide analysis of CNAs is to distinguish between the “driver” mutations that allow the tumor to initiate, grow, and persist, and the “passenger” mutations that represent random somatic events accumulated during tumorigenesis [1,3,7]. Identification of these “driver” alterations can provide important insights into the cellular defects that cause cancer and suggest potential diagnostic, prognostic, and targeted therapeutic strategies [1,7,8].

By studying a sufficiently large collection of cancer samples, Significant Copy Number Aberrations (SCAs), defined as significantly recurrent CNAs that affect the same region in multiple tumors, are widely considered as informative surrogates of “driver” mutations that may help pinpoint novel cancer-causing genes [3,9]. Past studies have detected many SCAs in a wide range of cancer types, with an impressive coverage of many known oncogenes and cancer suppressor genes [1,2,7]. Several methods for finding regions of SCAs using CNAs data have been described in the literature, where the task of distinguishing between sporadic CNAs and SCAs is largely a statistical significance testing. Two reviews with qualitative comparison of different methods have been published [10,11]. Despite the use of different algorithms, a common theme in these methods is that they often adopt a four-step strategy: (1) detect CNAs and separate deletions and amplifications; (2) design and calculate ensemble test statistics associated with a genomic locus; (3) construct and/or estimate the probability distribution of test statistics under the null hypothesis; (4) perform multiple testing on a pool of genomic loci.

Significance testing for aberrant copy number (STAC) starts by converting the normalized log-ratios into a binary matrix, with zeros indicating no change and ones indicting losses and gains [12]. STAC then proposes two statistics (footprint and frequency) to define regions of SCAs while adjusting for multiple comparisons, where the null hypothesis is that the detected CNAs from single-sample analysis are the realizations of random CNA placements whose probability distribution is generated by permutations on CNA segments [13]. Genomic Identification of Significant Targets in Cancer (GISTIC) works on the real-valued step function of log-ratios that allows GISTIC to exploit both the type (amplification/deletion) and amplitude of CNAs [1,3]. Using a semi-parametric permutation assuming independence between probes, GISTIC calculates a score that is based on both the amplitude and frequency of CNAs at each probe position and subsequently identify regions of SCAs, where amplification and deletion CNAs are handled separately, and armed-level and focal CNAs are further analyzed independently [14]. Aimed to correlate information from neighboring probes with the amplitude and frequency of CNAs at each probe position, Kernel Convolution – a Statistical Method for Aberrant Regions detection (KC-SMART) uses varying-width kernel functions to calculate the testing statistics from the original log-ratios across multiple samples, producing the kernel smoothed estimate (KSE) at each locus by locally weighted regression [15]. SCAs are selected based on a permutation-generated null distribution and Bonferroni correction. To substantially reduce computational burden in analyzing high-resolution and large-population data, correlation matrix diagonal segmentation (CMDS) identifies SCAs based on a between-chromosomal-site correlation analysis directly using the raw intensity ratios across all samples [16]. CMDS uses a correlation statistics to detect SCAs with a standard normal null distribution whose parameters are estimated directly from the data and adjusts for multiple comparisons by false discovery rate.

Existing methods have several limitations. When working with unprocessed raw intensity ratios [13,15,16], most methods are oblivious to noise clutter that can significantly confound estimation of the null distribution about true yet sporadic CNAs [9,17]. Furthermore, these methods cannot distinguish between contributions of amplifications and deletions to the calculated overall test statistics that may affect the power to detect SCAs. While some effort has been made to incorporate correlation among neighboring probes into the test statistics, most methods assign a score to, and test the significance at, each individual probe locus [14,15]. In addition, while it is widely accepted that CNAs signals at adjacent probes are highly correlated [9,13-15], the assumption of probe independence is often adopted in constructing and learning the null distribution, probably for mathematical convenience [3,16]. Moreover, existing permutation experiments using multiple samples cannot distinguish between the contributions of sporadic CNAs (obeying null distribution) and actual SCAs (deviating from null distribution) to the estimation of null distributions, resulting in theoretically conservative estimations especially when the number of true SCAs participating in the permutation is large.

We now report Significant Aberration in Cancer (SAIC), a carefully motivated method for accurately identifying SCAs using CNAs data from multiple samples. To distinguish between different biological roles of CNAs types and between noise and sporadic CNAs, we use discretized CNAs data and separately analyze copy number amplifications and deletions. By exploiting the intrinsic correlation among consecutive probes, we calculate and assign a score (test statistics) to each CNA unit instead of each single probe, based on both the amplitude and frequency of CNAs within the unit. To accurately estimate the null distribution governing sporadic CNAs, we perform random positional permutations on CNA units that preserve correlations inherent to the copy number data. More importantly, to minimize the unwanted participation of true SCAs in determining the null distribution [3,14], we iteratively detect SCAs and estimate an unbiased null distribution by an SCA-exclusive permutation scheme.

We tested SAIC on extensive simulation data sets, observing significantly improved performance with larger areas under the *Receiver Operating Characteristics* (ROC) curves and higher sensitivities at acceptable low false discovery rates, as compared to four popular peer methods (GISTIC, STAC, KC-SMART, and CMDS). We then applied SAIC to four real benchmark data sets, successfully identified the majority (84%) of previously reported SCAs harboring regions associated with well-known tumor-causing genes, and more importantly, detected some novel SCAs partially validated by the presence of known cancer-related genes.

## Methods

### Data format and definitions

Preprocessed log-ratio data are stored in a numeric *N* ×*M* matrix *X*. Each entry *x*_*nm*_ represents DNA copy number (in log2-ratio) for sample *n* at probe *m*, where each row *X*_*n*_ corresponds to copy number for *n*th sample at *M* probes. Copy number amplifications and deletions are analyzed separately. We use the indicator function to divide matrix *X* into two matrices *X* = *X*_amplification_ +*X*_deletion_, where

(1) $$\begin{array}{l} X_{\text{amplification}}=\left\{I\left(x_{\mathrm{nm}}\geq \theta_{\text{amplification}}\right)\cdot x_{\mathrm{nm}}\right\},\\ X_{\text{deletion}}=\left\{I\left(x_{\mathrm{nm}}\leq \theta_{\text{deletion}}\right)\cdot x_{\mathrm{nm}}\right\}\text{,} \end{array}$$

with *θ*_amplification_ and *θ*_deletion_ being the pre-specified thresholds. For brevity, we focus all subsequent discussion on *X*_amplification_ and make comments on *X*_deletion_ when necessary.

#### Definition 1

Any copy number probe *m* whose associated copy number is amplified or deleted in at least one of *N* samples is called a CNA probe.

**Figure 1:**
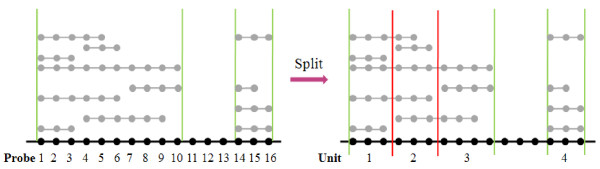
**An illustration on how CNA units are defined.** Left: Consecutive CNA probes are merged into two intervals, with the first interval containing probes 1–10 and the second interval containing probes 14–16. Right: Each of the two intervals is split into CNA units according to the correction coefficients between CNA probes defined by Eq. (2), *e.g.,* the first interval is split into three independent CNA units.

To exploit correlations inherent in copy number data, we first merge consecutive CNA probes into CNA regions, leaving the gaps consisting of only non CNA probes, see Figure 1. Within each CNA region, the Pearson correlation coefficient *ρ*_ij_ between CNA probes *i* and *j* is then calculated for $\left\{i\neq j\right\}\in M$:

(2) $$\rho_{\mathrm{ij}}=\frac{\sum_{n=1}^{N}(x_{\mathrm{ni}}-{\bar{x}}_{i})(x_{\mathrm{nj}}-{\bar{x}}_{j})}{(N-1)s_{i}s_{j}}\text{,}$$

where ${\bar{x}}_{i}$, ${\bar{x}}_{j}$, *s*_*i*_ and *s*_*j*_ are the estimated means and standard deviations of copy numbers at probes *i* and *j* across *N* samples, respectively. If *ρ*_ij_ is less than a pre-specified threshold *θρ*, a breakpoint occurs between probes *i* and *j*.

#### Definition 2

A sequence of consecutive CNA probes with no breakpoints is defined as a CNA unit, denoted by *u* (*k, L*) with *k* being the starting probe index and *L* being the length of the CNA unit.

Intuitively, a CNA unit consists of a sequence of highly correlated consecutive CNA probes. Figure 1 illustrates the concepts of CNA region and CNA unit, where two CNA regions contain 10 and 3 CNA probes, respectively, and the first CNA region is further split into three CNA units due to two breakpoints within the CNA region.

### Summary statistics and significance assessment

Units that exhibit high or low average copy number are of interest, so it is natural to examine summary statistics for each unit. SAIC identifies significant aberration units through two steps. First, the method calculates a statistic (*U* score) that incorporates both the frequencies of occurrence and the amplitudes of the CNA probes within the unit, leading to the unit summary statistics given by

(3) $$U_{{}^{k\text{,}\;L}}^{}=\frac{1}{LN}\sum_{n=1}^{N}\;\sum_{l=k}^{k+L-1}x_{\mathrm{nl}}^{}\text{.}$$

Second, the method assesses the statistical significance of each CNA unit by comparing the observed statistic to the *U* scores that would be expected by chance.

Sporadic CNA units often occur throughout the genome, so a null distribution for *U*_*k, L*_ under the hypothesis that no SCAs are present, can be estimated by randomly permuting the overall pattern of presumed all-sporadic CNA units across the genome [3,9,12,15]. Though various permutation schemes can be adopted, due to different rates of CNA and different percentages of normal tissue contamination in tumor samples [18], permutation of CNA units across rows/samples should be avoided. As aforementioned, permutation should be performed on CNA units (instead of single CNA probes) that preserve correlations inherent to the copy number data, even if the CNA units are sporadic [3,9,15]. Another subtle but conveniently ignored issue is the different background rates of CNA units with varying lengths [1]. Short CNA units occur at a frequency inversely related to their lengths and long CNA units occur approximately 30 times more frequently than would be expected by the inverse-length distribution. This observation is seen across all cancer types, is applicable to both copy gains and losses, and is supported by the calculated genome-average background rates for CNAs as a function of length [1]. These considerations motivate our carefully designed SAIC permutation scheme.

Let L denote the integer set containing the lengths of all the observed CNA units in *X*, K denote the integer set containing the starting probe indices of all the observed CNA units in *X*, and *X*^(*t*)^ be the random positional permutation of *X* for *t* = 1,2,…,*T*, with *T* being the total number of permutations. We now describe our method for analyzing CNA units for evidence of significant alteration in cancer, where we account for the difference in background rates between CNA units of different lengths by considering them adaptively.

#### Algorithm 1

Assessing the statistical significance of *U*_*k, L*_

(1) Perform *T* random within-row positional permutations *X*^(*1*)^, *X*^(*2*)^, …, *X*^(*T*)^ of the data matrix *X* on CNA units;

(2) Compute the value of summary statistic $U_{k\text{,}\;L}\left(X^{\left(t\right)}\right)$ for each permuted data set *t* = 1,2,…,*T*, and for each starting probe k=1,2,…,M−L+1 and each length L∈L;

(3) Calculate and assign a P-value to each observed CNA unit *u* (*k, L*) for k∈K based on the extreme right-hand tail probability given by [9,19]

(4) $$P\left(U_{k\text{,}\;L}\left(X\right)\right)=\frac{1+\sum_{t=1}^{T}I\left(\max_{k'}U_{k'\text{,}\;L}\left(X^{\left(t\right)}\right)\geq U_{k\text{,}\;L}\left(X\right)\right)}{T+1}\text{,}$$

where $I\left(\cdot \right)$is the indicator function.

The empirical P-values on *X*_deletion_ are calculated by the extreme left-hand tail probabilities and reversing the inequality in Eq. (4). Both definitions produce P-values that are easy to interpret, and the “max” operation automatically adjusted P-values for multiple comparisons across CNA units thus controls the family-wise error rate [9].

In algorithm 1, it is important to note that when we generate a randomly permuted dataset based on the observed data, we do not re-define the CNA units but re-use the already-defined CNA units. Specifically, in each permutation, we randomly place the already-defined CNA units over the whole genome or each chromosome within each sample, and calculate the summary *U* score for each length of CNA units. Thus, independent of the unit length, the observed CNA units will always be retained (implicitly) in the permuted dataset. Moreover, when the number of permutations is sufficiently large, the p-values of observed CNA units can be accurately estimated. More precisely, to assess the p-value associated with an observed CNA unit of length *L*, we calculate the *U* scores for any consecutive *L* probes (probes do not need to reside within the same unit) across the genome, and compare the maximum score with the score of the observed CNA unit.

### Iterative estimation of unbiased null distribution

One important issue concerning Algorithm 1 is the presence of true SCAs (departing from null distribution) in cancer genomes that presumably contribute high copy number deviations to the estimation of overall null distribution (governing only sporadic CNAs), potentially reducing power to detect less-extreme SCAs due to theoretical conservativeness [9,14]. Loss of power is particularly critical in real-world applications where the number of true SCAs in cancer genomes may be large. Thus, to minimize the unwanted participation of true SCAs in determining the null distribution, we iteratively detect SCAs and estimate an unbiased null distribution by applying an SCA-exclusive permutation scheme. SAIC assesses the ‘new’ SCAs conditional on having found the ‘existing’ SCAs, successively correcting for true SCAs in order to better dissect and detect SCAs. Specifically, the CNA units associated with the ‘existing’ SCAs are masked as zeros after each iteration, resulting in a new data set *X*_-SCAs_ in which already-detected SCAs becomes null.

#### Algorithm 2

Assessing iteratively the statistical significance of *U*_*k, L*_

(1) Perform Algorithm 1;

(2) Check whether ‘new’ SCAs are detected. If ‘yes’, continue; if “no”, stop and re-calculate the P-values for all SCAs using truth converging null distribution;

(3) Mask the CNA units associated with newly detected SCAs as zeros and let $X=X_{-\text{SCAs}}$, then go to step (1).

It has been shown experimentally that additional power to detect SCAs can be gained by removing the effect of newly detected SCAs after each iteration [9]. However, an iterative SCA-exclusive permutation scheme raises another subtle yet critical issue concerning the convergence of null distribution learning and potential bias due to the expected false positive SCAs under the truth-converging null distribution. Fortunately, based on the careful design of Algorithm 2, the following theorem shows that, if we apply a significance level $\alpha '=\alpha /\left(1+\alpha \right)$ where *α* is the targeted false positive rate (FPR), an unbiased estimation and detection results can be readily obtained using Algorithm 2 (see formal proof in Appendix A).

#### Theorem 1

*Suppose that Algorithm 2 is used to iteratively detect SCAs and estimate truth converging null distribution*. *Let αbe the targeted FPR and*$\alpha '=\alpha /\left(1+\alpha \right)$*be the significance level used to detect SCAs. Then an unbiased truth converging null distribution can be obtained together with a theoretical FPR α.*

### SAIC algorithm and data preprocessing

**Figure 2:**
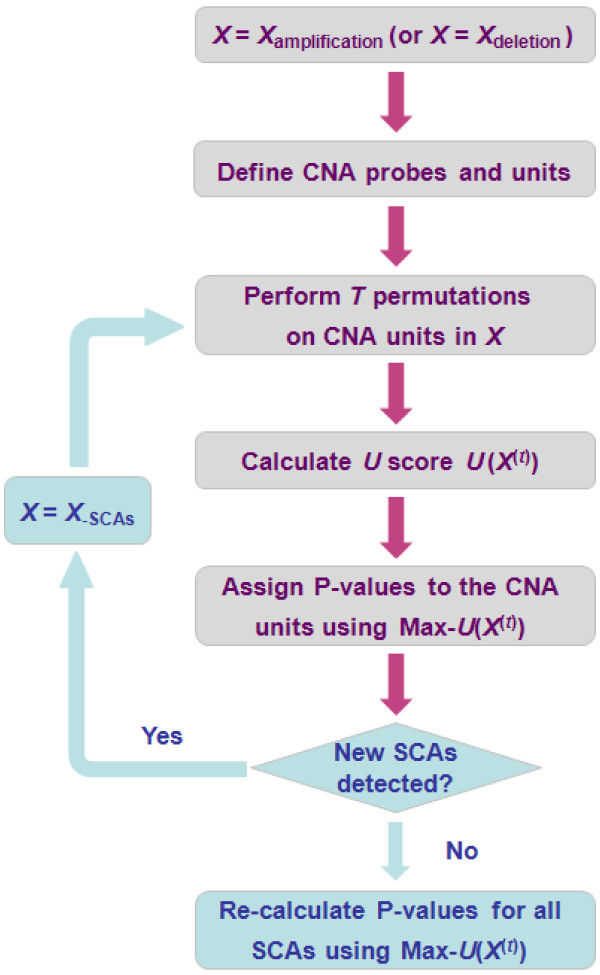
Schematic flowchart of combined SAIC algorithms 1 and 2.

Figure 2 shows the flowchart describing the entire SAIC algorithm. Our algorithm begins with two data preprocessing steps [18]. First, the extracted raw copy number signals from CEL files are normalized using benchmark methods such as dChip (DNA-Chip Analyzer) [20,21]. Second, the normalized copy number signals are segmented into CNA regions using existing single-sample analysis methods such as CBS (Circular Binary Segmentation) [22,23]. The preprocessed log2-transformed ratios are subsequently analyzed by the novel algorithm described here.

## Results

In the absence of definitive ground truth about the recurrent CNAs in the cancer genomes, the validation of a new method for detecting SCAs is always problematic [9,13,16,18,24]. We first validate SAIC on multiple realistic simulation data sets and then proceed to evaluate the method using real CNA data sets. All data sets were analyzed according to the algorithm described in Figure 2. We tested SAIC and the four peer methods (GISTIC, STAC, KC-SMART, CMDS) on realistic simulation data sets. Comparative performance was based on the ground truth in terms of detection power [18] and the *Receiver Operating Characteristics* (ROC) curves [16]. When applied to real CNA data, we compared and discussed biological plausibility of the implicated SCAs, and examined relative SCAs coverage between SAIC and GISTIC on benchmark data sets using Venn diagrams. To assure a meaningful and differential comparison, we emphasized experiment suitability when choosing algorithm parameter settings. For example, the algorithm parameter settings cannot be too “simple” (if there are only a few arm-level SCAs, all methods may perform equally well) or too “complex” (if there are many weak focal SCAs, no method will perform consistently well) [14].

### Simulation studies

Multiple simulation data sets with definitive ground truth and various design or parameter settings were generated based on the modified benchmark models proposed in [9,16,18,24] and as used to assess various performance characteristics [9,16,18]. We first assessed the family-wise type 1 error rate (FWER) whose accuracy is crucial for methods that detect SCAs based on their P-values. If the FWER is either too conservative or too liberal, the P-value loses its intended meaning and does not reflect the actual false positive rate. Thus, we cannot control how many false positives are detected by setting a P-value based threshold [25]. A large number of simulated null data sets (under the null hypothesis that no recurrent CNAs are present) were generated based on the realistic model proposed in [9] and subsequently analyzed with SAIC; results are presented in Table 1. Algorithm 2 was repeated 10,000 times, and the observed FWER was estimated by the proportion of at least one *U*_*k, L*_ (*X*) in *X* that was significant at *α* = 0.05 level [9]. Values of the observed FWER in Table 1 (0.0497) suggest that SAIC is almost perfect when compared with slightly conservative values (0.0452) by similar method [9].

**Table 1:** Empirical type 1 error rate for simulated data sets under the null hypothesis

**Null simulation model**	**Empirical FWER at*α*= 0.05level**
Copy number data	0.0488
Clumped copy number data (25%)	0.0500
Clumped copy number data (50%)	0.0493
Clumped copy number data (75%)	0.0505

We then assessed the detection power of SAIC as compared to GISTIC. Based on the simulation model proposed in [18], we generated 100 simulation data sets under each combinatorial parameter setting, resulting in a total of 1,900 simulation data sets, where each data set consists of *N* = 40 ~ 80 samples and each sample contains *M* = 5,000 probes. To replicate the effect of inevitable normal cell contamination [18], the copy numbers at every probes are simulated by a mixture of normal and tumor genomes, where the normal cell fraction *λ* is randomly drawn from a normal distribution $N\left(\mu_{\lambda },\sigma_{\lambda }\right)$ with *μ*_*λ*_ and *σ*_*λ*_ being the mean and standard deviation of normal cell fraction in the sample. Each sample contains two sporadic CNA regions, one deletion and one amplification randomly drawn from integer sets {0, 1} and {3, 4,…,8}, respectively. Each data set contains two recurrent CNA regions that are contributed from a fraction of samples according to a specified frequency *ω*, one deletion and one amplification similarly designed as aforementioned. The length of both sporadic and recurrent CNA regions is randomly assigned from 150 to 250 probes, realistically reflecting the estimated background rate of focal CNAs in a typical cancer sample genome [1]. To equally assess the power in detecting deletion or amplification SCAs, we calculate the detection power of SAIC or GISTIC as the rate of successfully detecting inserted, deleted or amplified SCAs across 100 data sets. Table 2 summarizes the comparative detection power of SAIC and GISTIC for a total of 19 parameter settings across 1,900 data sets. These comparative experimental results consistently show that SAIC outperforms GISTIC with significantly increased detection power in 18 out of 19 simulations.

**Table 2:** Power to detect SCAs by SAIC and GISTIC in simulation studies

***N* = 60,*ω*** **= 0.2,*μ***_***λ***_ **= 0.6,*σ***_***λ***_**=**	**0.15**	**0.2**	**0.25**	**0.3**	**0.35**
GISTIC	89%	86%	79%	74%	72%
SAIC	96%	94%	86%	86%	82%
*N* = 60, *ω* = 0.2, *σ*_*λ*_ = 0.25, *μ*_*λ*_ =	0.4	0.5	0.6	0.7	0.8
GISTIC	83%	81%	82%	72%	79%
SAIC	93%	91%	87%	79%	74%
*ω* = 0.2, *σ*_*λ*_ = 0.25, *μ*_*λ*_ = 0.6, *N*=	40	50	60	70	80
GISTIC	58%	73%	79%	86%	89%
SAIC	65%	83%	87%	93%	94%
*N* = 60, *σ*_*λ*_ = 0.25, *μ*_*λ*_ = 0.6, *ω* =		0.1	0.15	0.2	0.25
GISTIC		30%	58%	80%	92%
SAIC		37%	72%	87%	97%

**Figure 3:**
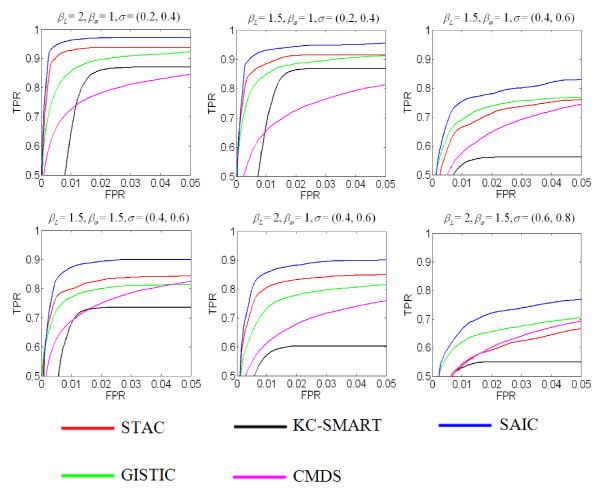
**Comparative performance of SAIC and four peer methods (STAC, GISTIC, KC-SMART, CMDS) on realistic simulation data sets, quantified by the partial ROC curves (north-west) (TPR: true positive rate; FPR: false positive rate).** The results are the averages calculated based on 100 replications under each of various parameter settings.

We further assessed the overall performance of SAIC, measured by both sensitivity and specificity via ROC curves, as compared with the four peer methods (GISTIC, STAC, KC-SMART, CMDS). Based on the modified benchmark model proposed in [24], we generated 100 simulation data sets under each combinatorial parameter setting, where each data set consists of *N* = 50 samples and each sample contains *M* = 5,000 probes. The log-ratios at every probe are simulated by a mixture of normal and tumor genomes, with the normal cell fraction *λ* being randomly drawn from a uniform distribution $U\left(0.2,0.8\right)$. Zero-mean Gaussian noise is then added to each sample with three levels of standard deviation *σ* randomly drawn from uniform distributions $U\left(0.2,0.4\right)$$U\left(0.4,0.6\right)$, and $U\left(0.6,0.8\right)$. To make the simulations more realistic, for each simulated sample genome, we insert 2 to 10 randomly located background CNA regions with the lengths varying from 10 to 50 probes. There are three ‘amplification’ (*L* = 30, 20, 10) and one ‘deletion’ (*L* = 20) ground truth SCAs embedded in each of the simulation data sets with a baseline frequency *ω* = 0.1. The copy numbers associated with amplification SCAs are 3, 4 and 5, and deletion SCAs are 0 and 1. In our simulation software, we use two parameters *β*_*L*_ and *β*_*ω*_ to modify the length and frequency of these SCAs. Other parameter settings include *θ*_*ρ*_ = 0.75, *θ*_amplification_ = 0.1 and *θ*_deletion_ = −0.1 (default setting by GISTIC and CBS) for defining CNAs probes and units. Based on the estimated true positive rate (TPR) and corresponding FPR at different significance levels, Figure 3 presents ROC curves of SAIC and peer methods derived from the simulation studies. These comparative experimental results consistently show that SAIC outperforms the peer methods in terms of larger areas *A*_*z*_ under the ROC curves or increased sensitivity at low FPR. More simulation studies are given in Additional file 1, where we report the power in detecting the boundaries of SCAs by these methods, and once again, showing outperformance of SAIC as compared to the peer methods [3,14].

### Application to four real cancer copy number data sets

We applied SAIC to four real cancer copy number data sets and identified many SCAs that encompass established or potentially novel cancer ‘driver’ genes. The data sets are from ovarian cancer [26,27], prostate cancer [8,18], lung adenocarcinoma [1,7], and glioblastoma [1,3]. Due to their distinct biological functions in cancer development, SAIC analyzes separately chromosomes 1–22 and chromosome X/Y. To account for the different background CNA rates across chromosomes, we identify SCAs by performing SAIC on individual chromosomes. Other parameter settings include *T* = 1000 and *α* = 0.05 (theoretical significance level or FPR/FWER). To provide a somewhat independent verification, we compared the SCAs detected by SAIC with what reproduced by GISTIC on lung adenocarcinoma and glioblastoma data sets that have been previously reported [3,7].

### Results on the ovarian cancer data set

**Figure 4:**
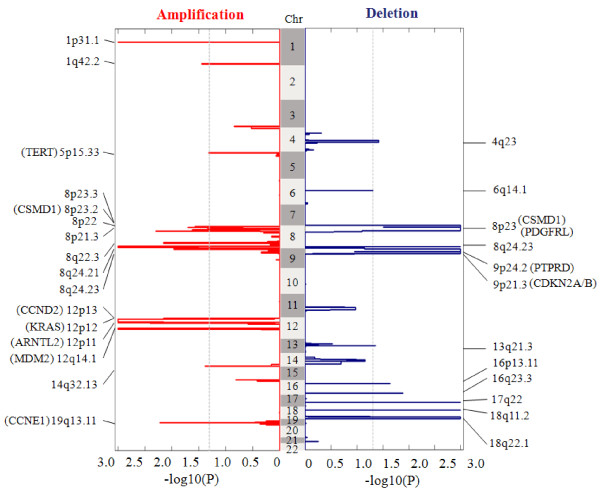
**Genome-wide landscapes of recurrent or sporadic CNAs derived from 63 ovarian cancer samples.** Amplifications and deletions are displayed on the left and right sides, separately, where dashed lines correspond to the significance level _*α* = 0.05_ for calling SCAs.

Our in-house ovarian cancer data set consists of *N* = 63 tumor samples [26-28]. Copy number signals were acquired using the Affymetrix Human Mapping 250 K Sty SNP Array platform [1]. Each sample contains a total of 238,230 probes across the whole genome. Other algorithm parameter settings include *θ*_*ρ*_ = 0.95, *θ*_amplification_ = 0.263 (2.4 copies) and *θ*_deletion_ = −0.322 (1.6 copies) [14]. The genome-wide landscapes (via -log_10_*P*) of recurrent or sporadic CNAs observed in the data sets are given in Figure 4, where amplifications and deletions are separately shown (left and right sides). SAIC detected several SCAs (both amplification and deletion), many of which are biologically plausible and include known oncogenes (e.g., KRAS, CCNE1 and CCND2) and tumor suppressor genes (e.g., CDKN2A and CDKN2B) [26,27,29,30]. Full lists of the genes covered by these SCAs are given in Additional file 2 (ST 2). SAIC also identified many other cancer driver genes within individual chromosomes (ST 3), such as SKIL, CDK4, PIK3CA, PTEN, FGD4, FGFR1.

### Results on the metastatic prostate cancer dataset

**Figure 5:**
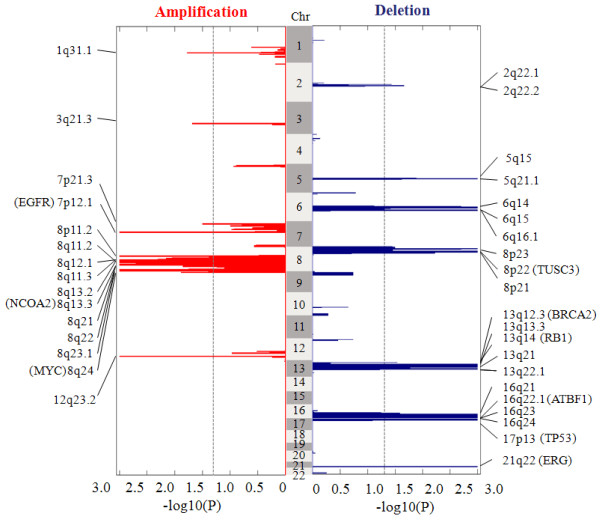
**Genome-wide landscapes of recurrent or sporadic CNAs derived from 13 metastatic prostate cancer samples.** Amplifications and deletions are displayed on the left and right sides, separately, where dashed lines correspond to the significance level *α* = 0.05 for calling SCAs.

Our in-house prostate cancer data set consists of *N* = 55 clustered metastatic tumor samples, obtained from 13 prostate cancer patients. Copy number signals were acquired using Affymetrix Genome-Wide Human SNP Array 6.0 [8,18]. Each sample contains a total of 1,868,857 probes across the whole genome. To discount the potential bias due to imbalanced subject-cluster sampling [8], we chose to analyze the *N* = 13 representative samples and to detect global recurrent CNAs by SAIC. Other algorithm parameter settings include *θ*_*ρ*_ = 0.95, *θ*_amplification_ = 0.263 and *θ*_deletion_ = −0.322, the same as used in analyzing ovarian cancer data. The genome-wide landscape of recurrent or sporadic CNAs observed in metastatic prostate cancer data is given in Figure 5, where SAIC detected 15 amplification SCAs (318 genes) and 21 deletion SCAs (756genes). Full list of the genes covered by these SCAs are given in Additional file 3 (ST 4). Many of these genes are cancer related (e.g., EGFR, BRCA2, TP53, ATBF1, MYC and RB1). In individual chromosome analysis of the data set, SAIC identified many other SCAs involved with cancer driver genes, such as PTEN (ST 5).

### Results on the lung adenocarcinoma and glioblastoma datasets

**Figure 6:**
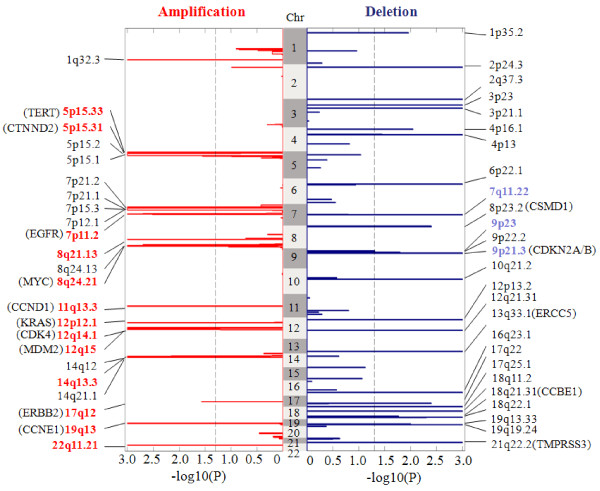
**Genome-wide landscapes of recurrent or sporadic CNAs derived from 371 lung adenocarcinoma samples.** Amplifications and deletions are displayed on the left and right sides, separately, where dashed lines correspond to the significance level _*α* = 0.05_ for calling SCAs.

**Figure 7:**
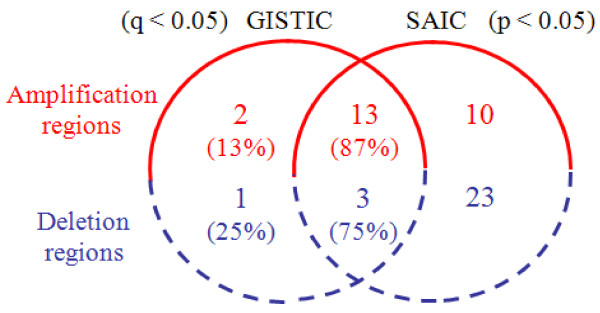
Venn diagram on the numbers of common and distinct focal SCAs detected by SAIC and GISTIC in the lung adenocarcinoma samples.

The lung adenocarcinoma data set consists of *N* = 371 tumor samples, publicly available at http://www.broad.mit.edu/cancer/pub/tsp[7]. Copy number signals were acquired using Affymetrix 250K Sty SNP Array, where each sample contains a total of 216,327 probes across the whole genome [7]. To assure the general comparability of the results produced by SAIC and GISTIC, we adopted similar algorithm parameter settings used by GISTIC for detecting focal SCAs: *θ*_amplification_ = 0.848 and *θ*_deletion_ = −1.15, in addition to *θ*_*ρ*_ = 0.9. The genome-wide landscape of recurrent or sporadic CNAs observed in lung adenocarcinoma data is given in Figure 6, where SAIC detected 23 amplification SCAs and 26 deletion SCAs (after combining some of 98 recurrent CNAs within the same cytobands). Full list of the genes covered by these SCAs is given in Additional file 4 (ST 6). The Venn diagram in Figure 7 reveals the numbers of common and distinctive SCAs detected by SAIC and GISTIC. It can be seen that SAIC successfully detected most (87% amplification and 75% deletion regions) of the SCAs that have been detected by GISTIC, while also revealing many additional SCAs (10 amplification and 23 deletion regions) [7]. In addition, the result from within-chromosome analysis of the data set is listed in Additional file 4 (ST 7).

**Figure 8:**
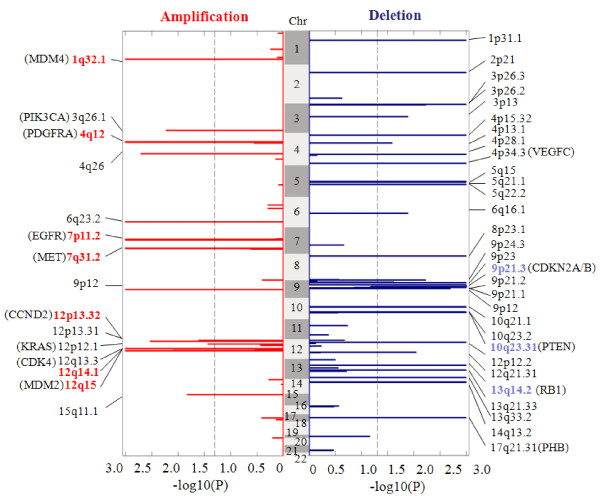
**Genome-wide landscapes of recurrent or sporadic CNAs derived from 141 glioblastoma samples.** Amplifications and deletions are displayed on the left and right sides, separately, where dashed lines correspond to the significance level _*α* = 0.05_ for calling SCAs.

**Figure 9:**
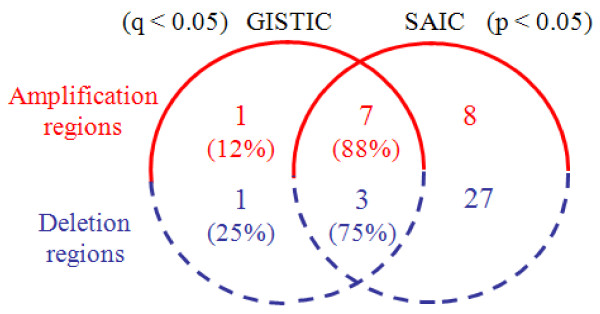
Venn diagram on the numbers of common and distinct focal SCAs detected by SAIC and GISTIC in the glioblastoma samples.

The glioblastoma data set consists of *N* = 141 tumor samples, publicly available at http://www.broad.mit.edu/cancer/pub/GISTIC, where each sample contains a total of 115,593 probes across the whole genome [3]. Once again, we adopted the similar algorithm parameter settings used by GISTIC for detecting focal SCAs. The genome-wide landscape of recurrent or sporadic CNAs observed in glioblastoma data is given in Figure 8, where SAIC detected 15 amplification SCAs and 30 deletion SCAs (after combining some of 67 recurrent CNAs within the same cytobands). Full list of the genes covered by these SCAs are given in Additional file 5 (ST 8). The Venn diagram in Figure 9 reveals the numbers of common and distinctive SCAs detected by SAIC and GISTIC. It can be seen that SAIC successfully detected most (88% amplification and 75% deletion regions) of the SCAs that have been detected by GISTIC, while it also revealed many additional SCAs (8 amplification and 27 deletion regions) [3]. In addition, the result from within-chromosome analysis of the data set is listed in Additional file 5 (ST 9).

The common SCAs regions (e.g., 7p11.2, 12p12.1, 9p21.3, etc.) are highly consistent with previous reports, and largely encompass well-known oncogenes or tumor suppressor genes. For example, EGFR (epidermal growth factor receptor) is an oncogene within 7p11.2 whose mutations or amplifications have been shown to contribute to uncontrolled cell division (a predisposition for cancer) [31]. Many additional SCAs regions (e.g., 8p23.2, 21q22.2) contain or adjacent to disease-related genes (e.g., CSMD1 and TMPRSS3) and may warrant further study.

## Discussion

SAIC is similar to many peer methods in that it assesses statistical significance of SCAs using a permutation-based null distribution [9,12,14-16]. However, in contrast to the existing procedures, the CNA units used by SAIC preserve the essential correlation structures of serial probes whose estimated average correlation coefficient can be as high as 0.985 [32]. Moreover, by automatically adjusting P-values for multiple comparisons [33,34] and iteratively re-estimating the null distribution exclusive of detected SCAs [9], SAIC can preserve the intrinsic false positive rate, without compromising detection power to resort to sometimes overly conservative schemes [3,14-16]. Theoretic analysis and extensive experimental results show that SAIC preserves both type 1 error and detection power, see Tables 1,2. Furthermore, the novel concept of CNA unit and associated scoring and permutation scheme neatly parallels many considerations in the revised GISTIC2.0 [14], for example, serial probes covering driver events should be more highly correlated than probes covering only passengers and thus more likely to identify the target genes. The flexible length-adaptive significance assessment of CNA units via Eq. (4) automatically accounts for distinct background rates according to their lengths and thus more likely to detect independent SCAs.

As for the *θ*_amplification_ and *θ*_deletion_ parameters in the SAIC algorithm, there is no general guideline about how to select their values [14], since different types of cancers usually have different rates and magnitudes of background CNAs [14,26,35]. In addition, various degrees of normal cell contamination [18] and intratumor heterogeneity [35,36] occur in many samples and these further complicate the selection of parameter values. Practically, lower thresholds were used to define broad (arm-level) CNAs while higher thresholds were used to define focal CNAs [3,14]. A newly proposed strategy is to apply joint magnitude-length thresholds [14] and to correct normal cell contamination using BACOM [18]. Since our main objective here is to identify focal CNAs, we have largely adopted the same strategy used in [3,14], i.e., we used relatively higher thresholds to define focal CNAs for subsequent analyses. Specifically, based on the observation that the magnitude of CNAs in ovarian and prostate cancers is relatively low, we used relatively lower and commonly used thresholds (2.0 ± 0.4), i.e., 2.4 copies for amplification and 1.6 copies for deletion. In contrast, on the datasets of lung adenocarcinoma and glioblastoma, we applied relatively higher thresholds (2.0 + 1.6, 2.0–1.1), i.e., 3.6 copies for amplification and 0.9 copies for deletion, that are similar to the thresholds used by GISTIC algorithms [3,14].

Similar situation occurs to the selection of *θ*_*ρ*_ in defining CNA units [9]. Lower values of *θ*_*ρ*_ often produce longer CNA units while higher values of *θ*_*ρ*_ often produce shorter CNA units. It has been reported that the average successive probe correlation of the segmented data can be as high as 0.985 [9,32]. In our experience in analyzing real cancer datasets, a value of $\theta_{\rho }$ taking between 0.7 and 0.95 would be a suitable choice.

It is important to note that the general conclusion on the relative performance of our SAIC and peer methods, at least based on the extensive simulation studies, remains largely true. We have used the same parameter values in all methods so that a fair comparison on their relative performances can be assured. Based on our analysis of real datasets using current parameter settings, it appears that SAIC performs well when compared to peer methods. In addition, the results of extensive simulation studies, performed under a variety of probe correlation schemes, show that SAIC preserves well the expected type 1 error, even when the probes follow non-stationary correlation structures similar to those found in real data [9].

SAIC currently can perform either genome-wide (except X/Y chromosome due to its distinct biological role) or chromosome-based CNA unit permutations. In the application of SAIC to real cancer data sets, we performed genome-wide, autosome-based, and X/Y-chromosome-based permutations. The combined results from using different permutation schemes contain more SCAs that may involve novel cancer driver genes. By exploiting the novel concepts of CNA probe, CNA unit, and multiscale permutation, experimental results show that SAIC can accurately detect the boundaries of SCAs with different lengths, see Additional file 1.

We have also performed simulation studies (data not shown) that indicate that detection power of SAIC can be further improved by correcting for normal tissue contamination using a recently developed BACOM method [18]. However, the current version of BACOM requires paired tumor-normal sampling, availability of two-channel signals, and existence of deletion CNAs. Thus, we leave the combination of SAIC and BACOM as an extension for future research.

## Conclusions

We have presented a novel approach to accurately detect significant recurrent CNAs in cancer genomes which is both statistically-principled and which, as illustrated by real examples, can be very effective at revealing SCAs within data. The concepts of CNA unit and iterative permutation are relatively simple to interpret, yet still convey considerable novel mathematical insights into data structure and bias correction.

It is worth noting that there are three novel features associated with SAIC. First, we define CNA unit to capture the intrinsic correlation structure in copy number data. Second, we perform iterative SCA-exclusive permutation to produce an unbiased null distribution. Third, we apply SAIC to real cancer copy number datasets and detect most previously reported SCAs covering well-known cancer genes.

Two important pending issues with the present algorithm are the expected significant impact of intratumor heterogeneity and normal cell contamination [18,35,36]. We are currently investigating applications of BACOM based normal cell correction [18] and hierarchical bi-clustering that optimize critical steps such as the selection of various thresholds and identification of subtype-specific copy number alterations.

## Appendix A

*Proof of theorem 1.* Let α’ be the significance level used in each iteration to detect SCAs in Algorithm 2. Under the truth converging null distribution, we have

(5) $$\mathrm{Pr}\left({\text{SCA}}^{\left(r\right)}\text{= 'yes'}|{\text{SCA}}^{\left(r-1\right)}\text{= 'yes'}\right)=\alpha '\text{,}$$

for iterations r=1,2,…,∞ since SAIC assesses the ‘new’ SCAs at the *r*th iteration conditional on having found the ‘existing’ SCAs at the (*r*-1)th iteration.Considering

(6) $$\begin{array}{l} \;\mathrm{Pr}\left({\text{SCA}}^{\left(2\right)}\text{= 'yes'}\right)\\ \text{=}\mathrm{Pr}\left({\text{SCA}}^{\left(2\right)}{\text{= 'yes',SCA}}^{\left(1\right)}\text{= 'yes'}\right)\\ =\mathrm{Pr}\left({\text{SCA}}^{\left(2\right)}\text{= 'yes'}|{\text{SCA}}^{\left(1\right)}\text{= 'yes'}\right)\mathrm{Pr}\left({\text{SCA}}^{\left(1\right)}\text{= 'yes'}\right)\\ =\alpha '\cdot \alpha '=\alpha {'}^{2}\text{.} \end{array}$$

Therefore for the *r*th iteration,

(7) $$\begin{array}{l} \;\mathrm{Pr}\left({\text{SCA}}^{\left(r\right)}\text{= 'yes'}\right)\\ \text{=}\mathrm{Pr}\left({\text{SCA}}^{\left(r\right)}{\text{= 'yes',SCA}}^{\left(r-1\right)}\text{= 'yes',}\ldots {\text{,SCA}}^{\left(1\right)}\text{= 'yes'}\right)\\ =\mathrm{Pr}\left({\text{SCA}}^{\left(r\right)}\text{= 'yes'}|{\text{SCA}}^{\left(r-1\right)}{\text{= 'yes',SCA}}^{\left(r-2\right)}\text{= 'yes',}\ldots ,{\text{SCA}}^{\left(1\right)}\text{= 'yes'}\right)\\ \cdot \mathrm{Pr}\left({\text{SCA}}^{\left(r-1\right)}\text{= 'yes'}|{\text{SCA}}^{\left(r-2\right)}\text{= 'yes'},{\text{SCA}}^{\left(r-3\right)}\text{= 'yes',}\ldots {\text{,SCA}}^{\left(1\right)}\text{= 'yes}\right)\\ \cdot \ldots \cdot \mathrm{Pr}\left({\text{SCA}}^{\left(2\right)}\text{= 'yes'}|{\text{SCA}}^{\left(1\right)}\text{= 'yes'}\right)\mathrm{Pr}\left({\text{SCA}}^{\left(1\right)}\text{= 'yes'}\right)\\ =\mathrm{Pr}\left({\text{SCA}}^{\left(r\right)}\text{= 'yes'}|{\text{SCA}}^{\left(r-1\right)}\text{= 'yes'}\right)\cdot \mathrm{Pr}\left({\text{SCA}}^{\left(r-1\right)}\text{= 'yes'}|{\text{SCA}}^{\left(r-2\right)}\text{= 'yes'}\right)\\ \cdot \ldots \cdot \mathrm{Pr}\left({\text{SCA}}^{\left(2\right)}\text{= 'yes'}|{\text{SCA}}^{\left(1\right)}\text{= 'yes'}\right)\mathrm{Pr}\left({\text{SCA}}^{\left(1\right)}\text{= 'yes'}\right)\\ =\alpha '\cdot \alpha '\cdot \alpha '\cdots \alpha '=\alpha {'}^{r}\text{.} \end{array}$$

The rationale behind the above derivation is that ${\text{SCA}}^{\left(r-1\right)}\text{= 'yes'}$ already implies ${\text{SCA}}^{\left(r-2\right)}\text{= 'yes',}\ldots ,{\text{SCA}}^{\left(1\right)}\text{= 'yes'}$. In other words, we have

(8) $$\mathrm{Pr}\left({\text{SCA}}^{\left(r\right)}\text{= 'yes'}\right)\text{=}\mathrm{Pr}\left({\text{SCA}}^{\left(r\right)}{\text{= 'yes',SCA}}^{\left(r-1\right)}\text{= 'yes',}\ldots ,{\text{SCA}}^{\left(1\right)}\text{= 'yes'}\right)$$

and

(9) $$\begin{array}{l} \mathrm{Pr}\left({\text{SCA}}^{\left(r\right)}\text{= 'yes'}|{\text{SCA}}^{\left(r-1\right)}{\text{= 'yes',SCA}}^{\left(r-2\right)}\text{= 'yes',}\ldots ,{\text{SCA}}^{\left(1\right)}\text{= 'yes'}\right)\\ =\mathrm{Pr}\left({\text{SCA}}^{\left(r\right)}\text{= 'yes'}|{\text{SCA}}^{\left(r-1\right)}\text{= 'yes'}\right) \end{array}\text{.}$$

Let *α* be the targeted FPR, we have

(10) $$\begin{array}{l} \;\alpha \text{=}\sum_{r=1}^{\infty }\mathrm{Pr}\left({\text{SCA}}^{\left(r\right)}\text{= 'yes'}\right)\\ =\alpha '+\alpha {'}^{2}+\ldots +\alpha {'}^{r}+\ldots =\frac{\alpha '}{1-\alpha '}\text{,}\;\left(\alpha '<1\right)\text{.} \end{array}$$

Accordingly, we have $\alpha '=\alpha /\left(1+\alpha \right)$.

## Additional files

## Competing interests

The authors declared that they have no competing interests.

## Authors’ contributions

XY, GY and YW participated in the design of concepts and methods. XY and GY developed the permutation strategy and CNA simulation algorithm. XY implemented the C++ code. RRW implemented the R code of GISTIC. GY, XY and XH analyzed and evaluated the algorithm. XH and YW constructed and proved Theorem 1. YW, XY and GY drafted the manuscript. IMS and EPH interpreted the results on real cancer data. JZ, RC and EPH help edited the manuscript. YW, RC and ZZ conceived of the study, participated in its design and coordination, and helped edited the paper. All authors read and approved the final manuscript.

## Supplementary Material

Additional file 1 **Table S1.** Comparative detection rates of ground truth SCA boundaries by STAC, GISTIC, KC-SMART, CMDS, and SAIC for simulation data sets under various model parameter settings. The results are calculated based on 100 replications for each of the parameter settings and using p-value (or q-value) cutoff threshold <0.05. Click here for file

Additional file 2 **Table S2 and Table S3.** Details about the implicated SCAs and full list of genes covered by these SCAs, derived from the ovarian cancer data set. Click here for file

Additional file 3 **Table S4 and Table S5. **Details about the implicated SCAs and full list of genes covered by these SCAs, derived from the prostate cancer data set. Click here for file

Additional file 4 **Table 6 and Suplementary Table 7. **Details about the implicated SCAs and full list of genes covered by these SCAs, derived from the lung adenocarcinoma data set. Click here for file

Additional file 5 **Table S8 and Table S9.** Details about the implicated SCAs and full list of genes covered by these SCAs, derived from the glioblastoma data set. Click here for file
