# Supplementary material for: Genome-wide identification of significant aberrations in cancer genome
Source: BMC Genomics. 2012 Jul 27;13:342. doi: 10.1186/1471-2164-13-342 (PMC3428679; doi:10.1186/1471-2164-13-342)
Supplement: Additional file 1 — Table S1. Comparative detection rates of ground truth SCA boundaries by STAC, GISTIC, KC-SMART, CMDS, and SAIC for simulation data sets under various model parameter settings. The results are calculated based on 100 replications for each of the parameter settings and using p-value (or q-value) cutoff threshold <0.05. [file 1471-2164-13-342-S1.doc]

**Supplementary Table 1.** Comparative detection rates of ground truth SCA boundaries by STAC, GISTIC, KC-SMART, CMDS, and SAIC for simulation data sets under various model parameter settings. The results are calculated based on 100 replications for each of the parameter settings and using p-value (or q-value) cutoff threshold <0.05. (Statistic/window: testing statistics or window lengths. Start: Start point of SCAs. End: End point of SCAs. Both: Start and End points of SCAs.)

1. *β_L_* = 1, *β_ω_* = 1, *σ*= (0.2, 0.4)

| Method | Statistic/  window | SCA 1 | | | SCA 2 | | | SCA 3 | | | SCA 4 | | |
| --- | --- | --- | --- | --- | --- | --- | --- | --- | --- | --- | --- | --- | --- |
|  |  | Start | End | Both | Start | End | Both | Start | End | Both | Start | End | Both |
| STAC | frequency | 0.43 | 0.48 | 0.32 | **1.00** | **1.00** | **1.00** | **1.00** | **1.00** | **1.00** | 0.99 | 0.98 | 0.97 |
|  | footprint | **0.84** | **0.87** | **0.82** | 0.94 | 0.93 | 0.93 | 0.94 | 0.94 | 0.94 | 0.91 | 0.92 | 0.91 |
| GISTIC | G-score | 0.57 | 0.61 | 0.50 | **1.00** | 0.99 | 0.99 | **1.00** | **1.00** | **1.00** | **1.00** | **1.00** | **1.00** |
| KC-SMART | KC-score | 0.06 | 0.01 | 0.00 | 0.00 | 0.00 | 0.00 | 0.00 | 0.00 | 0.00 | 0.00 | 0.00 | 0.00 |
| CMDS | *w*=10 | 0.34 | 0.38 | 0.15 | 0.00 | 0.01 | 0.00 | 0.00 | 0.00 | 0.00 | 0.06 | 0.12 | 0.02 |
|  | *w*=20 | 0.04 | 0.02 | 0.00 | 0.00 | 0.00 | 0.00 | 0.00 | 0.00 | 0.00 | 0.00 | 0.00 | 0.00 |
|  | *w*=30 | 0.02 | 0.08 | 0.01 | 0.00 | 0.00 | 0.00 | 0.00 | 0.00 | 0.00 | 0.00 | 0.00 | 0.00 |
| SAIC | U-score | 0.77 | 0.76 | 0.75 | **1.00** | 0.99 | 0.99 | **1.00** | **1.00** | **1.00** | **1.00** | **1.00** | **1.00** |

1. *β_L_* = 1, *β_ω_* = 1, *σ*= (0.4, 0.6)

| Method | Statistic/  window | SCA 1 | | | SCA 2 | | | SCA 3 | | | SCA 4 | | |
| --- | --- | --- | --- | --- | --- | --- | --- | --- | --- | --- | --- | --- | --- |
|  |  | Start | End | Both | Start | End | Both | Start | End | Both | Start | End | Both |
| STAC | frequency | 0.00 | 0.00 | 0.00 | 0.02 | 0.02 | 0.00 | 0.08 | 0.07 | 0.04 | 0.02 | 0.01 | 0.00 |
|  | footprint | 0.07 | 0.09 | 0.03 | 0.57 | 0.47 | 0.35 | 0.76 | 0.80 | 0.75 | 0.25 | 0.26 | 0.22 |
| GISTIC | G-score | 0.00 | **0.29** | 0.00 | 0.17 | 0.18 | 0.17 | 0.15 | 0.88 | 0.15 | **0.64** | 0.60 | 0.55 |
| KC-SMART | KC-score | 0.07 | 0.05 | 0.00 | 0.09 | 0.03 | 0.00 | 0.05 | 0.00 | 0.00 | 0.04 | 0.00 | 0.00 |
| CMDS | *w*=10 | 0.03 | 0.08 | 0.00 | 0.19 | 0.14 | 0.01 | 0.33 | 0.27 | 0.08 | 0.14 | 0.14 | 0.00 |
|  | *w*=20 | 0.03 | 0.05 | 0.00 | 0.13 | 0.14 | 0.05 | 0.03 | 0.03 | 0.00 | 0.09 | 0.12 | 0.00 |
|  | *w*=30 | 0.01 | 0.04 | 0.00 | 0.10 | 0.12 | 0.01 | 0.05 | 0.01 | 0.00 | 0.14 | 0.18 | 0.02 |
| SAIC | U-score | **0.21** | 0.21 | **0.11** | **0.79** | **0.82** | **0.67** | **0.87** | **0.95** | **0.86** | 0.62 | **0.63** | **0.59** |

1. *β_L_* = 1, *β_ω_* = 1, *σ*= (0.6, 0.8)

| Method | Statistic/  window | SCA 1 | | | SCA 2 | | | SCA 3 | | | SCA 4 | | |
| --- | --- | --- | --- | --- | --- | --- | --- | --- | --- | --- | --- | --- | --- |
|  |  | Start | End | Both | Start | End | Both | Start | End | Both | Start | End | Both |
| STAC | frequency | 0.00 | 0.00 | 0.00 | 0.05 | 0.08 | 0.02 | 0.07 | 0.05 | 0.04 | 0.00 | 0.00 | 0.00 |
|  | footprint | 0.04 | **0.04** | **0.01** | 0.37 | 0.31 | 0.20 | 0.39 | 0.41 | 0.31 | 0.11 | 0.13 | 0.09 |
| GISTIC | G-score | 0.03 | 0.01 | 0.00 | 0.06 | 0.05 | 0.05 | 0.04 | 0.12 | 0.04 | **0.47** | 0.45 | 0.35 |
| KC-SMART | KC-score | 0.02 | 0.00 | 0.00 | 0.06 | 0.02 | 0.00 | 0.07 | 0.01 | 0.00 | 0.17 | 0.01 | 0.00 |
| CMDS | *w*=10 | **0.10** | 0.01 | 0.00 | 0.13 | 0.17 | 0.03 | 0.23 | 0.18 | 0.03 | 0.08 | 0.13 | 0.00 |
|  | *w*=20 | 0.04 | 0.05 | 0.00 | 0.07 | 0.18 | 0.04 | 0.01 | 0.06 | 0.00 | 0.05 | 0.13 | 0.01 |
|  | *w*=30 | 0.03 | 0.03 | 0.00 | 0.05 | 0.13 | 0.00 | 0.02 | 0.05 | 0.00 | 0.08 | 0.07 | 0.00 |
| SAIC | U-score | 0.03 | 0.01 | 0.00 | **0.34** | **0.33** | **0.22** | **0.43** | **0.43** | **0.35** | 0.43 | **0.49** | **0.43** |

1. *β_L_* = 1, *β_ω_* = 1.5, *σ*= (0.2, 0.4)

| Method | Statistic/  window | SCA 1 | | | SCA 2 | | | SCA 3 | | | SCA 4 | | |
| --- | --- | --- | --- | --- | --- | --- | --- | --- | --- | --- | --- | --- | --- |
|  |  | Start | End | Both | Start | End | Both | Start | End | Both | Start | End | Both |
| STAC | frequency | 0.10 | 0.05 | 0.01 | 0.11 | 0.16 | 0.04 | 0.18 | 0.20 | 0.11 | 0.09 | 0.08 | 0.02 |
|  | footprint | 0.86 | 0.73 | 0.68 | 0.97 | **0.99** | 0.96 | 0.99 | 0.99 | 0.99 | 0.76 | 0.76 | 0.75 |
| GISTIC | G-score | **0.99** | 0.23 | 0.23 | **1.00** | 0.83 | 0.83 | **1.00** | 0.68 | 0.68 | 0.88 | 0.80 | 0.73 |
| KC-SMART | KC-score | 0.03 | 0.01 | 0.00 | 0.00 | 0.00 | 0.00 | 0.00 | 0.00 | 0.00 | 0.02 | 0.01 | 0.00 |
| CMDS | *w*=10 | 0.03 | 0.03 | 0.00 | 0.08 | 0.21 | 0.00 | 0.41 | 0.36 | 0.17 | 0.13 | 0.11 | 0.01 |
|  | *w*=20 | 0.01 | 0.01 | 0.00 | 0.14 | 0.14 | 0.00 | 0.04 | 0.02 | 0.00 | 0.13 | 0.15 | 0.04 |
|  | *w*=30 | 0.03 | 0.03 | 0.00 | 0.11 | 0.11 | 0.01 | 0.00 | 0.00 | 0.00 | 0.10 | 0.15 | 0.03 |
| SAIC | U-score | 0.92 | **0.89** | **0.83** | 0.99 | **0.99** | **0.98** | **1.00** | **1.00** | **1.00** | **0.89** | **0.90** | **0.88** |

1. *β_L_* = 1, *β_ω_* = 1.5, *σ*= (0.4, 0.6)

| Method | Statistic/  window | SCA 1 | | | SCA 2 | | | SCA 3 | | | SCA 4 | | |
| --- | --- | --- | --- | --- | --- | --- | --- | --- | --- | --- | --- | --- | --- |
|  |  | Start | End | Both | Start | End | Both | Start | End | Both | Start | End | Both |
| STAC | frequency | 0.04 | 0.04 | 0.01 | 0.27 | 0.30 | 0.13 | 0.30 | 0.25 | 0.19 | 0.04 | 0.04 | 0.00 |
|  | footprint | 0.24 | 0.27 | 0.11 | 0.71 | 0.75 | 0.61 | 0.91 | 0.89 | 0.86 | 0.59 | 0.59 | 0.55 |
| GISTIC | G-score | **0.45** | 0.20 | 0.20 | 0.05 | 0.15 | 0.05 | 0.02 | 0.11 | 0.02 | 0.36 | 0.33 | 0.32 |
| KC-SMART | KC-score | 0.14 | 0.03 | 0.00 | 0.06 | 0.00 | 0.00 | 0.01 | 0.01 | 0.00 | 0.01 | 0.00 | 0.00 |
| CMDS | *w*=10 | 0.02 | 0.03 | 0.00 | 0.19 | 0.25 | 0.06 | 0.31 | 0.27 | 0.09 | 0.15 | 0.17 | 0.01 |
|  | *w*=20 | 0.01 | 0.05 | 0.00 | 0.05 | 0.09 | 0.00 | 0.04 | 0.02 | 0.00 | 0.10 | 0.09 | 0.01 |
|  | *w*=30 | 0.00 | 0.05 | 0.00 | 0.05 | 0.09 | 0.00 | 0.04 | 0.02 | 0.00 | 0.10 | 0.09 | 0.01 |
| SAIC | U-score | 0.42 | **0.35** | **0.21** | **0.85** | 0.88 | **0.76** | **0.97** | **0.97** | **0.94** | **0.83** | **0.77** | **0.75** |

1. *β_L_* = 1, *β_ω_* = 1.5, *σ*= (0.6, 0.8)

| Method | Statistic/  window | SCA 1 | | | SCA 2 | | | SCA 3 | | | SCA 4 | | |
| --- | --- | --- | --- | --- | --- | --- | --- | --- | --- | --- | --- | --- | --- |
|  |  | Start | End | Both | Start | End | Both | Start | End | Both | Start | End | Both |
| STAC | frequency | 0.00 | 0.00 | 0.00 | 0.09 | 0.18 | 0.06 | 0.27 | 0.22 | 0.17 | 0.03 | 0.03 | 0.01 |
|  | footprint | 0.05 | 0.05 | 0.03 | 0.47 | 0.31 | 0.23 | 0.55 | 0.53 | 0.42 | 0.32 | 0.30 | 0.24 |
| GISTIC | G-score | 0.04 | 0.00 | 0.00 | 0.27 | 0.04 | 0.04 | 0.04 | 0.04 | 0.04 | 0.45 | 0.33 | 0.31 |
| KC-SMART | KC-score | 0.04 | 0.00 | 0.00 | 0.14 | 0.01 | 0.00 | 0.11 | 0.01 | 0.00 | 0.05 | 0.05 | 0.01 |
| CMDS | *w*=10 | 0.02 | 0.10 | 0.00 | 0.17 | 0.18 | 0.04 | 0.28 | 0.24 | 0.09 | 0.18 | 0.16 | 0.03 |
|  | *w*=20 | 0.05 | 0.05 | 0.00 | 0.09 | 0.17 | 0.02 | 0.00 | 0.02 | 0.00 | 0.13 | 0.11 | 0.01 |
|  | *w*=30 | 0.02 | 0.02 | 0.00 | 0.06 | 0.08 | 0.00 | 0.03 | 0.07 | 0.01 | 0.11 | 0.06 | 0.01 |
| SAIC | U-score | **0.12** | **0.11** | **0.07** | **0.63** | **0.50** | **0.34** | **0.70** | **0.67** | 0.50 | **0.64** | **0.57** | **0.54** |

1. *β_L_* = 1.5, *β_ω_* = 1, *σ*= (0.2, 0.4)

| Method | Statistic/  window | SCA 1 | | | SCA 2 | | | SCA 3 | | | SCA 4 | | |
| --- | --- | --- | --- | --- | --- | --- | --- | --- | --- | --- | --- | --- | --- |
|  |  | Start | End | Both | Start | End | Both | Start | End | Both | Start | End | Both |
| STAC | frequency | 0.01 | 0.01 | 0.00 | 0.01 | 0.00 | 0.00 | 0.04 | 0.02 | 000 | 0.01 | 0.02 | 0.00 |
|  | footprint | 0.52 | 0.48 | 0.37 | 0.76 | 0.76 | 0.72 | 0.83 | 0.82 | 0.81 | 0.52 | 0.53 | 0.51 |
| GISTIC | G-score | 0.24 | 0.22 | 0.12 | 0.90 | 0.86 | 0.81 | 0.97 | **1.00** | 0.97 | 0.55 | 0.54 | 0.43 |
| KC-SMART | KC-score | 0.09 | 0.10 | 0.01 | 0.01 | 0.00 | 0.00 | 0.00 | 0.00 | 0.00 | 0.00 | 0.00 | 0.00 |
| CMDS | *w*=10 | 0.00 | 0.03 | 0.00 | 0.11 | 0.09 | 0.00 | 0.21 | 0.23 | 0.07 | 0.07 | 0.07 | 0.01 |
|  | *w*=20 | 0.02 | 0.05 | 0.00 | 0.13 | 0.15 | 0.01 | 0.23 | 0.29 | 0.08 | 0.08 | 0.06 | 0.02 |
|  | *w*=30 | 0.02 | 0.02 | 0.00 | 0.13 | 0.12 | 0.00 | 0.01 | 0.01 | 0.00 | 0.12 | 0.12 | 0.03 |
| SAIC | U-score | **0.76** | **0.77** | **0.65** | **0.99** | **0.98** | **0.97** | **1.00** | **1.00** | **1.00** | **0.78** | **0.79** | **0.76** |

1. *β_L_* = 1.5, *β_ω_* = 1, *σ*= (0.4, 0.6)

| Method | Statistic/  window | SCA 1 | | | SCA 2 | | | SCA 3 | | | SCA 4 | | |
| --- | --- | --- | --- | --- | --- | --- | --- | --- | --- | --- | --- | --- | --- |
|  |  | Start | End | Both | Start | End | Both | Start | End | Both | Start | End | Both |
| STAC | frequency | 0.00 | 0.01 | 0.00 | 0.05 | 0.03 | 0.00 | 0.07 | 0.10 | 0.02 | 0.02 | 0.00 | 0.00 |
|  | footprint | 0.25 | 0.21 | 0.10 | 0.64 | 0.56 | 0.44 | 0.75 | 0.77 | 0.69 | 0.40 | 0.40 | 0.34 |
| GISTIC | G-score | 0.10 | 0.06 | 0.02 | 0.77 | 0.71 | 0.59 | 0.92 | 0.89 | 0.82 | 0.53 | 0.43 | 0.36 |
| KC-SMART | KC-score | 0.06 | 0.17 | 0.02 | 0.06 | 0.01 | 0.00 | 0.02 | 0.00 | 0.00 | 0.03 | 0.01 | 0.00 |
| CMDS | *w*=10 | 0.01 | 0.02 | 0.00 | 0.19 | 0.18 | 0.03 | 0.36 | 0.33 | 0.13 | 0.10 | 0.13 | 0.03 |
|  | *w*=20 | 0.01 | 0.01 | 0.00 | 0.11 | 0.00 | 0.00 | 0.25 | 0.01 | 0.00 | 0.05 | 0.01 | 0.00 |
|  | *w*=30 | 0.02 | 0.03 | 0.00 | 0.13 | 0.00 | 0.00 | 0.02 | 0.02 | 0.00 | 0.11 | 0.01 | 0.00 |
| SAIC | U-score | **0.40** | **0.41** | **0.25** | **0.80** | **0.80** | **0.65** | **0.94** | **0.93** | **0.89** | **0.74** | **0.70** | **0.69** |

1. *β_L_* = 1.5, *β_ω_* = 1, *σ*= (0.6, 0.8)

| Method | Statistic/  window | SCA 1 | | | SCA 2 | | | SCA 3 | | | SCA 4 | | |
| --- | --- | --- | --- | --- | --- | --- | --- | --- | --- | --- | --- | --- | --- |
|  |  | Start | End | Both | Start | End | Both | Start | End | Both | Start | End | Both |
| STAC | frequency | 0.01 | 0.01 | 0.00 | 0.08 | 0.08 | 0.01 | 0.13 | 0.12 | 0.07 | 0.00 | 0.02 | 0.00 |
|  | footprint | 0.06 | 0.06 | **0.02** | 0.28 | 0.39 | 0.14 | 0.42 | 0.43 | 0.31 | 0.21 | 0.20 | 0.12 |
| GISTIC | G-score | 0.02 | 0.07 | 0.01 | 0.38 | 0.35 | 0.18 | 0.64 | 0.61 | **0.42** | 0.34 | 0.33 | 0.26 |
| KC-SMART | KC-score | 0.02 | 0.05 | 0.00 | 0.05 | 0.04 | 0.00 | 0.05 | 0.14 | 0.00 | 0.08 | 0.02 | 0.00 |
| CMDS | *w*=10 | 0.05 | 0.03 | 0.00 | 0.13 | 0.17 | 0.01 | 0.21 | 0.29 | 0.06 | 0.04 | 0.12 | 0.00 |
|  | *w*=20 | 0.08 | 0.05 | 0.00 | 0.18 | 0.11 | 0.03 | 0.11 | 0.07 | 0.00 | 0.12 | 0.09 | 0.01 |
|  | *w*=30 | 0.03 | 0.03 | 0.00 | 0.06 | 0.09 | 0.02 | 0.03 | 0.02 | 0.00 | 0.04 | 0.13 | 0.01 |
| SAIC | U-score | **0.10** | **0.11** | 0.01 | **0.50** | **0.45** | **0.21** | **0.65** | **0.62** | 0.41 | **0.58** | **0.55** | **0.48** |

1. *β_L_* = 1.5, *β_ω_* = 1.5, *σ*= (0.2, 0.4)

| Method | Statistic/  window | SCA 1 | | | SCA 2 | | | SCA 3 | | | SCA 4 | | |
| --- | --- | --- | --- | --- | --- | --- | --- | --- | --- | --- | --- | --- | --- |
|  |  | Start | End | Both | Start | End | Both | Start | End | Both | Start | End | Both |
| STAC | frequency | 0.04 | 0.05 | 0.00 | 0.25 | 0.16 | 0.04 | 0.20 | 0.17 | 0.12 | 0.08 | 0.10 | 0.00 |
|  | footprint | 0.80 | 0.82 | 0.71 | 0.97 | 0.97 | 0.96 | 0.99 | **1.00** | 0.99 | 0.89 | 0.86 | 0.86 |
| GISTIC | G-score | 0.56 | 0.54 | 0.38 | 0.99 | 0.98 | 0.97 | **1.00** | **1.00** | 1.00 | 0.80 | 0.84 | 0.75 |
| KC-SMART | KC-score | 0.02 | 0.03 | 0.00 | 0.00 | 0.00 | 0.00 | 0.00 | 0.00 | 0.00 | 0.00 | 0.00 | 0.00 |
| CMDS | *w*=10 | 0.04 | 0.04 | 0.00 | 0.11 | 0.12 | 0.00 | 0.38 | 0.29 | 0.12 | 0.08 | 0.11 | 0.03 |
|  | *w*=20 | 0.03 | 0.02 | 0.00 | 0.20 | 0.19 | 0.03 | 0.24 | 0.27 | 0.08 | 0.11 | 0.10 | 0.03 |
|  | *w*=30 | 0.02 | 0.05 | 0.01 | 0.17 | 0.09 | 0.02 | 0.01 | 0.01 | 0.00 | 0.10 | 0.13 | 0.02 |
| SAIC | U-score | **0.83** | **0.91** | **0.78** | **1.00** | **0.99** | **0.99** | **1.00** | **1.00** | **1.00** | **0.94** | **0.97** | **0.94** |

1. *β_L_* = 1.5, *β_ω_* = 1.5, *σ*= (0.4, 0.6)

| Method | Statistic/  window | SCA 1 | | | SCA 2 | | | SCA 3 | | | SCA 4 | | |
| --- | --- | --- | --- | --- | --- | --- | --- | --- | --- | --- | --- | --- | --- |
|  |  | Start | End | Both | Start | End | Both | Start | End | Both | Start | End | Both |
| STAC | frequency | 0.06 | 0.12 | 0.01 | 0.27 | 0.25 | 0.09 | 0.49 | 0.44 | 0.33 | 0.08 | 0.07 | 0.02 |
|  | footprint | 0.33 | 0.34 | 0.18 | 0.79 | 0.77 | 0.66 | 0.97 | 0.95 | 0.94 | 0.57 | 0.51 | 0.46 |
| GISTIC | G-score | 0.16 | 0.19 | 0.06 | **0.88** | 0.87 | 0.76 | 0.95 | 0.95 | 0.91 | 0.60 | 0.59 | 0.45 |
| KC-SMART | KC-score | 0.09 | 0.06 | 0.00 | 0.06 | 0.00 | 0.00 | 0.00 | 0.00 | 0.00 | 0.02 | 0.02 | 0.00 |
| CMDS | *w*=10 | 0.07 | 0.06 | 0.01 | 0.26 | 0.27 | 0.09 | 0.33 | 0.31 | 0.11 | 0.14 | 0.19 | 0.02 |
|  | *w*=20 | 0.05 | 0.04 | 0.00 | 0.22 | 0.17 | 0.02 | 0.03 | 0.08 | 0.00 | 0.13 | 0.13 | 0.02 |
|  | *w*=30 | 0.04 | 0.04 | 0.00 | 0.14 | 0.08 | 0.01 | 0.00 | 0.00 | 0.00 | 0.11 | 0.09 | 0.00 |
| SAIC | U-score | **0.51** | **0.45** | **0.25** | **0.88** | **0.89** | **0.77** | **0.98** | **0.97** | **0.96** | **0.84** | **0.80** | **0.76** |

1. *β_L_* = 1.5, *β_ω_* = 1.5, *σ*= (0.6, 0.8)

| Method | Statistic/  window | SCA 1 | | | SCA 2 | | | SCA 3 | | | SCA 4 | | |
| --- | --- | --- | --- | --- | --- | --- | --- | --- | --- | --- | --- | --- | --- |
|  |  | Start | End | Both | Start | End | Both | Start | End | Both | Start | End | Both |
| STAC | frequency | 0.01 | 0.04 | 0.00 | 0.20 | 0.19 | 0.05 | 0.25 | 0.34 | 0.19 | 0.08 | 0.06 | 0.03 |
|  | footprint | 0.06 | 0.07 | 0.01 | 0.42 | 0.54 | 0.26 | 0.65 | 0.65 | 0.50 | 0.45 | 0.39 | 0.31 |
| GISTIC | G-score | 0.01 | 0.04 | 0.00 | **0.50** | 0.53 | 0.26 | **0.74** | **0.72** | **0.56** | 0.47 | 0.45 | 0.32 |
| KC-SMART | KC-score | 0.02 | 0.05 | 0.00 | 0.06 | 0.04 | 0.00 | 0.07 | 0.06 | 0.01 | 0.09 | 0.00 | 0.00 |
| CMDS | *w*=10 | 0.06 | 0.07 | 0.00 | 0.20 | 0.20 | 0.05 | 0.24 | 0.25 | 0.04 | 0.14 | 0.15 | 0.03 |
|  | *w*=20 | 0.00 | 0.01 | 0.00 | 0.15 | 0.13 | 0.02 | 0.08 | 0.09 | 0.00 | 0.13 | 0.18 | 0.02 |
|  | *w*=30 | 0.05 | 0.03 | 0.00 | 0.11 | 0.08 | 0.00 | 0.02 | 0.01 | 0.00 | 0.08 | 0.10 | 0.00 |
| SAIC | U-score | **0.14** | **0.14** | **0.02** | 0.48 | **0.55** | **0.30** | 0.71 | 0.69 | 0.52 | **0.73** | **0.64** | **0.64** |

1. *β_L_* = 2, *β_ω_* = 1, *σ*= (0.2, 0.4)

| Method | Statistic/  Window | SCA 1 | | | SCA 2 | | | SCA 3 | | | SCA 4 | | |
| --- | --- | --- | --- | --- | --- | --- | --- | --- | --- | --- | --- | --- | --- |
|  |  | Start | End | Both | Start | End | Both | Start | End | Both | Start | End | Both |
| STAC | Frequency | 0.02 | 0.02 | 0.00 | 0.01 | 0.03 | 0.00 | 0.01 | 0.01 | 0.00 | 0.01 | 0.00 | 0.00 |
|  | Footprint | 0.43 | 0.48 | 0.34 | 0.81 | 0.82 | 0.77 | 0.90 | 0.87 | 0.86 | 0.58 | 0.57 | 0.56 |
| GISTIC | G-score | 0.18 | 0.24 | 0.04 | 0.90 | 0.96 | 0.86 | 0.99 | 0.99 | 0.98 | 0.50 | 0.47 | 0.35 |
| KC-SMART | KC-score | 0.11 | 0.02 | 0.00 | 0.00 | 0.00 | 0.00 | 0.00 | 0.00 | 0.00 | 0.04 | 0.00 | 0.00 |
| CMDS | *w*=10 | 0.00 | 0.01 | 0.00 | 0.08 | 0.11 | 0.04 | 0.25 | 0.17 | 0.07 | 0.01 | 0.04 | 0.00 |
|  | *w*=20 | 0.00 | 0.01 | 0.00 | 0.11 | 0.07 | 0.01 | 0.19 | 0.27 | 0.07 | 0.07 | 0.06 | 0.02 |
|  | *w*=30 | 0.02 | 0.00 | 0.00 | 0.11 | 0.13 | 0.03 | 0.13 | 0.08 | 0.01 | 0.09 | 0.17 | 0.04 |
| SAIC | U-score | **0.71** | **0.76** | **0.59** | **0.99** | **1.00** | **0.99** | **1.00** | **1.00** | **1.00** | **0.83** | **0.82** | **0.80** |

1. *β_L_* = 2, *β_ω_* = 1, *σ*= (0.4, 0.6)

| Method | Statistic/  Window | SCA 1 | | | SCA 2 | | | SCA 3 | | | SCA 4 | | |
| --- | --- | --- | --- | --- | --- | --- | --- | --- | --- | --- | --- | --- | --- |
|  |  | Start | End | Both | Start | End | Both | Start | End | Both | Start | End | Both |
| STAC | Frequency | 0.02 | 0.03 | 0.00 | 0.06 | 0.04 | 0.00 | 0.10 | 0.07 | 0.03 | 0.03 | 0.01 | 0.00 |
|  | Footprint | 0.20 | 0.23 | 0.08 | 0.72 | 0.71 | 0.59 | 0.83 | 0.76 | 0.73 | 0.39 | 0.34 | 0.29 |
| GISTIC | G-score | 0.07 | 0.10 | 0.02 | 0.75 | 0.73 | 0.58 | 0.95 | 0.89 | 0.86 | 0.42 | 0.40 | 0.29 |
| KC-SMART | KC-score | 0.10 | 0.03 | 0.01 | 0.07 | 0.06 | 0.01 | 0.02 | 0.00 | 0.00 | 0.04 | 0.03 | 0.01 |
| CMDS | *w*=10 | 0.02 | 0.07 | 0.00 | 0.14 | 0.09 | 0.00 | 0.29 | 0.36 | 0.13 | 0.10 | 0.12 | 0.02 |
|  | *w*=20 | 0.00 | 0.05 | 0.00 | 0.12 | 0.20 | 0.04 | 0.15 | 0.13 | 0.04 | 0.10 | 0.23 | 0.01 |
|  | *w*=30 | 0.01 | 0.03 | 0.00 | 0.10 | 0.13 | 0.01 | 0.00 | 0.03 | 0.00 | 0.12 | 0.11 | 0.03 |
| SAIC | U-score | **0.38** | **0.40** | **0.18** | **0.83** | **0.88** | **0.74** | **0.96** | **0.91** | **0.87** | **0.73** | **0.74** | **0.67** |

1. *β_L_* = 2, *β_ω_* = 1, *σ*= (0.6, 0.8)

| Method | Statistic/  Window | SCA 1 | | | SCA 2 | | | SCA 3 | | | SCA 4 | | |
| --- | --- | --- | --- | --- | --- | --- | --- | --- | --- | --- | --- | --- | --- |
|  |  | Start | End | Both | Start | End | Both | Start | End | Both | Start | End | Both |
| STAC | Frequency | 0.02 | 0.02 | 0.01 | 0.15 | 0.11 | 0.07 | 0.13 | 0.16 | 0.07 | 0.01 | 0.02 | 0.00 |
|  | Footprint | **0.15** | 0.10 | 0.05 | **0.48** | 0.45 | **0.23** | 0.48 | 0.58 | 0.38 | 0.27 | 0.18 | 0.15 |
| GISTIC | G-score | 0.03 | 0.02 | 0.01 | **0.48** | 0.44 | 0.22 | **0.67** | 0.65 | 0.44 | 0.34 | 0.32 | 0.20 |
| KC-SMART | KC-score | 0.02 | 0.01 | 0.00 | 0.06 | 0.03 | 0.00 | 0.06 | 0.00 | 0.00 | 0.07 | 0.03 | 0.00 |
| CMDS | *w*=10 | 0.05 | 0.06 | 0.00 | 0.11 | 0.11 | 0.00 | 0.23 | 0.32 | 0.08 | 0.08 | 0.07 | 0.00 |
|  | *w*=20 | 0.07 | 0.03 | 0.00 | 0.17 | 0.15 | 0.03 | 0.12 | 0.13 | 0.02 | 0.08 | 0.09 | 0.00 |
|  | *w*=30 | 0.04 | 0.01 | 0.00 | 0.10 | 0.11 | 0.00 | 0.04 | 0.09 | 0.01 | 0.03 | 0.07 | 0.00 |
| SAIC | U-score | **0.15** | **0.14** | **0.06** | 0.43 | **0.54** | 0.22 | 0.65 | **0.73** | **0.51** | **0.60** | **0.52** | **0.47** |

1. *β_L_* = 2, *β_ω_* = 1.5, *σ*= (0.2, 0.4)

| Method | Statistic/  window | SCA 1 | | | SCA 2 | | | SCA 3 | | | SCA 4 | | |
| --- | --- | --- | --- | --- | --- | --- | --- | --- | --- | --- | --- | --- | --- |
|  |  | Start | End | Both | Start | End | Both | Start | End | Both | Start | End | Both |
| STAC | frequency | 0.05 | 0.06 | 0.00 | 0.18 | 0.12 | 0.02 | 0.11 | 0.19 | 0.04 | 0.11 | 0.15 | 0.04 |
|  | footprint | 0.87 | 0.84 | 0.79 | 0.97 | 0.97 | 0.95 | 0.98 | 0.99 | 0.98 | 0.92 | 0.94 | 0.90 |
| GISTIC | G-score | 0.54 | 0.48 | 0.32 | 0.98 | 0.98 | 0.96 | **1.00** | **1.00** | **1.00** | 0.84 | 0.81 | 0.74 |
| KC-SMART | KC-score | 0.08 | 0.00 | 0.00 | 0.00 | 0.00 | 0.00 | 0.00 | 0.00 | 0.00 | 0.00 | 0.00 | 0.00 |
| CMDS | *w*=10 | 0.03 | 0.01 | 0.00 | 0.09 | 0.13 | 0.01 | 0.35 | 0.34 | 0.13 | 0.08 | 0.08 | 0.01 |
|  | *w*=20 | 0.03 | 0.01 | 0.00 | 0.12 | 0.17 | 0.04 | 0.28 | 0.29 | 0.12 | 0.16 | 0.13 | 0.03 |
|  | *w*=30 | 0.01 | 0.03 | 0.00 | 0.22 | 0.19 | 0.06 | 0.04 | 0.13 | 0.01 | 0.11 | 0.16 | 0.02 |
| SAIC | U-score | **0.92** | **0.87** | **0.83** | **1.00** | **0.99** | **0.99** | **1.00** | **1.00** | **1.00** | **0.97** | **0.96** | **0.94** |

1. *β_L_* = 2, *β_ω_* = 1.5, *σ*= (0.4, 0.6)

| Method | Statistic/  window | SCA 1 | | | SCA 2 | | | SCA 3 | | | SCA 4 | | |
| --- | --- | --- | --- | --- | --- | --- | --- | --- | --- | --- | --- | --- | --- |
|  |  | Start | End | Both | Start | End | Both | Start | End | Both | Start | End | Both |
| STAC | frequency | 0.07 | 0.07 | 0.00 | 0.28 | 0.29 | 0.11 | 0.45 | 0.29 | 0.21 | 0.05 | 0.06 | 0.00 |
|  | footprint | 0.40 | 0.45 | 0.20 | 0.76 | 0.81 | 0.63 | **0.95** | 0.90 | 0.89 | 0.65 | 0.66 | 0.59 |
| GISTIC | G-score | 0.27 | 0.26 | 0.11 | 0.82 | 0.87 | 0.72 | 0.92 | 0.92 | 0.85 | 0.61 | 0.63 | 0.51 |
| KC-SMART | KC-score | 0.11 | 0.05 | 0.00 | 0.01 | 0.00 | 0.00 | 0.00 | 0.00 | 0.00 | 0.01 | 0.00 | 0.00 |
| CMDS | *w*=10 | 0.03 | 0.05 | 0.00 | 0.21 | 0.18 | 0.04 | 0.29 | 0.39 | 0.11 | 0.15 | 0.10 | 0.03 |
|  | *w*=20 | 0.03 | 0.04 | 0.01 | 0.19 | 0.27 | 0.07 | 0.05 | 0.08 | 0.00 | 0.16 | 0.20 | 0.04 |
|  | *w*=30 | 0.01 | 0.04 | 0.00 | 0.13 | 0.11 | 0.02 | 0.00 | 0.00 | 0.00 | 0.07 | 0.11 | 0.01 |
| SAIC | U-score | **0.52** | **0.48** | **0.27** | **0.90** | **0.89** | **0.80** | 0.94 | **0.96** | **0.90** | **0.84** | **0.89** | **0.81** |

1. *β_L_* = 2, *β_ω_* = 1.5, *σ*= (0.6, 0.8)

| Method | Statistic/  window | SCA 1 | | | SCA 2 | | | SCA 3 | | | SCA 4 | | |
| --- | --- | --- | --- | --- | --- | --- | --- | --- | --- | --- | --- | --- | --- |
|  |  | Start | End | Both | Start | End | Both | Start | End | Both | Start | End | Both |
| STAC | frequency | 0.02 | 0.02 | 0.00 | 0.26 | 0.29 | 0.13 | 0.34 | 0.35 | 0.16 | 0.04 | 0.05 | 0.01 |
|  | footprint | 0.11 | 0.13 | 0.04 | 0.50 | 0.53 | 0.24 | **0.68** | **0.75** | **0.49** | 0.43 | 0.51 | 0.33 |
| GISTIC | G-score | 0.03 | 0.08 | 0.01 | **0.55** | **0.57** | **0.34** | 0.63 | 0.72 | 0.43 | 0.52 | 0.59 | 0.41 |
| KC-SMART | KC-score | 0.03 | 0.01 | 0.00 | 0.07 | 0.06 | 0.01 | 0.03 | 0.03 | 0.00 | 0.04 | 0.02 | 0.00 |
| CMDS | *w*=10 | 0.02 | 0.05 | 0.00 | 0.12 | 0.13 | 0.01 | 0.36 | 0.22 | 0.07 | 0.14 | 0.14 | 0.02 |
|  | *w*=20 | 0.02 | 0.06 | 0.00 | 0.12 | 0.13 | 0.02 | 0.10 | 0.09 | 0.01 | 0.15 | 0.12 | 0.06 |
|  | *w*=30 | 0.04 | 0.02 | 0.00 | 0.13 | 0.07 | 0.01 | 0.01 | 0.03 | 0.00 | 0.11 | 0.05 | 0.00 |
| SAIC | U-score | **0.22** | **0.31** | **0.13** | 0.52 | 0.50 | 0.28 | 0.65 | 0.72 | 0.45 | **0.79** | **0.74** | **0.64** |
