# Supplementary material for: Genome-wide identification of significant aberrations in cancer genome
Source: BMC Genomics. 2012 Jul 27;13:342. doi: 10.1186/1471-2164-13-342 (PMC3428679; doi:10.1186/1471-2164-13-342)
Supplement: Additional file 2 — Table S2 and Table S3. Details about the implicated SCAs and full list of genes covered by these SCAs, derived from the ovarian cancer data set. [file 1471-2164-13-342-S2.doc]

**Supplementary Table 2.** Details about the implicated SCAs and full list of genes covered by these SCAs, derived from genome-wide analysis of the ovarian cancer data set. (Start: Start point of SCAs. End: End point of SCAs.) (Based on hg18 human genome assembly)

| **Cyto-**  **band** | **Region** | | **P-value** | **Genes covered by the SCA regions** |
| --- | --- | --- | --- | --- |
|  | **Start** | **End** |  |  |
| ***Amplification*** | | | | |
| 1p31.1 | 77279618 | 77279715 | <0.001 | ST6GALNAC5 |
| 1q42.2 | 232375554 | 233956769 | 0.036 | SLC35F3,LOC440733,C1orf31,TARBP1,IRF2BP2,TOMM20,RBM34,ARID4B,GGPS1,TBCE,B3GALNT2,GNG4,LOC645645,LYST |
| 5p15.33 | 431413 | 3610671 | 0.048 | AHRR,LOC116349,EXOC3,SLC9A3,CEP72,TPPP,LOC653082,LOC643702,LOC643740,ZDHHC11,LOC653350,BRD9,TRIP13,NKD2,SLC12A7,SLC6A19,SLC6A18,**TERT**,CRR9,SLC6A3,AYTL2,LOC653102,LOC642535,LOC653378,LOC442128,MRPL36,NDUFS6,LOC389267,LOC644065,IRX4,IRX2,CEI,LOC285577 |
| 8p23.3 | 189547 | 1926964 | 0.027 | LOC644147,FBXO25,C8orf42,LOC389607,ERICH1,C8orf68,LOC401442,LOC644319,LOC644327,LOC644421,DLGAP2,CLN8,C8orf61,ARHGEF10,LOC644668,KBTBD11,LOC644711 |
| 8p23.2 | 3565440 | 6775890 | 0.046 | **CSMD1**,LOC392179,LOC392180,MCPH1,ANGPT2,AGPAT5,XKR5,DEFB1,LOC392181,LOC645265,DEFA6 |
| 8p22 | 14321795 | 19296766 | 0.024 | SGCZ,TUSC3,LOC137012,LOC646433,MSR1,LOC646440,MRPL49P2,LOC646444,FGF20,EFHA2,ZDHHC2,CNOT7,VPS37A,MTMR7,LOC646479,SLC7A2,PDGFRL,MTUS1,FGL1,PCM1,ASAH1,MRPS18CP3,NAT1,AACP,LOC392206,NAT2,LOC653754,PSD3,LOC442382,SH2D4A |
| 8p21.3 | 19299555 | 20330273 | 0.005 | ChGn,C8orf35,LPL,SLC18A1,ATP6V1B2,LZTS1 |
| 8q22.3 | 105150171 | 110602670 | 0.007 | RIMS2,TM7SF4,DPYS,LRP12,LOC644103,ZFPM2,LOC346887,OXR1,LOC643319,STARS,LOC644199,ANGPT1,RSPO2,LOC644233,EIF3S6,KIAA0103,TMEM74,TRHR,NUDCD1,ENY2,PKHD1L1,MAPK6PS5 |
| 8q24.21 | 129856608 | 138138546 | <0.001 | CCDC26,MLZE,FAM49B,DDEF1,ADCY8,KIAA0143,HHLA1,KCNQ3,LRRC6,TMEM71,PHF20L1,TG,SLA,WISP1,NDRG1,LOC392271,FAM10A6,ST3GAL1,ZNF406,SAS-ZFAT,LOC645809,LOC286094,KHDRBS3,LOC645921 |
| 8q24.23 | 138379880 | 144508547 | 0.011 | FLJ45872,C8ORFK32,COL22A1,KCNK9,NIBP,C8orf17,LOC644167,CHRAC1,LOC646107,EIF2C2,PTK2,DENND3,SLC45A4,GPR20,PTP4A3,FLJ43860,TSNARE1,BAI1,ARC,JRK,PSCA,LY6K,C8orf55,SLURP1,LYPD2,LYNX1,LY6D,GML,LOC646338,CYP11B1,CYP11B2,LY6E,C8orf31,LOC642276,LOC642295,HHCM,LY6H,LOC338328,ZFP41,GLI4,LOC642405,ZNF696,TOP1MT |
| 12p13.33 | 36594 | 4570482 | 0.007 | IQSEC3,SLC6A12,SLC6A13,JARID1A,MGC13183,B4GALNT3,NINJ2,WNK1,HSN2,RAD52,LOC642821,RAB6IP2,LOC653152,FBXL14,WNT5B,ADIPOR2,CACNA2D4,LOC644564,DCP1B,CACNA1C,LOC283440,LOC341511,FKBP4,MDS028,NRIP2,FOXM1,MGC13204,TULP3,TEAD4,LOC387825,TSPAN9,LOC643057,LOC643119,LOC643152,HRMT1L4,MGC4266,PARP11,HIN1L,LOC399988,LOC390281,**CCND2**,C12orf5,FGF23,FGF6,C12orf4,RAD51AP1,DYRK4 |
| 12p13.32 | 4589936 | 20323537 | <0.001 | DYRK4,AKAP3,NDUFA9,GALNT8,KCNA6,KCNA1,LOC390282,KCNA5,LOC387826,NTF3,TMEM16B,VWF,CD9,LOC653324,PLEKHG6,TNFRSF1A,SCNN1A,LTBR,LOC390283,LOC390284,TNFRSF7,TAPBPL,VAMP1,PKP2P1,MRPL51,CNAP1,GAPDH,HOM-TES-103,NOL1,CHD4,GPR92,ACRBP,ING4,ZNF384,DKFZp547D2210,COPS7A,MLF2,PTMS,LAG3,CD4,GPR162,LEPREL2,GNB3,CDCA3,USP5,TPI1,SPSB2,LOC283345,B7,ENO2,ATN1,GRCC10,PTPN6,PHB2,EMG1,C3F,LOC390285,C1S,LOC643676,LOC653342,C1R,C1RL,RBP5,CLSTN3,PEX5,LOC341392,CD163L1,LOC642333,CD163,LOC643739,LOC643746,APOBEC1,GDF3,DPPA3,CLEC4C,NANOG,SLC2A14,LOC643781,LOC642464,LOC643796,SLC2A3,LOC283320,FOXJ2,C3AR1,NECAP1,CLEC4A,LOC642559,ZNF705A,LOC643841,FAM90A1,LOC643856,LOC653113,LOC642616,LOC643864,LOC389634,LOC643882,LOC643889,OR7E140P,OR7E148P,OR7E149P,CLEC6A,CLEC4D,CLEC4E,AICDA,LOC399994,MFAP5,FAM80B,A2ML1,PHC1,M6PR,KLRG1,LOC643989,LOC643996,MGC40170,A2M,PZP,A2MP,PTMAP4,LOC642846,LOC644081,LOC440080,DDX12,LOC644125,KLRB1,LOC374443,LOC644138,LOC644143,CLEC2D,DCAL1,CD69,LOC644170,LOC644184,KLRF1,CLEC2B,CLEC2A,FLJ46363,CLEC12A,CLEC1B,UNQ5782,LOC644221,CLEC9A,CLEC1A,LOC390294,CLEC7A,OLR1,FLJ31166,GABARAPL1,KLRD1,LOC644252,KLRK1,KLRC4,KLRC3,KLRC2,KLRC1,LOC255308,KLRA1,FLJ10292,STYK1,CSDA,LOC644286,TAS2R7,TAS2R8,TAS2R9,TAS2R10,PRR4,LOC440082,PRH1,TAS2R12,TAS2R13,PRH2,TAS2R14,TAS2R15,TAS2R50,TAS2R49,TAS2R48,TAS2R44,TAS2R63P,TAS2R46,TAS2R64P,TAS2R43,TAS2R65P,PS5,T2R55,PRB3,PRB4,PRB2,PRB1,LOC653247,LOC440084,LOC644346,LOC644359,LOC644375,ETV6,BCL2L14,LOC643287,LRP6,MORF4LP4,MANSC1,LOH12CR1,DUSP16,LOC644467,CREBL2,GPR19,CDKN1B,DKFZP434F0318,DDX47,LOC440086,LOC387841,GPRC5A,GPRC5D,HEBP1,KIAA1467,GSG1,LOC644574,EMP1,FLJ33810,GRIN2B,LOC644693,ATF7IP,FLJ22662,GUCY2C,HIST4H4,H2AFJ,WBP11,MGC47869,LOC440087,ART4,MGP,FLJ32115,ARHGDIB,PDE6H,RERG,PTPRO,EPS8,MRPS7P2,STRAP,DERA,MGST1,LOC400011,LOC121520,LMO3,LOC387845,LOC644839,LOC644843,LOC390297,LOC390298,LOC644867,FLJ22655,PIK3C2G,PLCZ1,LOC643668,LOC643674,CAPZA3,LOC644897,RPL7P6,PLEKHA5,LOC644926,AEBP2,LOC400013,LOC644976,LOC644983 |
| 12p12.2 | 20352871 | 22327036 | 0.007 | PDE3A,SLCO1C1,SLCO1B3,SLCO1B1,SLCO1A2,IAPP,FLJ22028,RECQL,GOLT1B,MGC10946,GYS2,LDHB,KCNJ8,ABCC9,CMAS,ST8SIA1 |
| 12p12.1 | 24921458 | 26250197 | <0.001 | BCAT1,LOC441630,LOC645167,LOC645177,LOC645186,LRMP,CASC1,LOC144363,**KRAS**,FLJ36004,LOC645233,RASSF8,BHLHB3,SSPN |
| 12p12.1 | 26257574 | 27526862 | 0.004 | SSPN,ITPR2,C12orf11,FGFR1OP2,TM7SF3,SURB7,LOC440091,STK38L,**ARNTL2**,LOC645320,LOC341346 |
| 12p11.23 | 27548428 | 27971480 | 0.013 | PPFIBP1,REP15,MRPS35,KLHDC5 |
| 12q14.1 | 61982815 | 69568653 | 0.001 | DPY19L2,LOC390338,TMEM5,LOC341315,SRGAP1,FLJ32549,LOC115749,XPOT,LOC653581,TBK1,RASSF3,GNS,KIAA0984,WIF1,LEMD3,MSRB3,LOC645253,HMGA2,LOC645270,MGC14817,TMBIM4,IRAK3,RBMS1P,LOC390340,HELB,GRIP1,LOC645305,GGTA1P,CAND1,LOC645328,DYRK2,LOC341333,IFNG,IL26,IL22,MDM1,LOC160410,LOC387867,RAP1B,LOC645422,LOC246723,NUP107,SLC35E3,**MDM2**,CPM,CPSF6,LYZ,YEATS4,FRS2,CCT2,LRRC10,VMD2L3,MGC13168,LOC645495,RAB3IP,LOC645507,C12orf28,CNOT2,KCNMB4,PTPRB,PTPRR |
| 14q32.13 | 95778535 | 95778585 | 0.041 | BDKRB2 |
| 19q13.11 | 33591978 | 35333863 | 0.006 | LOC642570,LOC642608,UQCRFS1,LOC284395,LOC342865,POP4,PLEKHF1,C19orf12,**CCNE1**,LOC126170,C19orf2,TAF2GL |
| ***Deletion*** | | | | |
| 4q23 | 99571456 | 113520616 | 0.038 | RAP1GDS1,TSPAN5,LOC132556,EIF4E,TBCAP3,LOC644631,LOC644639,METAP1,ADH5,ADH4,PCNAP1,ADH6,ADH1A,ADH1B,ADH1C,LOC644666,ADH7,C4orf17,RG9MTD2,MTTP,LOC285556,DAPP1,MAP2K1IP1,DNAJB14,H2AFZ,LOC644721,DDIT4L,EMCN,PPP3CA,FLJ20021,BANK1,SLC39A8,NFKB1,MANBA,UBE2D3,LOC493856,LOC391404,LOC150159,LOC133308,LOC653330,DHRS6,CENPE,TACR3,CXXC4,LOC644864,LOC391679,LOC643675,FLJ20032,PPA2,LOC441032,LOC402182,ATP5EP1,FLJ20184,FLJ43963,LOC644892,PHF22,FLJ13273,NPNT,MGC16169,SCYE1,DKK2,RAC1P5,PAPSS1,LOC644968,MGC26963,CYP2U1,HADHSC,LEF1,LOC644980,LOC644985,LOC641518,LOC644993,LOC645003,LOC391681,FLJ37673,RPL34,DC2,AGXT2L1,COL25A1,LOC645078,LOC645087,SEC24B,LOC389217,FLJ20647,CASP6,PLA2G12A,IF,NOLA1,RRH,FLJ44691,LOC442114,EGF,ELOVL6,LOC132706,LOC132707,LOC645145,ENPEP,PITX2,LOC391686,LOC645193,LOC132719,LOC402184,FLJ39370,C4orf16,TIFA,LOC645237,ALPK1 |
| 6q14.1 | 77497537 | 77497656 | 0.049 |  |
| 8p23.3 | 189547 | 6891919 | <0.001 | LOC644147,FBXO25,C8orf42,LOC389607,ERICH1,C8orf68,LOC401442,LOC644319,LOC644327,LOC644421,DLGAP2,CLN8,C8orf61,ARHGEF10,LOC644668,KBTBD11,LOC644711,MYOM2,**CSMD1**,LOC392179,LOC392180,MCPH1,ANGPT2,AGPAT5,XKR5,DEFB1,LOC392181,LOC645265,DEFA6,DEFA4,DEFA8P,LOC645285,DEFA1,LOC645303,LOC653600,LOC645316,DEFA3,LOC645336 |
| 8p23.1 | 6898012 | 6966042 | 0.031 | DEFA5 |
| 8p23.1 | 6998485 | 6998485 | 0.005 |  |
| 8p23.1 | 8147895 | 8338822 | <0.001 | DKFZp761P0423 |
| 8p23.1 | 8355227 | 8897157 | 0.001 | CLDN23,MFHAS1,MRPS18CP2,LOC645960 |
| 8p23.1 | 8898659 | 9407969 | 0.001 | THEX1,RNU7P4,PPP1R3B,LOC645986 |
| 8p23.1 | 9410926 | 23649933 | <0.001 | TNKS,MSRA,LOC346702,UNQ9391,RP1L1,LOC203076,SOX7,PINX1,XKR6,C8orf15,C8orf16,LOC392193,MTMR9,AMAC1L2,TDH,C8orf13,BLK,GATA4,C8orf49,NEIL2,FDFT1,CTSB,OR7E158P,OR7E161P,DEFB137,DEFB136,DEFB134,LOC646244,LOC646253,OR7E160P,LOC646266,LOC440053,LOC392196,LOC392197,DUB3,FAM90A2P,LOC646287,LOC646290,LOC653726,FAM86B1,ZNF705CP,LOC653727,LOC646304,LOC389633,LOC646318,LOC646323,LOC653333,LOC646344,LOC653337,LOC646354,OR7E8P,OR7E15P,OR7E10P,LONRF1,FLJ36980,KIAA1456,DLC1,C8orf48,SGCZ,TUSC3,LOC137012,LOC646433,MSR1,LOC646440,MRPL49P2,LOC646444,FGF20,EFHA2,ZDHHC2,CNOT7,VPS37A,MTMR7,LOC646479,SLC7A2,**PDGFRL**,MTUS1,FGL1,PCM1,ASAH1,MRPS18CP3,NAT1,AACP,LOC392206,NAT2,LOC653754,PSD3,LOC442382,SH2D4A,ChGn,C8orf35,LPL,SLC18A1,ATP6V1B2,LZTS1,RNU3P2,LOC646608,GFRA2,LOC653765,OR6R2P,DOK2,XPO7,NPM2,FGF17,EPB49,RAI16,NUDT18,HR,C8orf20,LGI3,SFTPC,BMP1,PHYHIP,LOC646654,POLR3D,PIWIL2,SLC39A14,PPP3CC,SORBS3,PDLIM2,C8orf58,KIAA1967,BIN3,FLJ14107,EGR3,PEBP4,RHOBTB2,TNFRSF10B,TNFRSF10C,TNFRSF10D,TNFRSF10A,LOC389641,CHMP7,R3HCC1,LOXL2,ENTPD4,LOC646708,SLC25A37,LOC653778,LOC646721,NKX3-1,NKX2-6,LOC646731 |
| 8q24.23 | 138378921 | 138379138 | <0.001 |  |
| 9p24.2 | 3058621 | 24413220 | <0.001 | RFX3,LOC645849,GLIS3,LOC645856,SLC1A1,C9orf68,PPAPDC2,CDC37L1,AK3,LOC392282,RCL1,JAK2,LOC642611,IGHEP2,INSL6,INSL4,RLN2,LOC645930,RLN1,C9orf46,CD274,PDCD1LG2,KIAA1432,KIAA1815,LOC645952,MLANA,KIAA2026,LOC441385,RANBP6,C9orf26,LOC645969,TPD52L3,UHRF2,C9orf38,GLDC,SNRPEL1,JMJD2C,LOC158345,LOC392285,LOC646041,C9orf123,**PTPRD**,RPS26P3,RN7SLP2,LOC646087,LOC646101,LOC646105,LOC646111,LOC646114,LOC646133,LOC646138,TYRP1,C9orf150,LOC646153,TDPX2,MPDZ,LOC646181,FLJ41200,LOC646206,LOC646211,LOC347193,LOC138864,NFIB,ZDHHC21,CER1,FREM1,LDHAL4,LOC389705,PSIP1P,C9orf52,LOC286348,SNAPC3,PSIP1,FTHL12,LOC646305,C9orf93,LOC646371,BNC2,MGC24103,C9orf39,SH3GL2,LOC646428,ADAMTSL1,C9orf94,C9orf138,PSMC3P,RRAGA,FAM29A,ADFP,LOC253482,C9orf55,RPS6,ASAH3L,LOC392288,SLC24A2,LOC646505,SMNP,MLLT3,KIAA1797,PTPLAD2,LOC646525,IFNB1,IFNW1,IFNA21,LOC392289,IFNA4,IFNA7,IFNA10,G13P1,LOC392291,IFNA16,IFNA17,LOC392292,IFNA14,IFNAP22,IFNA5,KLHL9,IFNA6,IFNA13,IFNA2,IFNWP12,IFNA8,LOC646581,IFNA1,IFNWP19,IFNE1,LOC402359,MTAP,C9orf53,**CDKN2A,CDKN2B**,LOC646605,DMRTA1,FLJ35282,LOC646609,LOC646611,LOC402360,ELAVL2 |
| 9p21.3 | 24545849 | 27768662 | 0.001 | LOC646646,TUSC1,FLJ16323,C9orf82,PLAA,IFT74,LRRC19,TEK,C9orf14,C9orf11,MOBKL2B,IFNK,C9orf72,LOC392298 |
| 13q21.3 | 63177133 | 63204396 | 0.044 |  |
| 16p13.11 | 16170651 | 16170862 | 0.023 | ABCC6 |
| 16q23.3 | 81840807 | 81840912 | 0.013 | CDH13 |
| 17q22 | 51518046 | 51522852 | <0.001 |  |
| 18q11.2 | 24449765 | 24450128 | <0.001 |  |
| 18q22.1 | 64902464 | 76104900 | <0.001 | DOK6,CD226,RTTN,SOCS6,RPS2P6,GTSCR1,LOC643734,LOC643765,CBLN2,NETO1,LOC388481,LOC400655,FBXO15,C18orf55,CYB5,LOC644041,FAUP1,C18orf51,CNDP2,CNDP1,LOC400657,ZNF407,ZADH2,SDCCAG33,LOC284274,ZNF516,FLJ44313,FLJ44881,LOC644657,ZNF236,MBP,LOC642534,GALR1,LOC645144,LOC645321,SALL3,ATP9B,LOC653054,LOC653063,LOC653069,NFATC1,FLJ25715,CTDP1,LOC645411,KCNG2,PQLC1,LOC440498,TXNL4A,C18orf22,KIAA0863,PARD6G |

**Supplementary Table 3.** Details about the implicated SCAs and full list of genes covered by these SCAs, derived from chromosomal analysis of the ovarian cancer data set. (Start: Start point of SCAs. End: End point of SCAs.) (Based on hg18 human genome assembly)

| **Cyto-**  **band** | **Region** | | **P-value** | **Genes covered by the SCA regions** |
| --- | --- | --- | --- | --- |
|  | **Start** | **End** |  |  |
| ***Amplification*** | | | | |
| 1p31.1 | 77279618 | 77279715 | <0.001 | ST6GALNAC5 |
| 1q42.2 | 232375554 | 233956769 | <0.001 | SLC35F3,LOC440733,C1orf31,TARBP1,IRF2BP2,TOMM20,RBM34,ARID4B,GGPS1,TBCE,B3GALNT2,GNG4,LOC645645,LYST |
| 1q42.2 | 233993760 | 238785543 | <0.001 | LYST,NID1,LOC343508,TM7SF1,ERO1LB,EDARADD,ENO1P,LGALS8,LOC645721,HEATR1,ACTN2,LOC644343,MTR,LOC149448,LOC440737,LOC645745,RYR2,ZP4,LOC391179,LOC653674,CHRM3,LOC128136,LOC645884,FMN2,LOC266783,GREM2 |
| 1q43 | 238809528 | 242379385 | 0.006 | GREM2,LOC645916,LOC645939,RGS7,LOC388755,FH,KMO,OPN3,CHML,WDR64,LOC391181,EXO1,LOC645962,LOC441925,LOC645980,MAP1LC3C,LOC200149,PLD5,LOC645998,LOC646001,LOC391183,LOC646070,LOC284701,CEP170,SDCCAG8,AKT3,LOC339529,LOC646116,ZNF238,LOC440742,LOC646135 |
| 1q43 | 242384974 | 245756155 | 0.029 | C1orf100,LOC126826,ADSS,C1orf101,CYCSP5,C1orf121,FAM36A,HNRPU,EFCAB2,LOC653718,LOC646263,MGC35030,FLJ10157,SMYD3,TFB2M,C1orf71,SCCPDH,LOC149134,AHCTF1,ZNF695,ZNF670,ZNF669,FLJ45717,ZNF124,LOC343165,LOC441931,VN1R5,LOC646422,ZNF496,CIAS1,OR2B11,OR2W5,LOC644852 |
| 1q44 | 246038341 | 246106336 | 0.005 | OR5AT1,LOC646510,OR6R1P,OR11L1,TRIM58 |
| 2p21 | 45810049 | 55935059 | 0.004 | PRKCE,EPAS1,LOC388946,ATP6V1E2,RHOQ,PIGF,CRIPT,SOCS5,LOC388948,MCFD2,TTC7A,FLJ40172,CALM2,TACSTD1,MSH2,KCNK12,LOC644093,LOC285053,LOC440864,MSH6,FBXO11,LOC646907,HTLF,LOC129285,FLJ46838,SALF,SBLF,ALF,LHCGR,LOC646935,LOC646936,FSHR,LOC130728,NRXN1,LOC646948,CRYGGP1,LOC646958,LOC442015,ASB3,CHAC2,C2orf30,GPR75,PSME4,ACYP2,TSPYL6,FLJ40298,LOC442016,SPTBN1,RPL23AP13,LOC400954,RTN4,FLJ31438,RPS27A,MTIF2,LOC344405,KIAA1212,MGC15407,SMEK2,PNPT1 |
| 2q21.2 | 133649932 | 133650312 | 0.008 | FLJ34870 |
| 3q22.2 | 135058555 | 135058683 | <0.001 | RAB6B |
| 3q22.3 | 138325045 | 142858527 | <0.001 | NPM1P17,SOX14,LOC402143,CLDN18,DZIP1L,A4GNT,DBR1,ARMC8,TXNDC6,LOC653601,MRAS,LOC653764,FAM62C,CEP70,LOC646607,FAIM,LOC256374,PIK3CB,LOC646612,LOC646619,FOXL2,LOC401089,LOC389151,FLJ46210,LOC646641,BPESC1,MRPS22,COPB2,RBP2,ACTGP1,RBP1,NMNAT3,CLSTN2,TRIM42,LOC646685,SLC25A36,SPSB4,ACPL2,LOC645424,ZBTB38,LOC391584,RASA2 |
| 3q24 | 142908108 | 152811671 | <0.001 | LOC646730,RNF7,GRK7,ATP1B3,TFDP2,MGC40579,XRN1,ATR,PLS1,TRPC1,PCOLCE2,PAQR9,LOC646751,SR140,CHST2,PBXP1,SLC9A9,LOC645515,LOC257039,MGC33365,LOC646781,GM2AP,LOC389156,PLOD2,PLSCR4,LOC440981,PLSCR2,PLSCR1,PLSCR5,ZIC4,ZIC1,LOC440982,LOC646849,LOC344741,RPL38P1,AGTR1,CPB1,CPA3,UBQLN4P,GYG1,SMARCA3,HPS3,CP,LOC389160,TM4SF18,TM4SF1,LOC646886,TM4SF4,WWTR1,LOC440983,COMMD2,LOC389163,RNF13,PFN2,LOC646903,LOC653659,LOC391587,LOC646908,TSC22D2,SERP1,EIF2A,SELT,MGC39662,LOC391588,SIAH2,LOC646951,USH3A,MED12L,GPR171,P2RY14,GPR87,P2RY13,P2RY12,IGSF10 |
| 3q25.2 | 152846461 | 155667868 | 0.003 | AADACL2,LOC201651,AADAC,SUCNR1,LOC401093,MBNL1,LOC645843,P2RY1,LOC646992,RAP2B,LOC152118,SGEF,RH_II,GuBp2,DHX36,GPR149 |
| 3q25.31 | 156440778 | 156470603 | 0.02 | LOC647008 |
| 3q25.31 | 156487065 | 161061236 | <0.001 | PLCL3,C3orf33,SLC33A1,GMPS,LOC389168,CASRL1,LOC442505,MRE11B,KCNAB1,SSR3,LOC653801,TIPARP,LOC653803,LOC647033,FLJ16641,LOC152084,LOC647039,CCNL1,LOC391589,VEPH1,PTX3,LOC647046,LOC152078,SHOX2,RSRC1,MLF1,GFM1,LXN,RARRES1,MFSD1,GPR79,SCHIP1 |
| 3q26.1 | 161112778 | 161543267 | 0.004 | IL12A,LOC647071,LOC647076,LOC402145,LOC401097,IFT80 |
| 3q26.1 | 161543839 | 162475125 | 0.009 | IFT80,SMC4L1,TRIM59,LOC647084,KPNA4,KRT8L2,ARL14,PPM1L,B3GALT3,NMD3 |
| 3q26.1 | 162498283 | 162642079 | 0.006 | LOC646085,ADMP,LOC653814,LOC647099 |
| 3q26.1 | 162831610 | 165316352 | <0.001 | LOC647107,LOC647109, |
| 3q26.1 | 165316848 | 165316948 | <0.001 | NA |
| 3q26.1 | 165319181 | 165319181 | <0.001 | NA |
| 3q26.1 | 165319327 | 167940611 | 0.004 | LOC647122,SI,LOC647130,SLITRK3,BCHE,LOC389173 |
| 3q26.2 | 167966753 | 168701237 | 0.003 | LOC131055,FLJ23049,SERPINI2,WDR49 |
| 3q26.2 | 168847165 | 171033453 | <0.001 | WDR49,PDCD10,SERPINI1,LOC646168,GOLPH4,LOC389174,LOC253820,LOC647158,LOC93556,EVI1,MDS1,LOC646200,TERC,ARPM1,TRV1,MYNN,LRRC34,LOC344657 |
| 3q26.31 | 171093969 | 171260407 | 0.002 | LOC151825,SAMD7,TLOC1,GPR160 |
| 3q26.31 | 171344052 | 174170387 | <0.001 | PHC3,PRKCI,LOC647173,**SKIL**,CLDN11,FLJ37228,SLC7A14,LOC402149,LOC440990,EIF5A2,LOC442096,SLC2A2,TNIK,LOC646288,PLD1,FLJ23172,FNDC3B,LOC391592,GHSR,TNFSF10,LOC339858,LOC647199,AADACL1,ECT2,RNU4P4,SPATA16 |
| 3q26.31 | 174213388 | 176069211 | <0.001 | SPATA16,LOC647206,NLGN1,LOC402150,LOC647212,NAALADL2 |
| 3q26.32 | 176150140 | 177932812 | <0.001 | NAALADL2,LOC442097 |
| 3q26.32 | 177938928 | 178166128 | <0.001 | NA |
| 3q26.32 | 178185895 | 178198612 | <0.001 | NA |
| 3q26.32 | 178263193 | 184138552 | <0.001 | TBL1XR1,LOC339845,LOC401101,LOC646376,LOC647239,KCNMB2,WIG1,**PIK3CA**,KCNMB3,LOC647244,ZNF639,MFN1,GNB4,LOC647247,LOC442098,ACTL6A,MRPL47,NDUFB5,USP13,PEX5L,LOC647249,LOC131054,TTC14,CCDC39,LOC647255,LOC647253,LOC391595,FXR1,DNAJC19,SOX2,LOC402152,ATP11B |
| 3q27.1 | 184186423 | 186117711 | <0.001 | MCCC1,LAMP3,MCF2L2,B3GNT5,KLHL6,LOC647263,KLHL24,YEATS2,MAP6D1,PSARL,LOC391598,LOC647265,LOC391599,ABCC5,LOC389179,HTR3D,HTR3C,5-HT3c2,HTR3E,HSP90Ae,EIF2B5,LOC440991,DVL3,AP2M1,ABCF3,LOC90113,ALG3,MGC2408,ECE2,CAMK2N2,PSMD2,EIF4G1,C3orf40,CLCN2,POLR2H,THPO,CHRD,LOC391600,EPHB3,MAGEF1,KIAA0804 |
| 3q27.3 | 186162609 | 187713436 | <0.001 | KIAA0804,LOC285382,EHHADH,EIF2S2P2,LOC647274,MAP3K13,LOC647276,TMEM41A,LIPH,SENP2,IMP-2,LOC646600,SFRS10,LOC344887,ETV5,DGKG,LOC253573 |
| 3q27.3 | 187776514 | 187776642 | <0.001 | DNAJB11 |
| 3q27.3 | 187781075 | 197922333 | <0.001 | DNAJB11,AHSG,FETUB,HRG,LOC647280,KNG1,LOC280644,EIF4A2,RFC4,LOC647282,ADIPOQ,LOC440992,LOC647285,ST6GAL1,RPL39L,RTP1,MASP1,IFRG28,LOC647290,SST,RTP2,BCL6,FLJ42393,LPP,LOC647299,LOC647300,FAM79B,TP73L,LEPREL1,LOC391603,CLDN1,CLDN16,UNQ846,IL1RAP,LOC647309,OSTN,UTS2D,CCDC50,POP2,LOC647312,LOC647315,FGF12,LOC151963,LOC647319,HRASLS,ATP13A5,ATP13A4,OPA1,LOC647323,HES1,CPN2,LRRC15,GP5,ATP13A3,TMEM44,LSG1,FAM43A,LOC285303,C3orf21,CENTB2,PPP1R2,LOC653112,APOD,LOC642655,LOC440993,MUC20,MUC4,TNK2,LOC642706,SDHAL2,LOC642749,LOC642764,TFRC,FLJ25996,ZDHHC19,OSTalpha,PCYT1A,MGC33212,TM4SF19,UBXD7,RNF168,C3orf43,LOC642892,WDR53,FBXO45,LOC642923,LRRC33,C3orf34 |
| 3q29 | 197936635 | 198445298 | <0.001 | PIGX,PAK2,SENP5,NCBP2,PIGZ,LOC643013,LOC440995,MFI2,LOC391609,LOC643052,DLG1 |
| 3q29 | 198445583 | 199284641 | <0.001 | DLG1,BDH1,LOC643131,LOC220729,LOC643141,KIAA0226,FYTTD1,LRCH3,LOC642770,IQCG,RPL35A,LMLN,LOC653228,LOC348840 |
| 5p15.33 | 165712 | 399636 | <0.001 | KIAA1909,LOC389257,LOC133957,SDHA,PDCD6,AHRR |
| 5p15.33 | 431413 | 3610671 | <0.001 | AHRR,LOC116349,EXOC3,SLC9A3,CEP72,TPPP,LOC653082,LOC643702,LOC643740,ZDHHC11,LOC653350,BRD9,TRIP13,NKD2,SLC12A7,SLC6A19,SLC6A18,TERT,CRR9,SLC6A3,AYTL2,LOC653102,LOC642535,LOC653378,LOC442128,MRPL36,NDUFS6,LOC389267,LOC644065,IRX4,IRX2,CEI,LOC285577 |
| 5p15.33 | 3619830 | 3730182 | <0.001 | IRX1 |
| 5p15.33 | 3767522 | 7720153 | <0.001 | LOC340094,ADAMTS16,LOC442131,KIAA0947,LOC645267,FLJ33360,TRG20,FLJ25076,NSUN2,SRD5A1,POLS,LOC645463,LOC645451,LOC442132,ADCY2 |
| 5p15.31 | 7720353 | 7736544 | <0.001 | ADCY2 |
| 5p15.31 | 7764624 | 13589409 | <0.001 | ADCY2,LOC134121,MGC5297,MTRR,LOC645502,LOC645583,LOC645607,SEMA5A,TAS2R1,LOC134145,CCT5,LOC134147,MARCH6,ROPN1L,LOC645735,LOC345711,LOC645763,DAP,CTNND2,LOC645817 |
| 5p15.2 | 13591666 | 17797421 | <0.001 | LOC391738,DNAH5,TRIO,FAM105A,LOC645894,LOC391739,EEF1AL11,FAM105B,ANKH,LOC642954,LOC391741,FBXL7,LOC441061,ZNF622,FLJ20152,MYO10,LOC643003,FLJ34047,BASP1,LOC646012,FTHL10,LOC285697,LOC340096,LOC646032,LOC391742,LOC391745,LOC391746,LOC646066,LOC391747,LOC402199,LOC391749,LOC285563,LOC402200,LOC402201,LOC646103,LOC402203,LOC202201,LOC402205,LOC646126,LOC402207,LOC402208,LOC646152,LOC646165,LOC391761,LOC646188,LOC391763,LOC391764,LOC391765,LOC391766,LOC391767,LOC391768,LOC391769,LOC391770 |
| 5p15.1 | 17797885 | 20772541 | <0.001 | LOC646241,LOC646273,CDH18,LOC646280,LOC266786,LOC646296 |
| 5p14.3 | 20846434 | 30841081 | <0.001 | LOC646296,LOC646326,LOC646335,LOC646351,CDH12,LOC643288,LOC643300,PMCHL1,LOC391771,LOC646393,LOC646398,PRDM9,LOC439936,LOC503540,CDH10,LOC646435,LOC643379,MSNL1,LOC646504,CDH9,LOC643401,LOC646568,PGBD3P2,HPRTP2 |
| 5p13.3 | 30841110 | 31377931 | <0.001 | LOC391774,CDH6 |
| 5p13.3 | 31379276 | 34600592 | <0.001 | RNASEN,FLJ11193,PDZK3,GOLPH3,LOC202122,MTMR12,ZFR,LOC646616,SUB1,NPR3,FLJ14054,LOC340113,LOC643528,TARS,LOC646639,ADAMTS12,RLN3R1,SLC45A2,AMACR,C1QTNF3,LOC646650,LOC646652,LOC646653 |
| 5p13.2 | 34620378 | 35198519 | <0.001 | LOC401180,RAI14,FLJ25439,RAD1,BXDC2,DNAJA5,AGXT2,PRLR |
| 5p13.2 | 35201692 | 41710414 | <0.001 | PRLR,LOC646682,FLJ23577,IL7R,CAPSL,UGT3A1,LOC646695,UGT3A2,LMBRD2,SKP2,FLJ30596,FLJ25422,SLC1A3,LOC646719,NIPBL,LOC646723,LOC391777,FLJ13231,OFD1P1,LOC441068,NUP155,WDR70,LOC643787,LOC646739,GDNF,FLJ39155,LIFR,OSMR,RICTOR,FYB,C9,DAB2,LOC285634,PTGER4,OSRF,PRKAA1,RPL37,CARD6,C7,FLJ40243,C6,LOC646824,PLCXD3,TCP1L2 |
| 5p12 | 43332789 | 43620105 | 0.008 | HMGCS1,CCL28,FLJ21657,LOC646916,FLJ32363,PAIP1,LOC644116 |
| 5q11.1 | 50302214 | 50660886 | 0.038 | LOC133569 |
| 5q11.1 | 50672927 | 51658827 | <0.001 | LOC642366,ISL1 |
| 5q11.2 | 51678891 | 53815288 | <0.001 | PELO,ITGA1,ITGA2,MOCS2,LOC402214,FST,NDUFS4,LOC642374,ASSP9,ARL15,HSPB3 |
| 5q11.2 | 54069039 | 55851649 | <0.001 | LOC391783,LOC642646,ESM1,GZMK,GZMA,FLJ37927,LOC493869,LOC345643,UNG2,DHX29,SKIV2L2,PPAP2A,RNF138P1,FLJ90709,DDX4,LOC402216,IL31RA,IL6ST,FLJ11795,LOC345645,LOC642853,LOC441073,LOC441074 |
| 5q11.2 | 55853231 | 55939396 | 0.001 | LOC441074 |
| 5q32 | 145183591 | 148424379 | 0.002 | MGC21644,LOC643226,SH3RF2,PLAC8L1,LARS,POU4F3,TCERG1,GPR151,PPP2R2B,STK32A,DPYSL3,KIAA0555,SPINK1,SCGB3A2,MGC23985,SPINK5,SPINK5L2,SPINK6,LOC402232,SPINK5L3,ECG2,LOC643394,FBXO38,HTR4,ADRB2,SH3TC2,LOC255187 |
| 5q32 | 148424450 | 149890124 | <0.001 | LOC255187,ABLIM3,FLJ36748,GRPEL2,MGC3265,IL17B,CSNK1A1,FLJ41603,PPARGC1B,PDE6A,LOC644762,SLC26A2,TIGD6,RPS20P4,CSF1R,RPL7P,PDGFRB,CDX1,SLC6A7,CAMK2A,ARSI,TCOF1,CD74,RPS14,NDST1 |
| 12p13.33 | 36594 | 4570482 | <0.001 | IQSEC3,SLC6A12,SLC6A13,JARID1A,MGC13183,B4GALNT3,NINJ2,WNK1,HSN2,RAD52,LOC642821,RAB6IP2,LOC653152,FBXL14,WNT5B,ADIPOR2,CACNA2D4,LOC644564,DCP1B,CACNA1C,LOC283440,LOC341511,FKBP4,MDS028,NRIP2,FOXM1,MGC13204,TULP3,TEAD4,LOC387825,TSPAN9,LOC643057,LOC643119,LOC643152,HRMT1L4,MGC4266,PARP11,HIN1L,LOC399988,LOC390281,CCND2,C12orf5,FGF23,FGF6,C12orf4,RAD51AP1,DYRK4 |
| 12p13.32 | 4571424 | 4571564 | <0.001 | DYRK4 |
| 12p13.32 | 4589936 | 20323537 | <0.001 | DYRK4,AKAP3,NDUFA9,GALNT8,KCNA6,KCNA1,LOC390282,KCNA5,LOC387826,NTF3,TMEM16B,VWF,CD9,LOC653324,PLEKHG6,TNFRSF1A,SCNN1A,LTBR,LOC390283,LOC390284,TNFRSF7,TAPBPL,VAMP1,PKP2P1,MRPL51,CNAP1,GAPDH,HOM-TES-103,NOL1,CHD4,GPR92,ACRBP,ING4,ZNF384,DKFZp547D2210,COPS7A,MLF2,PTMS,LAG3,CD4,GPR162,LEPREL2,GNB3,CDCA3,USP5,TPI1,SPSB2,LOC283345,B7,ENO2,ATN1,GRCC10,PTPN6,PHB2,EMG1,C3F,LOC390285,C1S,LOC643676,LOC653342,C1R,C1RL,RBP5,CLSTN3,PEX5,LOC341392,CD163L1,LOC642333,CD163,LOC643739,LOC643746,APOBEC1,GDF3,DPPA3,CLEC4C,NANOG,SLC2A14,LOC643781,LOC642464,LOC643796,SLC2A3,LOC283320,FOXJ2,C3AR1,NECAP1,CLEC4A,LOC642559,ZNF705A,LOC643841,FAM90A1,LOC643856,LOC653113,LOC642616,LOC643864,LOC389634,LOC643882,LOC643889,OR7E140P,OR7E148P,OR7E149P,CLEC6A,CLEC4D,CLEC4E,AICDA,LOC399994,MFAP5,FAM80B,A2ML1,PHC1,M6PR,KLRG1,LOC643989,LOC643996,MGC40170,A2M,PZP,A2MP,PTMAP4,LOC642846,LOC644081,LOC440080,DDX12,LOC644125,KLRB1,LOC374443,LOC644138,LOC644143,CLEC2D,DCAL1,CD69,LOC644170,LOC644184,KLRF1,CLEC2B,CLEC2A,FLJ46363,CLEC12A,CLEC1B,UNQ5782,LOC644221,CLEC9A,CLEC1A,LOC390294,CLEC7A,OLR1,FLJ31166,GABARAPL1,KLRD1,LOC644252,KLRK1,KLRC4,KLRC3,KLRC2,KLRC1,LOC255308,KLRA1,FLJ10292,STYK1,CSDA,LOC644286,TAS2R7,TAS2R8,TAS2R9,TAS2R10,PRR4,LOC440082,PRH1,TAS2R12,TAS2R13,PRH2,TAS2R14,TAS2R15,TAS2R50,TAS2R49,TAS2R48,TAS2R44,TAS2R63P,TAS2R46,TAS2R64P,TAS2R43,TAS2R65P,PS5,T2R55,PRB3,PRB4,PRB2,PRB1,LOC653247,LOC440084,LOC644346,LOC644359,LOC644375,ETV6,BCL2L14,LOC643287,LRP6,MORF4LP4,MANSC1,LOH12CR1,DUSP16,LOC644467,CREBL2,GPR19,CDKN1B,DKFZP434F0318,DDX47,LOC440086,LOC387841,GPRC5A,GPRC5D,HEBP1,KIAA1467,GSG1,LOC644574,EMP1,FLJ33810,GRIN2B,LOC644693,ATF7IP,FLJ22662,GUCY2C,HIST4H4,H2AFJ,WBP11,MGC47869,LOC440087,ART4,MGP,FLJ32115,ARHGDIB,PDE6H,RERG,PTPRO,EPS8,MRPS7P2,STRAP,DERA,MGST1,LOC400011,LOC121520,LMO3,LOC387845,LOC644839,LOC644843,LOC390297,LOC390298,LOC644867,FLJ22655,PIK3C2G,PLCZ1,LOC643668,LOC643674,CAPZA3,LOC644897,RPL7P6,PLEKHA5,LOC644926,AEBP2,LOC400013,LOC644976,LOC644983 |
| 12p12.2 | 20352871 | 22327036 | <0.001 | PDE3A,SLCO1C1,SLCO1B3,SLCO1B1,SLCO1A2,IAPP,FLJ22028,RECQL,GOLT1B,MGC10946,GYS2,LDHB,KCNJ8,ABCC9,CMAS,ST8SIA1 |
| 12p12.1 | 22366156 | 23025546 | <0.001 | ST8SIA1,KIAA0528,ETNK1,LOC645102 |
| 12p12.1 | 23026858 | 23140376 | <0.001 | NA |
| 12p12.1 | 23144508 | 23164381 | <0.001 | LOC645117 |
| 12p12.1 | 23165048 | 24808118 | <0.001 | SOX5,FLJ32894 |
| 12p12.1 | 24819025 | 24821806 | <0.001 | NA |
| 12p12.1 | 24836266 | 24872878 | <0.001 | BCAT1 |
| 12p12.1 | 24876824 | 24892284 | <0.001 | BCAT1 |
| 12p12.1 | 24921458 | 26250197 | <0.001 | BCAT1,LOC441630,LOC645167,LOC645177,LOC645186,LRMP,CASC1,LOC144363,KRAS,FLJ36004,LOC645233,RASSF8,BHLHB3,SSPN |
| 12p12.1 | 26257574 | 27526862 | <0.001 | SSPN,ITPR2,C12orf11,FGFR1OP2,TM7SF3,SURB7,LOC440091,STK38L,ARNTL2,LOC645320,LOC341346 |
| 12p11.23 | 27539678 | 27541560 | <0.001 | LOC341346 |
| 12p11.23 | 27548428 | 27971480 | <0.001 | PPFIBP1,REP15,MRPS35,KLHDC5 |
| 12p11.22 | 27977793 | 28072500 | <0.001 | PTHLH |
| 12p11.22 | 28086392 | 28091522 | <0.001 | NA |
| 12p11.22 | 28091579 | 29000222 | <0.001 | LOC440092,DKFZP779L1558 |
| 12p11.22 | 29018271 | 29936009 | <0.001 | MLSTD1,PTX1,LOC645410,OVCH1,LOC653626,TMTC1 |
| 12p11.22 | 29982038 | 31241617 | <0.001 | IPO8,C1QDC1,LOC645485,LOC400019,LOC390299,TSPAN11,DDX11,OVOS2 |
| 12p11.21 | 31243957 | 31833373 | <0.001 | OVOS2,LOC441632,FAM60A,LOC645593,FLJ13224,MGC24039,MRPL30P2,LOC341356,LOC645619,MGC50559,LOC196394,LOC645636,LOC144383 |
| 12p11.21 | 31911142 | 33305740 | <0.001 | LOC645665,FLJ10652,BICD1,**FGD4**,DNM1L,YARS2,PKP2,LOC283343 |
| 12p11.1 | 33318968 | 33403184 | <0.001 | NA |
| 12p11.1 | 33429446 | 34198126 | <0.001 | SYT10,LOC390301,ALG10 |
| 12q13.2 | 55341558 | 58894656 | 0.028 | PTGES3,NACA,PRIM1,HSD17B6,SDR-O,LOC644764,RDH16,LOC390332,ADMR,ZBTB39,TAC3,MYO1A,KIAA0286,NAB2,STAT6,LRP1,NXPH4,SHMT2,LOC56901,STAC3,KIAA1002,INHBC,INHBE,GLI1,ARHGAP9,MARS,DDIT3,MBD6,DCTN2,KIF5A,PIP5K2C,DTX3,GEFT,SLC26A10,B4GALNT1,LOC441641,OS9,CENTG1,TSPAN31,**CDK4**,MARCH9,CYP27B1,METTL1,DKFZP586D0919,TSFM,AVIL,CTDSP2,XRCC6BP1,LOC338805,LOC644908,LRIG3,LOC644915,SLC16A7,LOC390335 |
| 12q14.1 | 58902277 | 58902745 | 0.032 | NA |
| 12q14.1 | 58931384 | 61568609 | 0.014 | LOC644957,PGBD3P1,FAM19A2,LOC644998,LOC645000,USP15,KIAA1040,FLJ25590,PPM1H |
| 12q14.1 | 61578624 | 61895738 | <0.001 | PPM1H,LOC645085,RPL14L,LOC645100,LOC121498,AVPR1A |
| 12q14.1 | 61896783 | 61896809 | <0.001 | NA |
| 12q14.1 | 61982815 | 69568653 | <0.001 | DPY19L2,LOC390338,TMEM5,LOC341315,SRGAP1,FLJ32549,LOC115749,XPOT,LOC653581,TBK1,RASSF3,GNS,KIAA0984,WIF1,LEMD3,MSRB3,LOC645253,HMGA2,LOC645270,MGC14817,TMBIM4,IRAK3,RBMS1P,LOC390340,HELB,GRIP1,LOC645305,GGTA1P,CAND1,LOC645328,DYRK2,LOC341333,IFNG,IL26,IL22,MDM1,LOC160410,LOC387867,RAP1B,LOC645422,LOC246723,NUP107,SLC35E3,MDM2,CPM,CPSF6,LYZ,YEATS4,FRS2,CCT2,LRRC10,VMD2L3,MGC13168,LOC645495,RAB3IP,LOC645507,C12orf28,CNOT2,KCNMB4,PTPRB,PTPRR |
| 12q15 | 69587543 | 70029255 | <0.001 | PTPRR,TSPAN8 |
| 12q15 | 70100887 | 71395453 | <0.001 | LGR5,PSRC2,THAP2,TMEM19,RAB21,TBC1D15,TPH2,LOC283392,TRHDE |
| 14q32.13 | 95778535 | 95778585 | <0.001 | BDKRB2 |
| 15q11.1 | 19827281 | 19840684 | 0.015 | OR4Q1P |
| 15q26.1 | 91980364 | 91980436 | <0.001 | NA |
| 15q26.2 | 94728761 | 100210760 | <0.001 | LOC644279,LOC388181,SPATA8,ARRDC4,FLJ39743,IGF1R,MGC18216,LOC145814,DMN,TTC23,LRRC28,LOC643430,LOC644752,MEF2A,LYSMD4,DKFZp434I1020,LOC644800,LOC400464,ADAMTS17,FLJ42289,LASS3,PRKXP1,LINS1,ASB7,LOC440313,ALDH1A3,LOC644916,FLJ27465,LRRK1,CHSY1,SELS,SNRPA1,PCSK6,TM2D3,TARSL2,LOC645032,LOC441734,LOC440315,LOC441735,LOC441736,OR4F6,OR4F15,OR4F14P,OR4F13P |
| 17q21.31 | 41569489 | 41630337 | <0.001 | KIAA1267,LOC644246 |
| 17q21.31 | 41644356 | 41704075 | <0.001 | LOC644253,LOC644256,LOC644264 |
| 17q21.31 | 41708649 | 41708649 | 0.001 | LOC644264 |
| 17q22 | 53976358 | 63643742 | <0.001 | C17orf47,TEX14,LOC645545,RAD51C,PPM1E,TRIM37,FAM33A,PRR11,C17orf71,GDPD1,YPEL2,LOC645563,DHX40,CLTC,BIT1,TMEM49,TUBD1,RPS6KB1,LOC51136,LOC653645,TBC1D3P1,LOC645603,ABC1,LOC645638,LOC653653,LOC441797,CA4,USP32,C17orf64,LOC645684,LOC645688,APPBP2,PPM1D,BCAS3,LOC645722,TBX2,LOC388407,TBX4,NACAL,BRIP1,KIAA1287,THRAP1,LOC284167,LOC645804,TBC1D3P2,LOC645822,FLJ25818,FLJ12760,TLK2,MRC2,RNF190,TANC2,CYB561,LOC645892,LOC342541,ACE,LOC653684,KCNH6,WDR68,CCDC44,LOC390806,MAP3K3,MGC10986,LOC440455,LYK5,CCDC47,DDX42,FTSJ3,PSMC5,SMARCD2,CSH2,GH2,CSH1,CSHL1,GH1,CD79B,SCN4A,LOC645966,ICAM2,ERN1,TEX2,LOC645993,PECAM1,C17orf60,POLG2,DDX5,CCDC45,SMURF2,MGC40489,LOC645625,LOC646026,LOC646014,LOC646022,LOC440456,LOC646038,FLJ34306,LOC646043,LOC646047,LOC440459,FLJ32065,GNA13,RGS9,AXIN2,CCDC46,LOC280637,APOH,PRKCA,LOC645710,CACNG5,CACNG4,CACNG1,HELZ,LOC646175,LOC646178,LOC441799,PSMD12,PITPNC1,NOL11,FALZ,C17orf58,KPNA2,SH3GLP3 |
| 17q24.1 | 63666271 | 65892761 | 0.001 | SH3GLP3,LOC440461,AMZ2,SLC16A6,KIAA1001,WIPI1,PRKAR1A,FAM20A,ABCA8,ABCA9,ABCA6,ABCA10,ABCA5,LOC642415,MAP2K6,LOC653093,LOC642494,KCNJ16,KCNJ2,LOC401887 |
| 17q24.2 | 66236699 | 68389374 | <0.001 | LOC124685,SOX9,SLC39A11 |
| 17q24.3 | 68404257 | 74433873 | <0.001 | SLC39A11,LOC390811,SSTR2,COG1,FAM104A,HLC-8,LOC642843,CDC42EP4,SDK2,C17orf54,RPL38,TTYH2,DNAI2,FLJ37300,LOC388419,GPR142,GPRC5C,CD300A,CD300LB,CD300C,LOC441800,FLJ31882,LOC643105,CD300LE,RAB37,CD300LF,SLC9A3R1,NAT9,TMEM104,GRIN2C,FDXR,FADS6,USH1G,OTOP2,OTOP3,C17orf28,CDR2L,ICT1,LOC643154,ATP5H,KCTD2,LOC643177,SLC16A5,ARMC7,NT5C,HN1,SUMO2,NUP85,GGA3,MRPS7,MIF4GD,SLC25A19,GRB2,LOC653241,KIAA0195,CASKIN2,TSEN54,LLGL2,LOC643248,MYO15B,RECQL5,LOC643008,SAP30BP,ITGB4,GALK1,H3F3B,LOC643260,ZC3H5,UNC13D,WBP2,TRIM47,TRIM65,MRPL38,FBF1,LOC643082,ACOX1,CDK3,EVPL,SRP68,GALR2,LGICZ,EXOC7,LOC643278,FOXJ1,RNF157,LOC643159,FAM100B,QRICH2,PRPSAP1,SPHK1,UBE2O,AANAT,RHBDL6,CYGB,ST6GALNAC2,ST6GALNAC1,MXRA7,PTDSR,LOC124512,SFRS2,ET,MGAT5B,SEC14L1,CYCSP40,SEPT9,LOC643283,FLJ45079,TNRC6C,TMC6,TMC8,EIF5AP2,SYNGR2,TK1,AFMID,BIRC5,EPR1,LOC283999,THA1P,SOCS3,DNAH17,DNAHL1,PSCD1,USP36,LOC643678,TIMP2 |
| 17q25.1 | 74442637 | 74749078 | 0.006 | LGALS3BP,CANT1,C1QTNF1,FLJ21865,LOC146713 |
| 17q25.1 | 74774728 | 77814474 | <0.001 | LOC146713,LOC643798,ENPP7,CBX2,CBX8,CBX4,TBC1D16,CCDC40,GAA,DDX48,CARD14,SGSH,SLC26A11,KIAA1618,C17orf27,FLJ35220,LOC643912,NPTX1,raptor,LOC653386,FLJ46026,CHMP6,LOC644292,FLJ90757,BAIAP2,AATK,FLJ44861,AZI1,C17orf56,LOC284184,MGC15523,C17orf55,TMEM105,LOC643982,BAHCC1,LOC644012,ACTG1,FSCN2,C17orf70,NPL4,TSPAN10,PDE6G,MGC16597,LOC339229,ARL16,HGS,MRPL12,SLC25A10,DYSFIP1,P4HB,ARHGDIA,THOC4,ANAPC11,NPB,PCYT2,SIRT7,MAFG,PYCR1,LOC255275,NOTUM,ASPSCR1,STRA13,LRRC45,RAC3,DCXR,RFNG,GPS1,DUS1L,FASN,FLJ23754,CCDC57,SLC16A3,CSNK1D |
| 22q11.21 | 19241756 | 20258641 | 0.018 | PCQAP,LOC645280,SLC9A3P2,LOC150207,LOC645289,POM121L4P,DKFZp434N035,LOC653603,PIK4CA,SERPIND1,SNAP29,CRKL,LOC400890,AIFL,LZTR1,THAP7,MGC16703,LOC645341,P2RXL1,SLC7A4,LOC653608,LOC400891,LOC645348,FLJ42953,LOC376818,LOC284861,LOC653257,LOC645367,LOC645376,LOC643318,LOC653617,LOC653264,LOC653270,LOC645390,LOC388853,LOC440804,HIC2,LOC645426,LOC220686,LOC375133,LOC150221,UBE2L3 |
| 17q24.3 | 68404257 | 74433873 | <0.001 | SLC39A11,LOC390811,SSTR2,COG1,FAM104A,HLC-8,LOC642843,CDC42EP4,SDK2,C17orf54,RPL38,TTYH2,DNAI2,FLJ37300,LOC388419,GPR142,GPRC5C,CD300A,CD300LB,CD300C,LOC441800,FLJ31882,LOC643105,CD300LE,RAB37,CD300LF,SLC9A3R1,NAT9,TMEM104,GRIN2C,FDXR,FADS6,USH1G,OTOP2,OTOP3,C17orf28,CDR2L,ICT1,LOC643154,ATP5H,KCTD2,LOC643177,SLC16A5,ARMC7,NT5C,HN1,SUMO2,NUP85,GGA3,MRPS7,MIF4GD,SLC25A19,GRB2,LOC653241,KIAA0195,CASKIN2,TSEN54,LLGL2,LOC643248,MYO15B,RECQL5,LOC643008,SAP30BP,ITGB4,GALK1,H3F3B,LOC643260,ZC3H5,UNC13D,WBP2,TRIM47,TRIM65,MRPL38,FBF1,LOC643082,ACOX1,CDK3,EVPL,SRP68,GALR2,LGICZ,EXOC7,LOC643278,FOXJ1,RNF157,LOC643159,FAM100B,QRICH2,PRPSAP1,SPHK1,UBE2O,AANAT,RHBDL6,CYGB,ST6GALNAC2,ST6GALNAC1,MXRA7,PTDSR,LOC124512,SFRS2,ET,MGAT5B,SEC14L1,CYCSP40,SEPT9,LOC643283,FLJ45079,TNRC6C,TMC6,TMC8,EIF5AP2,SYNGR2,TK1,AFMID,BIRC5,EPR1,LOC283999,THA1P,SOCS3,DNAH17,DNAHL1,PSCD1,USP36,LOC643678,TIMP2 |
| 17q25.1 | 74442637 | 74749078 | 0.006 | LGALS3BP,CANT1,C1QTNF1,FLJ21865,LOC146713 |
| 17q25.1 | 74774728 | 77814474 | <0.001 | LOC146713,LOC643798,ENPP7,CBX2,CBX8,CBX4,TBC1D16,CCDC40,GAA,DDX48,CARD14,SGSH,SLC26A11,KIAA1618,C17orf27,FLJ35220,LOC643912,NPTX1,raptor,LOC653386,FLJ46026,CHMP6,LOC644292,FLJ90757,BAIAP2,AATK,FLJ44861,AZI1,C17orf56,LOC284184,MGC15523,C17orf55,TMEM105,LOC643982,BAHCC1,LOC644012,ACTG1,FSCN2,C17orf70,NPL4,TSPAN10,PDE6G,MGC16597,LOC339229,ARL16,HGS,MRPL12,SLC25A10,DYSFIP1,P4HB,ARHGDIA,THOC4,ANAPC11,NPB,PCYT2,SIRT7,MAFG,PYCR1,LOC255275,NOTUM,ASPSCR1,STRA13,LRRC45,RAC3,DCXR,RFNG,GPS1,DUS1L,FASN,FLJ23754,CCDC57,SLC16A3,CSNK1D |
| 22q11.21 | 19241756 | 20258641 | 0.018 | PCQAP,LOC645280,SLC9A3P2,LOC150207,LOC645289,POM121L4P,DKFZp434N035,LOC653603,PIK4CA,SERPIND1,SNAP29,CRKL,LOC400890,AIFL,LZTR1,THAP7,MGC16703,LOC645341,P2RXL1,SLC7A4,LOC653608,LOC400891,LOC645348,FLJ42953,LOC376818,LOC284861,LOC653257,LOC645367,LOC645376,LOC643318,LOC653617,LOC653264,LOC653270,LOC645390,LOC388853,LOC440804,HIC2,LOC645426,LOC220686,LOC375133,LOC150221,UBE2L3 |
| ***Deletion*** | | | | |
| 2q37.3 | 240961788 | 240961802 | 0.014 | NA |
| 2q37.3 | 242567344 | 242663303 | 0.002 | FLJ40712,FLJ38379,FLJ41327 |
| 3p21.2 | 51012298 | 57330377 | 0.002 | DOCK3,ARMET,RBM15B,VPRBP,SRISNF2L,TEX264,GRM2,LOC440956,IQCF3,LOC645389,IQCF2,LOC389124,IQCF1,RNU3IP2,PARP3,GPR62,PCBP4,LOC653767,ABHD14B,ABHD14A,ACY1,RPL29,DUSP7,WDR51A,ALDOAP1,ALAS1,TLR9,PTK9L,PPM1M,TMEM113,GLYCTK,DNAH1,PPP2R5CP,BAP1,PHF7,SEMA3G,TNNC1,NISCH,STAB1,FLJ12442,LOC440957,PB1,GNL3,GLT8D1,SPCS1,NEK4,ITIH1,ITIH3,ITIH4,MUSTN1,TMEM110,SFMBT1,LOC401068,LOC553148,RFT1,PRKCD,TKT,DCP1A,LOC391539,CACNA1D,CHDH,IL17RB,ACTR8,SELK,CABYRP,CACNA2D3,LRTM1,WNT5A,CAST1,LOC285331,RAP140,ARHGEF3,SPATA12,IL17RD,HESX1,APPL,ASB14,DNHD2 |
| 3p14.3 | 57369806 | 60041684 | 0.001 | FLJ44290,FLJ34969,SLMAP,LOC344797,FLNB,DNASE1L3,ABHD6,RPP14,PXK,PDHB,KCTD6,ACOX2,TU3A,FAM3D,FLJ42117,LOC339902,FHIT,NPCDR1 |
| 3p14.2 | 60048193 | 60202432 | <0.001 | FHIT |
| 3p14.2 | 60225104 | 63152598 | 0.006 | FHIT,LOC131691,LOC391540,LOC391541,PTPRG,ID2B,C3orf14,ZNF312,CADPS,LOC389127,LOC132205 |
| 3q28 | 190577002 | 190580641 | 0.032 | NA |
| 3q28 | 191244558 | 191244743 | 0.032 | LEPREL1 |
| 3q28 | 191358612 | 191358892 | 0.003 | NA |
| 3q29 | 192689524 | 199284641 | 0.042 | LOC647312,LOC647315,FGF12,LOC151963,LOC647319,HRASLS,ATP13A5,ATP13A4,OPA1,LOC647323,HES1,CPN2,LRRC15,GP5,ATP13A3,TMEM44,LSG1,FAM43A,LOC285303,C3orf21,CENTB2,PPP1R2,LOC653112,APOD,LOC642655,LOC440993,MUC20,MUC4,TNK2,LOC642706,SDHAL2,LOC642749,LOC642764,TFRC,FLJ25996,ZDHHC19,OSTalpha,PCYT1A,MGC33212,TM4SF19,UBXD7,RNF168,C3orf43,LOC642892,WDR53,FBXO45,LOC642923,LRRC33,C3orf34,PIGX,PAK2,SENP5,NCBP2,PIGZ,LOC643013,LOC440995,MFI2,LOC391609,LOC643052,DLG1,BDH1,LOC643131,LOC220729,LOC643141,KIAA0226,FYTTD1,LRCH3,LOC642770,IQCG,RPL35A,LMLN,LOC653228,LOC348840 |
| 6q14.1 | 77497537 | 77497656 | 0.001 | NA |
| 8p23.3 | 189547 | 2509684 | <0.001 | LOC644147,FBXO25,C8orf42,LOC389607,ERICH1,C8orf68,LOC401442,LOC644319,LOC644327,LOC644421,DLGAP2,CLN8,C8orf61,ARHGEF10,LOC644668,KBTBD11,LOC644711,MYOM2 |
| 8p23.2 | 2536762 | 4782440 | <0.001 | CSMD1 |
| 8p23.2 | 4807585 | 6891919 | <0.001 | CSMD1,LOC392179,LOC392180,MCPH1,ANGPT2,AGPAT5,XKR5,DEFB1,LOC392181,LOC645265,DEFA6,DEFA4,DEFA8P,LOC645285,DEFA1,LOC645303,LOC653600,LOC645316,DEFA3,LOC645336 |
| 8p23.1 | 6898012 | 6966042 | <0.001 | DEFA5 |
| 8p23.1 | 6998485 | 6998485 | <0.001 | NA |
| 8p23.1 | 8147895 | 8338822 | <0.001 | DKFZp761P0423 |
| 8p23.1 | 8355227 | 8897157 | <0.001 | CLDN23,MFHAS1,MRPS18CP2,LOC645960 |
| 8p23.1 | 8898659 | 8898840 | <0.001 | THEX1 |
| 8p23.1 | 8912382 | 9407969 | <0.001 | THEX1,RNU7P4,PPP1R3B,LOC645986 |
| 8p23.1 | 9410926 | 12520366 | <0.001 | TNKS,MSRA,LOC346702,UNQ9391,RP1L1,LOC203076,SOX7,PINX1,XKR6,C8orf15,C8orf16,LOC392193,MTMR9,AMAC1L2,TDH,C8orf13,BLK,GATA4,C8orf49,NEIL2,FDFT1,CTSB,OR7E158P,OR7E161P,DEFB137,DEFB136,DEFB134,LOC646244,LOC646253,OR7E160P,LOC646266,LOC440053,LOC392196,LOC392197,DUB3,FAM90A2P,LOC646287,LOC646290,LOC653726,FAM86B1,ZNF705CP,LOC653727,LOC646304,LOC389633,LOC646318,LOC646323,LOC653333,LOC646344,LOC653337 |
| 8p23.1 | 12537556 | 23649933 | <0.001 | LOC653337,LOC646354,OR7E8P,OR7E15P,OR7E10P,LONRF1,FLJ36980,KIAA1456,DLC1,C8orf48,SGCZ,TUSC3,LOC137012,LOC646433,MSR1,LOC646440,MRPL49P2,LOC646444,FGF20,EFHA2,ZDHHC2,CNOT7,VPS37A,MTMR7,LOC646479,SLC7A2,PDGFRL,MTUS1,FGL1,PCM1,ASAH1,MRPS18CP3,NAT1,AACP,LOC392206,NAT2,LOC653754,PSD3,LOC442382,SH2D4A,ChGn,C8orf35,LPL,SLC18A1,ATP6V1B2,LZTS1,RNU3P2,LOC646608,GFRA2,LOC653765,OR6R2P,DOK2,XPO7,NPM2,FGF17,EPB49,RAI16,NUDT18,HR,C8orf20,LGI3,SFTPC,BMP1,PHYHIP,LOC646654,POLR3D,PIWIL2,SLC39A14,PPP3CC,SORBS3,PDLIM2,C8orf58,KIAA1967,BIN3,FLJ14107,EGR3,PEBP4,RHOBTB2,TNFRSF10B,TNFRSF10C,TNFRSF10D,TNFRSF10A,LOC389641,CHMP7,R3HCC1,LOXL2,ENTPD4,LOC646708,SLC25A37,LOC653778,LOC646721,NKX3-1,NKX2-6,LOC646731 |
| 8p21.2 | 23658974 | 28711850 | <0.001 | STC1,LOC646740,ADAM28,ADAMDEC1,ADAM7,NEF3,NEFL,DOCK5,GNRH1,KCTD9,CDCA2,EBF2,PPP2R2A,LOC157489,BNIP3L,LOC440258,PNMA2,LOC338097,DPYSL2,ADRA1A,LOC646818,STMN4,TRIM35,PTK2B,CHRNA2,EPHX2,GULOP,CLU,SCARA3,LOC646843,CCDC25,ESCO2,PBK,SCARA5,HMFN0672,ELP3,LOC389644,PNOC,ZNF395,FBXO16,FZD3,LOC653787,EXTL3,RC74 |
| 8p21.1 | 28715674 | 30303091 | <0.001 | RC74,FLJ21616,KIF13B,DUSP4,LOC646909,MAP2K1P1,TMEM66,LEPROTL1,DCTN6,LOC392209,LOC642319 |
| 8p12 | 30303295 | 33695814 | <0.001 | LOC92755,RBPMS,LOC642356,GTF2E2,GSR,LOC642388,UBXD6,PPP2CB,TEX15,LOC441344,PURG,WRN,LOC642513,LOC653104,NRG1,FUT10,RBM13,C8orf41,RNF122,DUSP26,LOC642685 |
| 8p12 | 33703169 | 33903256 | <0.001 | LOC388460 |
| 8p12 | 33927167 | 35286320 | <0.001 | CYCSP3,LOC137107 |
| 8p12 | 35315959 | 36339821 | <0.001 | LOC653122,UNC5D |
| 8p12 | 36355124 | 37001574 | <0.001 | LOC642855,MRPS7P1,FKSG2 |
| 8p11.23 | 37017410 | 37017442 | <0.001 | NA |
| 8p11.23 | 37018284 | 37075555 | <0.001 | LOC642879 |
| 8p11.23 | 37105077 | 37112027 | <0.001 | NA |
| 8p11.23 | 37113823 | 41820475 | <0.001 | LOC642950,ZNF703,SPFH2,PROSC,GPR124,BRF2,RAB11FIP1,GOT1L1,ADRB3,EIF4EBP1,ASH2L,STAR,LSM1,BAG4,DDHD2,PPAPDC1B,**WHSC1L1**,LETM2,**FGFR1**,FLJ43582,RNF5P1,LOC653218,TACC1,PLEKHA2,HTRA4,TM2D2,ADAM9,ADAM32,ADAM5,ADAM3A,LOC643197,ADAM18,ADAM2,INDO,LOC169355,C8orf4,ZMAT4,SFRP1,GOLGA7,LOC392214,SLD5,AGPAT6,FLJ25169,ANK1 |
| 8p11.21 | 41835520 | 42174630 | <0.001 | LOC643484,MYST3,AP3M2,PLAT |
| 8p11.21 | 42175206 | 42175362 | <0.001 | PLAT |
| 8p11.21 | 42183874 | 43782832 | <0.001 | PLAT,IKBKB,POLB,DKK4,VDAC3,SLC20A2,C8orf40,CHRNB3,CHRNA6,THAP1,RNF170,HOOK3,FNTA,FLJ23356,LOC441347,TMEM76,LOC643642,LOC643654,LOC347028,POTE8,LOC653343 |
| 8q11.1 | 47064078 | 47995266 | <0.001 | LOC389652 |
| 8q11.1 | 48030990 | 52450309 | <0.001 | RPL10AP2,LOC389654,LOC392217,CEBPD,LOC643975,PRKDC,MCM4,UBE2V2,LOC389655,FLJ46365,EFCAB1,LOC644334,SNAI2,C8orf22,C8orf62,SNTG1,CYCSP22,LOC644507,PXDNL |
| 8q11.22 | 52457559 | 53279035 | <0.001 | PXDNL,LOC644527,BTF3P1,PCMTD1,ST18 |
| 8q11.23 | 53279666 | 53290465 | <0.001 | ST18 |
| 8q11.23 | 53299334 | 54197442 | <0.001 | ST18,UNQ9433,RB1CC1,LOC644727,LOC644755,GPR7 |
| 8q11.23 | 54201590 | 54201931 | <0.001 | NA |
| 8q11.23 | 54209689 | 54837276 | <0.001 | OPRK1,MAPK6PS1,ATP6V1H |
| 8q11.23 | 54914761 | 55430165 | <0.001 | ATP6V1H,RGS20,TCEA1,LYPLA1,TDGF5,LOC644866,MRPL15,LOC392221 |
| 8q11.23 | 55452140 | 56078561 | <0.001 | SOX17,LOC392222,SEC11L2,RP1,LOC644986 |
| 8q12.1 | 56082799 | 57258362 | <0.001 | XKR4,LOC645012,TMEM68,NCOA6IP,LYN,PSMC6P,RPS20,RNU54,MOS,PLAG1 |
| 8q12.1 | 57296075 | 60607171 | <0.001 | LOC442387,RDHE2,LOC442388,PENK,LOC389662,RPL37P6,IMPAD1,LOC286177,C8orf71,T1560,C8orf72,LOC137886,CYP7A1,LOC137885,SDCBP,NSMAF,TOX |
| 8q12.1 | 60639227 | 60716348 | <0.001 | NA |
| 8q12.1 | 60716750 | 61340216 | <0.001 | LOC645408,CA8 |
| 8q12.1 | 61355704 | 61355761 | <0.001 | CA8 |
| 8q12.1 | 61369194 | 63965379 | <0.001 | LOC392225,RAB2,CHD7,LOC442389,NASPP1,NPM1P6,MGC34646,ASPH,LOC643730,LOC645551,LOC392226,FAM77D |
| 8q12.3 | 63970954 | 67597715 | <0.001 | FAM77D,LOC643763,GGH,TTPA,YTHDF3,IFITM8P,BHLHB5,CYP7B1,LOC645765,LOC392227,ARMC1,MTFR1,PDE7A,DNAJC5B,TRIM55,CRH,RRS1,ADHFE1,C8orf46 |
| 8q13.1 | 67740224 | 71556304 | <0.001 | VCPIP1,C8orf44,SGK3,PTTG3,C8orf45,LOC645929,LOC645936,LOC286187,COPS5,CSPP1,ARFGEF1,CPA6,DEPDC2,C8orf34,LOC389667,LOC646009,SULF1,SLCO5A1,PRDM14,H2AFZP2,NCOA2,LOC646067,LOC646063 |
| 8q13.3 | 71568374 | 72444849 | <0.001 | TRAM1,LACTB2,XKR9,EYA1 |
| 8q13.3 | 72454494 | 73478558 | <0.001 | MSC,TRPA1,LOC392232 |
| 8q13.3 | 73488434 | 73491353 | <0.001 | NA |
| 8q13.3 | 73523131 | 74145558 | <0.001 | KCNB2,TERF1,RPESP |
| 8q21.11 | 74177559 | 77138320 | <0.001 | LOC646197,LOC389669,RPL7,RDH10,LOC644201,STAU2,VENTXP6,LOC646235,UBE2W,TCEB1,TMEM70,LY96,JPH1,GDAP1,LOC286157,FLJ39080,PI15,CRISPLD1,HNF4G |
| 8q21.11 | 77199133 | 80401853 | <0.001 | MRPL9P1,LOC646345,ZFHX4,PXMP3,LOC646374,PKIA,C8orf70,IL7,LOC644363,LOC646399 |
| 8q21.13 | 80402202 | 81128294 | <0.001 | STMN2,HEY1,MRPS28,TPD52 |
| 8q21.13 | 81128385 | 83684014 | <0.001 | TPD52,LOC389671,LOC402342,LOC340443,ZBTB10,LOC389672,LOC646437,ZNF704,CKS1A,PAG1,LOC653745,LOC653746,LOC646463,FABP5,PMP2,LOC646480,FABP4,FTHL11,LOC646486,LOC646490,IMPA1,SLC10A5,ZFAND1,CHMP4C,SNX16,HNRPA1P4 |
| 8q21.13 | 83713599 | 84943217 | <0.001 | LOC646521,LOC646529 |
| 8q21.2 | 84967893 | 85017716 | <0.001 | LOC646537 |
| 8q21.2 | 85074614 | 85280456 | <0.001 | LOC646537,LOC138046 |
| 8q21.2 | 85298374 | 85298711 | <0.001 | LOC138046 |
| 8q21.2 | 85305933 | 88423104 | <0.001 | LOC138046,LRRCC1,E2F5,C8orf59,CA13,CA1,CA3,CA2,LOC653760,LOC392242,REXO1L3P,REXO1L1,LOC653042,REXO1L5P,REXO1L2P,REXO1L6P,REXO1L7P,LOC642320,PSKH2,ATP6V0D2,SLC7A13,WWP1,FAM82B,LOC642367,LOC642382,CPNE3,CNGB3,LOC642428,CNBD1 |
| 8q21.3 | 88508305 | 95372632 | <0.001 | LOC642461,SOX5P,WDR21C,LOC642514,MMP16,LOC642595,LOC642609,RIPK2,C8orf1,NBN,DECR1,CALB1,TMEM64,EFCBP1,LOC642730,TMEM55A,OTUD6B,LOC642767,LOC441368,SLC26A7,MRPS16P1,RUNX1T1,RPS26P10,LOC642866,FLJ46284,LOC642649,LOC389676,LOC642924,LOC642936,FAM92A1,RBM12B,TMEM67,LOC157667,PPM2C,CDH17,GEM |
| 8q22.1 | 95450132 | 108446732 | <0.001 | RAD54B,FSBP,KIAA1429,LOC643041,LOC643049,RBM35A,DPY19L4,C8orf52,CCNE2,TP53INP1,C8orf38,LOC653212,PLEKHF2,C8orf37,LOC643216,LOC643228,GDF6,UQCRB,MTERFD1,PTDSS1,SDC2,PGCP,TSPYL5,LOC286150,MTDH,LAPTM4B,RPS23P1,MATN2,RPL30,C8orf47,HRSP12,POP1,NPAL2,LOC643460,KCNS2,STK3,LOC643494,MRP63P7,LOC643506,LOC643514,OSR2,VPS13B,COX6C,RGS22,FBXO43,POLR2K,SPAG1,RNF19,ANKRD46,MGC39715,PABPC1,RPS26P6,YWHAZ,ZNF706,DUXAP2,NACAP1,GRHL2,NCALD,LOC643831,RRM2B,EDD1,ODF1,KLF10,FLJ45248,AZIN1,LOC442395,LOC643972,ATP6V1C1,BAALC,LOC644001,FZD6,CTHRC1,SLC25A32,WDSOF1,TARBP2P,RIMS2,TM7SF4,DPYS,LRP12,LOC644103,ZFPM2,LOC346887,OXR1,LOC643319,STARS,LOC644199,ANGPT1 |
| 8q23.1 | 108472658 | 119076457 | <0.001 | ANGPT1,RSPO2,LOC644233,EIF3S6,KIAA0103,TMEM74,TRHR,NUDCD1,ENY2,PKHD1L1,MAPK6PS5,EBAG9,FLJ20366,LOC644335,KCNV1,LOC392262,LOC644430,LOC644468,CSMD3,TRPS1,EIF3S3,C8orf53,RAD21,LOC644660,LOC441376,SLC30A8,THRAP6,EXT1 |
| 8q24.11 | 119097846 | 119097846 | <0.001 | EXT1 |
| 8q24.11 | 119116570 | 119955282 | <0.001 | EXT1,SAMD12,LOC441377 |
| 8q24.12 | 120007728 | 125041794 | <0.001 | TNFRSF11B,COLEC10,MAL2,NOV,LOC392264,ENPP2,CYCSP23,TAF2,DCC1,DEPDC6,COL14A1,MRPL13,MTBP,LOC643608,SNTB1,HAS2,MRPS36P3,LOC392265,ZHX2,DERL1,WDR67,FAM83A,C8orf76,ZHX1,LOC392266,ATAD2,LOC392267,C8orf32,FBXO32,C8ORFK36,ANXA13,FAM91A1 |
| 8q24.13 | 125052490 | 127306142 | <0.001 | C8orf54,FLJ32770,LOC392268,LOC442396,LOC645063,LOC392269,TMEM65,TRMT12,RNF139,TATDN1,NDUFB9,MTSS1,ZNF572,SQLE,KIAA0196,C8orf36,TRIB1 |
| 8q24.21 | 127309835 | 127309905 | <0.001 | NA |
| 8q24.21 | 127310450 | 128283681 | <0.001 | LOC645274,FAM84B,LOC645290,SRRM1L |
| 8q24.21 | 128350890 | 128351093 | <0.001 | NA |
| 8q24.21 | 128351336 | 128352364 | <0.001 | NA |
| 8q24.21 | 128357804 | 128550531 | <0.001 | POU5F1P1 |
| 8q24.21 | 128573679 | 128575517 | <0.001 | NA |
| 8q24.21 | 128577254 | 129821356 | <0.001 | **MYC**,PVT1,LOC441378 |
| 8q24.21 | 129856608 | 131356022 | <0.001 | CCDC26,MLZE,FAM49B,DDEF1 |
| 8q24.21 | 131356093 | 131356239 | <0.001 | DDEF1 |
| 8q24.21 | 131368653 | 132787960 | <0.001 | DDEF1,ADCY8 |
| 8q24.22 | 132843438 | 132847253 | <0.001 | NA |
| 8q24.22 | 132864960 | 132995952 | <0.001 | NA |
| 8q24.22 | 133011281 | 134720333 | <0.001 | KIAA0143,HHLA1,KCNQ3,LRRC6,TMEM71,PHF20L1,TG,SLA,WISP1,NDRG1,LOC392271,FAM10A6,ST3GAL1 |
| 8q24.22 | 134736775 | 135804194 | <0.001 | ZNF406,SAS-ZFAT |
| 8q24.22 | 135811513 | 137734990 | <0.001 | LOC645809,LOC286094,KHDRBS3 |
| 8q24.23 | 137735555 | 138362332 | <0.001 | LOC645921 |
| 8q24.23 | 138378921 | 138379138 | <0.001 | NA |
| 8q24.23 | 138379880 | 140595671 | <0.001 | FLJ45872,C8ORFK32,COL22A1 |
| 8q24.3 | 140607188 | 140792065 | <0.001 | KCNK9 |
| 8q24.3 | 140825354 | 141272686 | <0.001 | NIBP,LOC644167 |
| 8q24.3 | 141295342 | 141295342 | <0.001 | NIBP |
| 8q24.3 | 141303344 | 141930077 | <0.001 | NIBP,CHRAC1,LOC646107,EIF2C2,PTK2 |
| 8q24.3 | 142002911 | 142002911 | <0.001 | PTK2 |
| 8q24.3 | 142078709 | 146264218 | <0.001 | PTK2,DENND3,SLC45A4,GPR20,PTP4A3,FLJ43860,TSNARE1,BAI1,ARC,JRK,PSCA,LY6K,C8orf55,SLURP1,LYPD2,LYNX1,LY6D,GML,LOC646338,CYP11B1,CYP11B2,LY6E,C8orf31,LOC642276,LOC642295,HHCM,LY6H,LOC338328,ZFP41,GLI4,LOC642405,ZNF696,TOP1MT,C8orf51,RHPN1,MAFA,ZC3H3,GSDMDC1,LOC642475,NAPRT1,EEF1D,TIGD5,PYCRL,TSTA3,ZNF623,ZNF707,LOC286076,MAPK15,FLJ46072,LOC642564,LOC642574,SCRIB,SIAHBP1,NRBP2,LOC642584,PLEC1,PARP10,GRINA,SPATC1,LOC392275,OPLAH,LOC642602,EXOSC4,GPAA1,CYC1,SHARPIN,MAF1,KIAA1875,LOC642628,C8orf30A,LOC642638,KIAA1833,LOC653119,LOC642658,LOC653154,LOC642664,LOC642673,LOC642859,BOP1,LOC642876,HSF1,DGAT1,SCRT1,C8ORFK29,FBXL6,GPR172A,ADCK5,CPSF1,SLC39A4,VPS28,NFKBIL2,CYHR1,KIFC2,FOXH1,PPP1R16A,GPT,MFSD3,RECQL4,LRRC14,LRRC24,MGC70857,KIAA1688,ZNF251,ZNF34,RPL8,ZNF517,LOC642867,ZNF7,COMMD5,LOC642872,ZNF250,ZNF16,LOC642914,TMED10P,C8orf77,C8orf33 |
| 9p24.3 | 235706 | 2850773 | <0.001 | DOCK8,LOC645577,LOC645586,ANKRD15,LOC642350,DMRT1,DMRT3,DMRT2,SMARCA2,LOC645766,VLDLR,KCNV2,KIAA0020 |
| 9p24.2 | 2869563 | 3024259 | <0.001 | LOC138234 |
| 9p24.2 | 3058621 | 17626245 | <0.001 | RFX3,LOC645849,GLIS3,LOC645856,SLC1A1,C9orf68,PPAPDC2,CDC37L1,AK3,LOC392282,RCL1,JAK2,LOC642611,IGHEP2,INSL6,INSL4,RLN2,LOC645930,RLN1,C9orf46,CD274,PDCD1LG2,KIAA1432,KIAA1815,LOC645952,MLANA,KIAA2026,LOC441385,RANBP6,C9orf26,LOC645969,TPD52L3,UHRF2,C9orf38,GLDC,SNRPEL1,JMJD2C,LOC158345,LOC392285,LOC646041,C9orf123,**PTPRD**,RPS26P3,RN7SLP2,LOC646087,LOC646101,LOC646105,LOC646111,LOC646114,LOC646133,LOC646138,TYRP1,C9orf150,LOC646153,TDPX2,MPDZ,LOC646181,FLJ41200,LOC646206,LOC646211,LOC347193,LOC138864,NFIB,ZDHHC21,CER1,FREM1,LDHAL4,LOC389705,PSIP1P,C9orf52,LOC286348,SNAPC3,PSIP1,FTHL12,LOC646305,C9orf93,LOC646371,BNC2,MGC24103,C9orf39,SH3GL2 |
| 9p22.2 | 17626337 | 17626674 | <0.001 | SH3GL2 |
| 9p22.2 | 17645535 | 21460997 | <0.001 | SH3GL2,LOC646428,ADAMTSL1,C9orf94,C9orf138,PSMC3P,RRAGA,FAM29A,ADFP,LOC253482,C9orf55,RPS6,ASAH3L,LOC392288,SLC24A2,LOC646505,SMNP,MLLT3,KIAA1797,PTPLAD2,LOC646525,IFNB1,IFNW1,IFNA21,LOC392289,IFNA4,IFNA7,IFNA10,G13P1,LOC392291,IFNA16,IFNA17,LOC392292,IFNA14,IFNAP22,IFNA5,KLHL9,IFNA6,IFNA13,IFNA2,IFNWP12,IFNA8,LOC646581,IFNA1,IFNWP19 |
| 9p21.3 | 21486908 | 24413220 | <0.001 | LOC402359,MTAP,C9orf53,CDKN2A,CDKN2B,LOC646605,DMRTA1,FLJ35282,LOC646609,LOC646611,LOC402360,ELAVL2 |
| 9p21.3 | 24545849 | 27768662 | <0.001 | LOC646646,TUSC1,FLJ16323,C9orf82,PLAA,IFT74,LRRC19,TEK,C9orf14,C9orf11,MOBKL2B,IFNK,C9orf72,LOC392298 |
| 9p21.2 | 27792613 | 28577774 | <0.001 | LRRN6C,LOC646700,LOC653777 |
| 9p21.1 | 28670869 | 28701542 | <0.001 | LRRN6C |
| 9p21.1 | 28724248 | 28743170 | 0.001 | NA |
| 9p21.1 | 28831307 | 30038981 | 0.002 | LOC286239,LOC646734 |
| 9p21.1 | 30068773 | 30487203 | <0.001 | LOC401497 |
| 9p21.1 | 30502128 | 31143406 | 0.003 | LOC401497,LOC441391,LOC442405,LOC442406,LOC646753 |
| 9p21.1 | 31143852 | 31144475 | 0.001 | NA |
| 9p21.1 | 31154389 | 31914600 | 0.013 | LOC138412,LOC646768 |
| 9q22.2 | 92794541 | 92794650 | 0.042 | NA |
| 10q23.31 | 89672813 | 89830760 | 0.042 | **PTEN** |
| 11q14.1 | 81181573 | 81194806 | 0.006 | NA |
| 13q21.31 | 63177133 | 63204396 | 0.023 | NA |
| 14q11.2 | 19272965 | 19447566 | <0.001 | OR4Q3,OR4H12P,OR4M1,OR4N1P,OR4N2,OR4K6P,OR4K3P,OR4K2,OR4K4P |
| 14q11.2 | 19447789 | 19553467 | <0.001 | OR4K5,OR4K1,OR4K16P,OR4K15,OR4Q2P,OR4K14 |
| 14q11.2 | 19580947 | 20693676 | <0.001 | OR4U1P,OR4L1,OR4T1P,OR4K17,OR4N5,OR11G1P,OR11P1P,OR11G2,OR11H5P,OR11H6,OR11H7P,OR11H4,TTC5,CCNB1IP1,RPPH1,PARP2,TEP1,LOC123103,LOC441672,OSGEP,APEX1,TMEM55B,NP,RNASE10,RNASE9,RNASE11,RNASE12,LOC643145,LOC254028,OR6S1,RNASE4,ANG,RANBP20P,FAM12A,FAM12B,RNASE6,RNASE1,RNASE3,LOC643332,RNASE2,FLJ20859,SLC39A2,NDRG2,C14orf8,RNASE13,RNASE7,RNASE8,FLJ10357,ZNF219,LOC643382,LOC554207,OR5AU1 |
| 14q11.2 | 20696687 | 22075626 | <0.001 | LOC401744,HNRPC,RPGRIP1,MRPS17P6,SUPT16H,CHD8,UBA52P2,EIF4EBP1P,RAB2B,C14orf92,TRA@,TRAV1-1,TRAV1-2,TRAV2,TRAV3,TRAV4,TRAV5,TRAV6,TRAV7,TRAV8-1,TRAV9-1,TRAV10,TRAV11,TRAV12-1,TRAV8-2,TRAV8-3,TRAV13-1,TRAV12-2,TRAV8-4,TRAV8-5,TRAV13-2,TRAV14DV4,TRAV9-2,TRAV15,TRAV12-3,TRAV8-6,TRAV16,TRAV17,TRAV18,TRAV19,TRAV20,TRAV21,TRAV22,TRAV23DV6,TRDV1,TRAV24,TRAV25,TRAV26-1,TRAV8-7,TRAV27,TRAV28,TRAV29DV5,TRAV30,TRAV31,TRAV32,TRAV33,TRAV26-2,TRAV34,TRAV35,TRAV36DV7,TRAV37,TRAV38-1,TRAV38-2DV8,TRAV39,TRAV40,TRAV41,TRD@,TRDV2,TRDD1,TRDD2,TRDD3,TRDJ1,TRDJ4,TRDJ2,TRDJ3,TRDC,TRDV3,TRAJ61,TRAJ60,TRAJ59,TRAJ58,TRAJ57,TRAJ56,TRAJ55,TRAJ54,TRAJ53,TRAJ52,TRAJ51,TRAJ50,TRAJ49,TRAJ48,TRAJ47,TRAJ46,TRAJ45,TRAJ44,TRAJ43,TRAJ42,TRAJ41,TRAJ40,TRAJ39,TRAJ38,TRAJ37,TRAJ36,TRAJ35,TRAJ34,TRAJ33,TRAJ32,TRAJ31,TRAJ30,TRAJ29,TRAJ28,TRAJ27,TRAJ26,TRAJ25,TRAJ24,TRAJ23,TRAJ22,TRAJ21,TRAJ20,TRAJ19,TRAJ18,TRAJ17,TRAJ16,TRAJ15,TRAJ14,TRAJ13,TRAJ12,TRAJ11,TRAJ10,TRAJ9,TRAJ8 |
| 14q11.2 | 22087370 | 23342964 | <0.001 | TRA@,TRAC,DAD1,ABHD4,OR6J1,RPL26P2,OR6E1P,LOC643639,OXA1L,SLC7A7,MRPL52,MMP14,LRP10,FLJ38964,RBM23,SKB1,TRR,C14orf94,JUB,C14orf93,PSMB5,FLJ16369,CDH24,ACIN1,C14orf119,LOC643712,CEBPE,SLC7A8,RPL39P2,HMGN2P,KIAA1443,PPP1R3E,BCL2L2,PABPN1,SLC22A17,EFS,LOC643759,IL17E,CMTM5,MYH6,MYH7,C14orf120,ZFHX2,ZNF409,THTPA,AP1G2,JPH4,DHRS2,BRD7P |
| 14q11.2 | 23351860 | 24029893 | <0.001 | LOC643819,C14orf165,DHRS4,DHRS4L2,LOC400197,C14orf121,CPNE6,NRL,PCK2,WDR23,LOC161247,PSME1,C14orf122,PSME2,RNF31,ISGF3G,REC8L1,IPO4,TM9SF1,TSSK4,CHMP4A,MGC5987,NEDD8,GMPR2,TINF2,TGM1,RABGGTA,DHRS1,C14orf21,CIDEB,LTB4R2,LTB4R,ADCY4,RIPK3,NFATC4,KIAA1305,LOC643866,KIAA0323,C14orf124 |
| 14q11.2 | 24040413 | 26937626 | <0.001 | CMA1,CTSG,GZMH,GZMB,STXBP6,LOC643872,OR7K1P,LOC401767,NOVA1,UNGP2,RPS27AP4 |
| 14q12 | 26961103 | 34427101 | <0.001 | LOC643990,BNIP3P,RPL26P3,BTF3P2,FOXG1B,LOC387978,LOC644095,PRKD1,SYF2P,KIAA1333,SCFD1,UBE2CP1,RPL12P5,RPL27P1,COCH,STRN3,AP4S1,HECTD1,NARSP,ATP5GP4,C14orf126,LOC644223,GPR33,NUBPL,C14orf128,ARHGAP5,LOC644295,AKAP6,MTCO1P2,NPAS3,EGLN3,LOC644347,LOC644360,C14orf147,LOC644384,C14orf11,SNX6,RPL23AP9,CFL2,RPL12P6,BAZ1A |
| 14q13.1 | 34451372 | 34999946 | <0.001 | LOC390466,LOC338902,LOC441682,SRP54,C14orf24,C14orf10,KIAA0391,CDC10P,MRP63P8,DPRXP3,PSMA6,LOC122589,NFKBIA,DNAJC8P1 |
| 14q13.1 | 35006343 | 37538344 | <0.001 | LOC122592,INSM2,GARNL1,NUTF2P2,BRMS1L,LOC644584,LOC644616,MBIP,STELLAR,TITF1,PHKBP2,NKX2-8,FLJ42220,LOC644719,PAX9,SLC25A21,MIPOL1,LOC644765,FOXA1,TTC6 |
| 14q13.3 | 37608411 | 38772080 | <0.001 | SSTR1,CLEC14A,LOC390470,LOC283547,SEC23A,PPIAP4,SIP1,TRAPPC6B,PNN,YTHDF2P |
| 14q21.1 | 38776492 | 41443589 | <0.001 | MIA2,CTAGE5,COILP,FBXO33,LOC644898,LOC644919,LRFN5,LOC644977 |
| 14q21.1 | 41484830 | 45190488 | <0.001 | YWHAQP,TUBBP3,HNRPUP,LOC390472,ARHGAP16P,EIF4BP,LOC645086,YWHAZP,C14orf155,LOC401770,LOC401772,C14orf28,BTBD5,KIAA0423,PRPF39,FKBP3,FANCM,C14orf106,LOC644589,LOC645149 |
| 14q21.2 | 45270503 | 45297574 | <0.001 | NA |
| 14q21.2 | 45371549 | 46288404 | <0.001 | LOC645168,RPL10L |
| 14q21.2 | 46403821 | 49817087 | <0.001 | MAMDC1,RPA2P,RPL13AP2,RPS15AP3,LOC645211,RPL18P1,ATP5GP2,RPS29,PPIL5,RHOQP,RPL36AL,MGAT2,C14orf104,POLE2,STK16P,KLHDC1,KLHDC2,SDCCAG1,ARF6,LOC283551,PDLIM1P,RPS15AP2,LOC196913,C14orf138,SOS2,L2HGDH |
| 14q21.3 | 49854669 | 51385013 | <0.001 | ATP5S,CDKL1,MAP4K5,SPG3A,SNRPGP,SAV1,ZNF405P,NIN,C14orf29,PYGL,MRP63P9,TRIM9,TXNDC,LOC283553,SETP2,LOC645380,C14orf82,FRMD6,LOC645393,OR7E105P,OR7E106P,OR7E159P |
| 14q22.1 | 51390854 | 60068293 | <0.001 | GNG2,C14orf166,NID2,COX5AP2,LOC645417,PTGDR,PTGER2,KIAA1344,DKFZp762F0713,ERO1L,PSMC6,STYX,GNPNAT1,PLEKHC1,NDUFB3P3,DDHD1,LOC645496,BMP4,ATP5C2,LOC645560,CDKN3,CNIH,GMFB,CGRRF1,LOC645602,LOC644925,SAMD4,GCH1,WDHD1,SOCS4,C14orf32,LGALS3,GALIG,DLG7,UBE2L7P,LOC645637,FBXO34,LOC653555,KIAA0831,HMGN1P1,TBPL2,ABI1P,KTN1,LOC645683,LOC645687,PELI2,C14orf101,LOC645776,OTX2,LOC645798,LOC440180,LOC645814,EXOC5,C14orf108,C14orf35,C14orf105,SLC35F4,LOC401777,C14orf37,UBA52P3,ACTR10,PSMA3,HMGB1P,ARID4A,UNQ9438,TIMM9,KIAA0586,HSBP1P1,HNRPCP,DACT1,RPL31P4,LOC440181,LOC390482,PPIAP5,DAAM1,GPR135,C14orf149,C14orf100,RTN1,FLJ46156,C14orf135,DHRS7,PSMA3P,PPM1A,RBM8B,C14orf39,LOC390483,SIX6 |
| 14q23.1 | 60085227 | 61014730 | <0.001 | SIX1,SIX4,MNAT1,MAD2L1P,SRMP2,LOC653695,TRMT5,SLC38A6,TMEM30B,PRKCH |
| 14q23.1 | 61015992 | 64131739 | <0.001 | PRKCH,LOC400221,HIF1A,SNAPC1,LOC122867,MOCS3P,SYT16,LOC401778,LOC646110,LOC646113,LOC646121,KCNH5,PARP1P2,RHOJ,GPHB5,PPP2R5E,LOC646150,GCATP,C14orf150,HSPEP2,SGPP1,EIF2S2P,SYNE2,ESR2,LOC441687,MTHFD1,AKAP5,ZBTB25,ZBTB1,HSPA2,C14orf50 |
| 14q23.2 | 64159117 | 66704071 | <0.001 | PLEKHG3,SPTB,LOC653716,CHURC1,GPX2,RAB15,FNTB,MAX,LOC646279,RPL36AP2,PTBP1P,FUT8,LOC645431,RPL21P8,NCOA4P,LOC646362,YBX1P1,MGC88374,GPHN |
| 14q23.3 | 66708208 | 67134787 | <0.001 | GPHN,C14orf54,LOC260329,MPP5,ATP6V1D,EIF2S1,PLEK2,FLJ33387,PLEKHH1,PIGH |
| 14q23.3 | 67188499 | 73056667 | <0.001 | VTI1B,COX7AP1,RDH11,RPL21P9,RDH12,ZFYVE26,RAD51L1,RPL7AP5,PPIAP6,RPL12P7,ZFP36L1,FLJ39779,MAGOHP,BLZF2P,ACTN1,RPS29P1,WDR22,DDX18P1,C14orf114,GALNTL1,LOC653751,ERH,SLC39A9,FLJ44817,LOC646511,RPL24P3,C14orf162,KIAA0247,SFRS5,SLC10A1,LOC646541,SMOC1,RPL7AP6,SLC8A3,LOC646548,ADAM21P,C14orf112,SYNJ2BP,ADAM21,C14orf55,ADAM20,MED6,TTC9,MAP3K9,PCNX,PTTG4P,RNU56B,SIPA1L1,RGS6,DPF3,LOC645728,WDR21A,RPL36AP3,RPS12P1,ZFYVE1,RBM25,PSEN1,PAPLN,NUMB,MGC48595,C14orf169 |
| 14q24.2 | 73075681 | 73105424 | <0.001 | MGC48595,ACOT1,NT5CP2 |
| 14q24.2 | 73136565 | 73808045 | <0.001 | ACOT6,NDUFB8P1,C14orf168,PNMA1,C14orf43,LOC646631,ZADH1,ZNF410,C14orf44,COQ6,ENTPD5,RPL41P4,LOC646642,C14orf45,ALDH6A1,C14orf46,CHX10 |
| 14q24.3 | 73814930 | 91724557 | <0.001 | ABCD4,C14orf115,RPS2P2,LOC646658,NPC2,RAP1AP,HBLD1,LTBP2,KIAA0317,C14orf111,YLPM1,DLST,RPS6KL1,PGF,EIF2B2,MLH3,ACYP1,C14orf140,NEK9,TMED10,FOS,LOC646701,JDP2,BATF,C14orf58,RPS24P2,C14orf1,TTLL5,TGFB3,MGC16028,C14orf118,ESRRB,CYCSP1,RPSAP3,VASH1,ANGEL1,C14orf166B,LOC646765,RPL22P2,RPLP1P1,C14orf4,KIAA1737,ZDHHC22,TMEM63C,NGB,POMT2,GSTZ1,TMED8,C14orf174,C14orf148,C14orf133,AHSA1,THSD3,SPTLC2,ALKBH,RPL21P10,C14orf156,SNW1,FLJ25976,ADCK1,FRDAP,NRXN3,DIO2,C14orf145,HMGN2P2,TSHR,RPL17P3,NMNATP,GTF2A1,LOC246720,UNGP3,STN2,RPS24P3,LOC388002,SEL1L,LOC646867,LOC646874,RPL9P6,EIF3S6IPP,LOC646883,ENSAP2,RNU3P3,LOC646919,FLRT2,LOC646924,LOC283584,LOC646926,LOC283585,GALC,GPR65,KCNK10,SPATA7,PTPN21,FLJ11806,EML5,TTC8,LOC390501,LOC653795,CHES1,LOC646224,CAP2P1,LOC400236,PRO1768,CHORDC2P,C14orf143,LOC646996,TDP1,KCNK13,GLRXP2,PSMC1,C14orf102,RPL21P11,CALM1,TTC7B,RPS18P2,RPS6KA5,C14orf159,GPR68,KIAA1509,KIAA2010,LOC647032,C14orf161,NANOGP7,MTAC2D1,FBLN5,TRIP11,PTMAP7,ATXN3,NDUFB1,CPSF2 |
| 14q32.11 | 91739589 | 93700484 | <0.001 | SLC24A4,RIN3,LGMN,GOLGA5,CHGA,ITPK1,CYB5P3,MOAP1,C14orf142,C14orf130,RPL18AP1,BTBD7,RPL36AP4,KIAA1409,COX8C,LOC653810,PRIMA1,C14orf152,ASB2,C14orf48,OTUB2,LOC653813,DDX24,FAM14B,IFI27,FAM14A |
| 14q32.12 | 93750927 | 94401384 | <0.001 | KIAA1622,SERPINA10,SERPINA6,SERPINA2,SERPINA1,SERPINA11,SERPINA9,SERPINA12,SERPINA4,SERPINA5,SERPINA3,LOC390503,SERPINA13,RPSAP4,GSC,RPL15P2 |
| 14q32.12 | 94431099 | 97884159 | <0.001 | DICER1,LOC647116,FLJ45244,CLMN,C14orf139,C14orf49,C14orf62,GLRX5,TCL6,TCL1B,TCL1A,LOC647137,C14orf132,BDKRB2,CKS1BP,BDKRB1,C14orf103,C14orf129,AK7,PBPP1,RPL23AP10,PAPOLA,VRK1,LOC647197 |
| 14q32.2 | 97902070 | 98387885 | <0.001 | FLJ25773 |
| 14q32.2 | 98394205 | 99148653 | <0.001 | RPL3P4,BCL11B,C14orf154,CCNK,LOC647237 |
| 14q32.2 | 99174082 | 102504492 | <0.001 | KIAA1822,CYP46A1,LOC647240,EML1,LOC341965,RPS2P3,EVL,DEGS2,YY1,SLC25A29,C14orf68,WARS,NDUFB3P4,WDR25,KIAA1446,C14orf70,DLK1,LOC647251,MEG3,LOC388015,LOC646590,LOC647254,FLJ41170,DIO3,LOC122423,RPL26P4,C14orf72,PPP2R5C,NPM1P20,DYNC1H1,HSPCA,WDR20,RAGE,C14orf131,CINP,KIAA0329,ANKRD9,RCOR1,RPL23AP11,TRAF3,AMN,CDC42BPB |
| 14q32.31 | 102505936 | 102554578 | <0.001 | CDC42BPB |
| 14q32.31 | 102567591 | 102596316 | <0.001 | CDC42BPB |
| 14q32.31 | 102636538 | 104419597 | <0.001 | C14orf73,TNFAIP2,LOC647273,RPL21P12,RPL21P13,LOC441698,RPL17P4,EIF5,RPSAP5,MARK3,RPL10AP1,CKB,C14orf172,BAG5,C14orf153,KNS2,XRCC3,ZFYVE21,PPP1R13B,C14orf2,LOC647286,TDRD9,LOC374569,KIF26A,C14orf144,LOC400258,FLJ42486,C14orf151,C14orf173,LOC388022,ADSSL1,SIVA,AKT1,RPS2P4,KIAA0284 |
| 14q32.33 | 104527433 | 106318151 | <0.001 | C14orf79,CDCA4,GPR132,LOC647308,JAG2,NUDT14,BRF1,BTBD6,PACS2,LOC647310,MTA1,CRIP2,CRIP1,C14orf80,LOC647311,MGC4659,ATP5GP3,ELK2P2,IGHA2,IGHE,IGHG4,IGHG2,IGHGP,ELK2P1,IGHA1,IGHEP1,IGHG1,IGHG3,IGHD,IGHM,IGHJ6,IGHJ3P,IGHJ5,IGHJ4,IGHJ3,IGHJ2P,IGHJ2,IGHJ1,IGHD7-27,IGHJ1P,IGHD1-26,IGHD6-25,IGHD5-24,IGHD4-23,IGHD3-22,IGHD2-21,IGHD1-20,IGHD6-19,IGHD5-18,IGHD4-17,IGHD3-16,IGHD2-15,IGHD1-14,IGHD6-13,IGHD5-12,IGHD4-11,IGHD3-10,IGHD3-9,IGHD2-8,IGHD1-7,IGHD6-6,IGHD5-5,IGHD4-4,IGHD3-3,IGHD2-2,IGHD1-1,KIAA0125,IGHV6-1,IGHVII-1-1,ADAM6,LOC192133,IGHV1-2,IGHVIII-2-1,IGHV1-3,IGHV4-4,IGHV2-5,IGHVIII-5-1,IGHVIII-5-2,IGHV3-6,IGHV3-7,IGHV1-8,IGHV3-9,IGHV2-10,IGHV3-11,IGHVIII-11-1,IGHV1-12,IGHV3-13,IGHVIII-13-1,IGHV1-14,IGHV3-15,IGHVII-15-1,IGHV3-16,IGHVIII-16-1,IGHV1-17,LOC192128,IGHV1-18,IGHV3-19,IGHV3-20,IGHVII-20-1,IGHV3-21,IGHV3-22,IGHVII-22-1,IGHVIII-22-2,IGHV3-23,IGHV1-24,IGHV3-25,IGHVIII-25-1,IGHV2-26,IGHVIII-26-1,IGHVII-26-2,IGHV7-27,IGHV4-28,IGHVII-28-1,IGHV3-29,LOC192132,IGHV3-30,IGHVII-30-1,IGHV3-30-2,IGHV4-31,IGHVII-31-1,IGHV3-32,LOC192131,IGHV3-33,IGHVII-33-1,IGHV3-33-2,IGHV4-34,IGHV7-34-1,IGHV3-35,IGHV3-36,IGHV3-37,IGHV3-38,IGHVIII-38-1,IGHV4-39,IGHV7-40,IGHVII-40-1,IGHV3-41,IGHV3-42,IGHV3-43,IGHVII-43-1,IGHVIII-44,IGHVIV-44-1,IGHVII-44-2,IGHV1-45,IGHV1-46,IGHVII-46-1,IGHV3-47,IGHVIII-47-1,IGHV3-48,IGHV3-49,IGHVII-49-1,IGHV3-50,IGHV5-51,IGHVIII-51-1,IGHVII-51-2,IGHV3-52,IGHV3-53,IGHVII-53-1,IGHV3-54,IGHV4-55,IGHV7-56,IGHV3-57,IGHV1-58,IGHV4-59,IGHV3-60,IGHVII-60-1,IGHV4-61,IGHV3-62,IGHVII-62-1,IGHV3-63,LOC192130,IGHV3-64,IGHV3-65,IGHVII-65-1,IGHV3-66,IGHV1-67,LOC192127,IGHVII-67-1,IGHVIII-67-2,IGHVIII-67-3,IGHVIII-67-4,IGHV1-68,IGHV1-69,IGHV2-70,IGHV3-71,IGHV3-72,IGHV3-73,IGHV3-74,IGHVII-74-1,IGHV3-75,IGHV3-76,IGHVIII-76-1 |
| 15q13.1 | 30297184 | 30298847 | 0.028 | NA |
| 16p13.11 | 16170651 | 16170862 | <0.001 | ABCC6 |
| 16q23.1 | 77434802 | 77434919 | 0.03 | WWOX |
| 16q23.3 | 81840807 | 81840912 | <0.001 | CDH13 |
| 17q11.2 | 26520469 | 26570301 | 0.012 | NF1 |
| 17q22 | 51518046 | 51522852 | <0.001 | NA |
| 17q25.1 | 74757811 | 74757985 | 0.018 | LOC146713 |
| 18q11.2 | 24449765 | 24450128 | <0.001 | NA |
| 18q22.1 | 62060664 | 62060697 | <0.001 | NA |
| 18q22.1 | 62060963 | 62060963 | 0.011 | NA |
| 18q22.1 | 64902064 | 64902105 | 0.041 | NA |
| 18q22.1 | 64902464 | 76104900 | <0.001 | DOK6,CD226,RTTN,SOCS6,RPS2P6,GTSCR1,LOC643734,LOC643765,CBLN2,NETO1,LOC388481,LOC400655,FBXO15,C18orf55,CYB5,LOC644041,FAUP1,C18orf51,CNDP2,CNDP1,LOC400657,ZNF407,ZADH2,SDCCAG33,LOC284274,ZNF516,FLJ44313,FLJ44881,LOC644657,ZNF236,MBP,LOC642534,GALR1,LOC645144,LOC645321,SALL3,ATP9B,LOC653054,LOC653063,LOC653069,NFATC1,FLJ25715,CTDP1,LOC645411,KCNG2,PQLC1,LOC440498,TXNL4A,C18orf22,KIAA0863,PARD6G |
| 19q13.32 | 47380527 | 47527532 | 0.029 | DEDD2,ZNF526,GSK3A,ERF,CIC,PAFAH1B3,MGC70924,FLJ90805 |
| 21q22.2 | 41332614 | 41332735 | 0.032 | NA |
