# Supplementary material for: Genome-wide identification of significant aberrations in cancer genome
Source: BMC Genomics. 2012 Jul 27;13:342. doi: 10.1186/1471-2164-13-342 (PMC3428679; doi:10.1186/1471-2164-13-342)
Supplement: Additional file 3 — Table S4 and Table S5. Details about the implicated SCAs and full list of genes covered by these SCAs, derived from the prostate cancer data set. [file 1471-2164-13-342-S3.doc]

**Supplementary Table 4.** Details about the implicated SCAs and full list of genes covered by these SCAs, derived from genome-wide analysis of the prostate cancer data set. (Start: Start point of SCAs. End: End point of SCAs.) (Based on hg18 human genome assembly)

| **Cyto-**  **band** | **Region** | | **P-value** | **Genes covered by the SCA regions** |
| --- | --- | --- | --- | --- |
|  | **Start** | **End** |  |  |
| ***Amplification*** | | | | |
| 1q31.1 | 185996586 | 185996586 | 0.017 | NA |
| 3q21.3 | 127195881 | 131182049 | 0.021 | SLC41A3,ALDH1L1,LOC644662,KLF15,CCDC37,ZXDC,UROC1,CHST13,C3orf22,LOC645840,LOC255330,LOC645852,CHCHD6,LOC644738,PLXNA1,FLJ40141,LOC645994,GPR175,MCM2,PODXL2,ABTB1,MGLL,LOC653521,KLHDC6,SEC61A1,RUVBL1,EEFSEC,DNAJB8,GATA2,C3orf27,FLJ40473,RPN1,RAB7,FTHL4,LOC391574,LOC646147,LOC653712,LOC646158,ACAD9,KIAA1257,LOC401087,CCDC48,GP9,RAB43,KIAA1160,ZNF9,COPG,C3orf37,H1FX,LOC339942,LOC646226,LOC132241,LOC646238,C3orf25,MBD4,IFT122,RHO,H1FOO,PLXND1,TMCC1,LOC644947,TRH |
| 7p21.3 | 8990771 | 10370805 | 0.032 | LOC340268,LOC646033 |
| 7p12.1 | 51735404 | 55153330 | 0.001 | LOC642878,LOC392027,DKFZp564N2472,LOC653175,FLJ45974,LOC222005,MGC33530,LOC392030,SEC61G,LOC643168,**EGFR** |
| 8p11.23 | 37835998 | 41651413 | <0.001 | RAB11FIP1,GOT1L1,ADRB3,EIF4EBP1,ASH2L,STAR,LSM1,BAG4,DDHD2,PPAPDC1B,WHSC1L1,LETM2,FGFR1,FLJ43582,RNF5P1,LOC653218,TACC1,PLEKHA2,HTRA4,TM2D2,ADAM9,ADAM32,ADAM5,ADAM3A,LOC643197,ADAM18,ADAM2,INDO,LOC169355,C8orf4,ZMAT4,SFRP1,GOLGA7,LOC392214,SLD5,AGPAT6,FLJ25169,ANK1 |
| 8p11.21 | 41654876 | 41654876 | <0.001 | ANK1 |
| 8p11.21 | 41659228 | 42773387 | 0.002 | ANK1,LOC643484,MYST3,AP3M2,PLAT,IKBKB,POLB,DKK4,VDAC3,SLC20A2,C8orf40,CHRNB3,CHRNA6 |
| 8q11.21 | 48990637 | 49046076 | 0.042 | PRKDC,MCM4 |
| 8q12.1 | 56119911 | 56204747 | 0.009 | XKR4 |
| 8q12.1 | 58510755 | 63479655 | 0.007 | T1560,C8orf72,LOC137886,CYP7A1,LOC137885,SDCBP,NSMAF,TOX,LOC645408,CA8,LOC392225,RAB2,CHD7,LOC442389,NASPP1,NPM1P6,MGC34646,ASPH,LOC643730,LOC645551,LOC392226,FAM77D |
| 8q12.3 | 63490044 | 63490044 | 0.005 | FAM77D |
| 8q12.3 | 65355691 | 68291691 | 0.001 | BHLHB5,CYP7B1,LOC645765,LOC392227,ARMC1,MTFR1,PDE7A,DNAJC5B,TRIM55,CRH,RRS1,ADHFE1,C8orf46,MYBL1,LOC645895,VCPIP1,C8orf44,SGK3,PTTG3,C8orf45,LOC645929,LOC645936,LOC286187,COPS5,CSPP1,ARFGEF1 |
| 8q13.2 | 68324257 | 68710014 | 0.016 | ARFGEF1,CPA6 |
| 8q13.2 | 68712746 | 69970425 | <0.001 | CPA6,DEPDC2,C8orf34 |
| 8q13.2 | 70099697 | 70363152 | 0.003 | LOC389667 |
| 8q13.2 | 70381191 | 71115041 | <0.001 | LOC646009,SULF1,SLCO5A1 |
| 8q13.3 | 71123789 | 71308654 | 0.004 | PRDM14,H2AFZP2,**NCOA2**,LOC646067 |
| 8q13.3 | 71323788 | 73441136 | <0.001 | NCOA2,LOC646067,LOC646063,TRAM1,LACTB2,XKR9,EYA1,MSC,TRPA1,LOC392232 |
| 8q13.3 | 73640656 | 73994428 | 0.002 | KCNB2 |
| 8q21.11 | 74001540 | 74535315 | 0.002 | KCNB2,TERF1,RPESP,LOC646197,LOC389669,RPL7,RDH10,LOC644201 |
| 8q21.11 | 74581833 | 75730305 | <0.001 | STAU2,VENTXP6,LOC646235,UBE2W,TCEB1,TMEM70,LY96,JPH1,GDAP1,LOC286157,FLJ39080 |
| 8q21.11 | 75733990 | 75742020 | 0.008 | FLJ39080 |
| 8q21.11 | 75750235 | 76213440 | <0.001 | FLJ39080,PI15,CRISPLD1 |
| 8q21.11 | 76562540 | 77343156 | <0.001 | HNF4G |
| 8q21.11 | 77468429 | 78108433 | <0.001 | MRPL9P1,LOC646345,ZFHX4,PXMP3 |
| 8q21.12 | 79101643 | 79793146 | <0.001 | LOC646374,PKIA,C8orf70 |
| 8q21.12 | 79826622 | 79970312 | 0.005 | IL7,LOC644363 |
| 8q21.12 | 80049709 | 81579586 | <0.001 | LOC646399,STMN2,HEY1,MRPS28,TPD52,LOC389671,LOC402342,LOC340443,ZBTB10 |
| 8q21.13 | 81594333 | 81594333 | 0.024 | ZBTB10 |
| 8q21.13 | 81605168 | 81645040 | 0.004 | LOC389672 |
| 8q21.13 | 81658479 | 83718279 | <0.001 | ZNF704,CKS1A,PAG1,LOC653745,LOC653746,LOC646463,FABP5,PMP2,LOC646480,FABP4,FTHL11,LOC646486,LOC646490,IMPA1,SLC10A5,ZFAND1,CHMP4C,SNX16,HNRPA1P4 |
| 8q21.13 | 84130171 | 84240387 | 0.006 | LOC646529 |
| 8q21.2 | 84722655 | 85425642 | 0.002 | LOC646537,LOC138046 |
| 8q21.2 | 85434281 | 87046380 | 0.001 | LOC138046,LRRCC1,E2F5,C8orf59,CA13,CA1,CA3,CA2,LOC653760,LOC392242,REXO1L3P,REXO1L1,LOC653042,REXO1L5P,REXO1L2P,REXO1L6P,REXO1L7P |
| 8q21.3 | 87060228 | 87722435 | 0.004 | LOC642320,PSKH2,ATP6V0D2,SLC7A13,WWP1,FAM82B,LOC642367,LOC642382,CPNE3,CNGB3 |
| 8q21.3 | 87737354 | 88353990 | 0.004 | CNGB3,LOC642428,CNBD1 |
| 8q21.3 | 88365900 | 88508943 | 0.033 | CNBD1 |
| 8q21.3 | 88678369 | 88769616 | 0.029 | LOC642461 |
| 8q21.3 | 88783438 | 89112004 | 0.006 | SOX5P,WDR21C,LOC642514 |
| 8q21.3 | 89935863 | 90112490 | 0.023 | NA |
| 8q21.3 | 90126188 | 90670216 | 0.002 | LOC642595,LOC642609 |
| 8q21.3 | 90677565 | 91247921 | 0.004 | RIPK2,C8orf1,NBN,DECR1,CALB1 |
| 8q21.3 | 91253458 | 91372309 | 0.048 | NA |
| 8q21.3 | 92178849 | 92226337 | 0.05 | LOC642767 |
| 8q21.3 | 92244241 | 92534061 | 0.013 | LOC642767,SLC26A7 |
| 8q21.3 | 92589049 | 92657981 | 0.006 | NA |
| 8q21.3 | 92705562 | 93638710 | 0.002 | MRPS16P1,RUNX1T1,RPS26P10 |
| 8q22.1 | 93653568 | 93887791 | 0.014 | LOC642866,FLJ46284 |
| 8q22.1 | 94089357 | 95542532 | 0.001 | LOC642649,LOC389676,LOC642924,LOC642936,FAM92A1,RBM12B,TMEM67,LOC157667,PPM2C,CDH17,GEM,LOC643022,RAD54B,FSBP |
| 8q22.1 | 95847847 | 95964031 | 0.005 | DPY19L4,C8orf52,CCNE2 |
| 8q22.1 | 95965282 | 96425423 | 0.024 | CCNE2,TP53INP1,C8orf38,LOC653212,PLEKHF2,C8orf37 |
| 8q22.1 | 96427151 | 96486715 | 0.008 | NA |
| 8q22.1 | 96914315 | 97748531 | 0.013 | LOC643216,LOC643228,GDF6,UQCRB,MTERFD1,PTDSS1,SDC2,PGCP |
| 8q22.1 | 97959674 | 100709521 | <0.001 | PGCP,TSPYL5,LOC286150,MTDH,LAPTM4B,RPS23P1,MATN2,RPL30,C8orf47,HRSP12,POP1,NPAL2,LOC643460,KCNS2,STK3,LOC643494,MRP63P7,LOC643506,LOC643514,OSR2,VPS13B |
| 8q22.2 | 100718959 | 101731897 | 0.007 | VPS13B,COX6C,RGS22,FBXO43,POLR2K,SPAG1,RNF19,ANKRD46,MGC39715 |
| 8q22.3 | 102714833 | 104657256 | 0.026 | GRHL2,NCALD,LOC643831,RRM2B,EDD1,ODF1,KLF10,FLJ45248,AZIN1,LOC442395,LOC643972,ATP6V1C1,BAALC,LOC644001,FZD6,CTHRC1,SLC25A32,WDSOF1 |
| 8q23.1 | 107563179 | 109625791 | 0.014 | OXR1,LOC643319,STARS,LOC644199,ANGPT1,RSPO2,LOC644233,EIF3S6,KIAA0103 |
| 8q24.13 | 125469382 | 126936888 | 0.018 | TRMT12,RNF139,TATDN1,NDUFB9,MTSS1,ZNF572,SQLE,KIAA0196,C8orf36,TRIB1 |
| 8q24.21 | 127627710 | 128050431 | <0.001 | FAM84B,LOC645290 |
| 8q24.21 | 128054062 | 128328101 | <0.001 | SRRM1L |
| 8q24.21 | 128489740 | 128574847 | 0.005 | POU5F1P1 |
| 8q24.21 | 128581886 | 129019540 | <0.001 | **MYC**,PVT1 |
| 8q24.21 | 129021490 | 129026680 | 0.01 | PVT1 |
| 8q24.22 | 132258606 | 133683844 | 0.032 | KIAA0143,HHLA1,KCNQ3,LRRC6 |
| 8q24.22 | 133912574 | 134239192 | 0.032 | PHF20L1,TG,SLA |
| 8q24.22 | 134242719 | 136827333 | <0.001 | WISP1,NDRG1,LOC392271,FAM10A6,ST3GAL1,ZNF406,SAS-ZFAT,LOC645809,LOC286094,KHDRBS3 |
| 8q24.3 | 140129217 | 141788433 | 0.041 | **KCNK9**,NIBP,C8orf17,LOC644167,CHRAC1,LOC646107,EIF2C2,PTK2 |
| 8q24.3 | 141791266 | 142074020 | 0.013 | PTK2 |
| 12q23.2 | 101941518 | 101941518 | <0.001 | NA |
| ***Deletion*** | | | | |
| 2q22.1 | 138935721 | 139042511 | 0.037 | LOC339745 |
| 2q22.2 | 143355079 | 146440590 | 0.022 | KYNU,ARHGAP15,LOC647040,GTDC1,ZFHX1B,SGCEP,LOC647045 |
| 5q15 | 94071954 | 97076117 | 0.013 | MCTP1,LOC642563,FAM81B,KIAA0372,DKFZp313G1735,GPR150,LOC317671,SPATA9,RHOBTB3,GLRX,FIS,ELL2,LOC441097,PCSK1,CAST,ARTS-1,LOC642716,LRAP,LNPEP,LOC642737,LIX1,RIOK2,YTHDF1P |
| 5q15 | 98135041 | 99078160 | 0.001 | RGMB,LOC642690,CHD1,LOC441066 |
| 5q21.1 | 99310734 | 99317820 | 0.01 | LOC285706 |
| 5q21.1 | 99322441 | 99563108 | 0.012 | LOC643020,LOC643031,LOC643047,LOC643053 |
| 5q21.1 | 102511430 | 102516658 | 0.024 | HISPPD1 |
| 6q14.1 | 82475389 | 82483196 | 0.048 | NA |
| 6q14.1 | 83057555 | 85644061 | 0.002 | TPBG,C6orf157,KIAA1117,PGM3,RWDD2,ME1,PRSS35,SNAP91,LOC401268,C6orf159,CYB5R4,LOC643761,C6orf117,C6orf84,LOC442233,TBX18 |
| 6q14.3 | 85665503 | 87466492 | 0.002 | LOC643851,LOC643858,LOC643870,NT5E,SNX14,SYNCRIP,LOC643883,C6orf160,LOC643906,LOC643916,LOC643926 |
| 6q14.3 | 87466743 | 87467387 | 0.018 | NA |
| 6q14.3 | 87517384 | 90596729 | <0.001 | LOC643962,HTR1E,LOC643971,CGA,LOC442234,ZNF292,GJB7,LOC644016,C6orf162,C6orf163,C6orf165,TAF13P,SLC35A1,RARSL,ORC3L,C6orf166,SPACA1,CNR1,LOC644119,ACTBP8,RNGTT,CYCSP16,PNRC1,SRrp35,ACY1L2,GABRR1,GABRR2,UBE2J1,RRAGD,ANKRD6,DJ122O8.2,MDN1,CASP8AP2 |
| 6q15 | 90602901 | 91033654 | 0.001 | CASP8AP2,LOC644269,CX62,BACH2,LOC653299 |
| 6q15 | 91098822 | 91185400 | 0.004 | NA |
| 6q15 | 91188213 | 92784316 | 0.002 | MAP3K7 |
| 6q15 | 92788711 | 92794166 | 0.025 | NA |
| 6q16.1 | 93135142 | 93141944 | 0.009 | NA |
| 6q16.1 | 93146799 | 96387563 | <0.001 | LOC135270,EPHA7,LOC643432,LOC644562,LOC644569,LOC644579,CYCSP17,MANEA |
| 6q16.1 | 96395392 | 96938848 | 0.01 | LOC442236,FUT9 |
| 6q16.1 | 97795795 | 98984201 | 0.039 | C6orf167 |
| 6q16.1 | 99219429 | 100050723 | 0.05 | POU3F2,FBXL4,DHRS6P1,C6orf168,COQ3,C6orf111,USP45 |
| 6q16.3 | 104847916 | 109283510 | <0.001 | HACE1,LOC643075,LIN28B,BVES,POPDC3,PREP,LOC643132,PRDM1,ATG5,AIM1,RTN4IP1,QRSL1,LOC553137,C6orf203,KIAA1553,C6orf210,FLJ10159,SCML4,SEC63,LOC643297,LOC642741,OSTM1,NR2E1,SNX3,LACE1,FOXO3A,LOC442240,ARMC2 |
| 8p23.3 | 242541 | 2390262 | 0.036 | LOC644147,FBXO25,C8orf42,LOC389607,ERICH1,C8orf68,LOC401442,LOC644319,LOC644327,LOC644421,DLGAP2,CLN8,C8orf61,ARHGEF10,LOC644668,KBTBD11,LOC644711,MYOM2 |
| 8p23.2 | 2694603 | 3569825 | 0.032 | CSMD1 |
| 8p23.2 | 4185927 | 5020596 | 0.032 | CSMD1,LOC392179 |
| 8p23.1 | 8177800 | 9078513 | 0.001 | DKFZp761P0423,CLDN23,MFHAS1,MRPS18CP2,LOC645960,THEX1,RNU7P4,PPP1R3B |
| 8p23.1 | 9085514 | 9097487 | 0.014 | NA |
| 8p23.1 | 9101214 | 9433870 | 0.003 | LOC645986 |
| 8p23.1 | 9436786 | 9935652 | <0.001 | TNKS |
| 8p23.1 | 9943681 | 11047079 | 0.001 | MSRA,LOC346702,UNQ9391,RP1L1,LOC203076,SOX7,PINX1,XKR6,C8orf15,C8orf16 |
| 8p23.1 | 11048140 | 11048140 | 0.001 | XKR6 |
| 8p23.1 | 11049265 | 11894615 | 0.002 | XKR6,LOC392193,MTMR9,AMAC1L2,TDH,C8orf13,BLK,GATA4,C8orf49,NEIL2,FDFT1,CTSB,OR7E158P,OR7E161P,DEFB137,DEFB136,DEFB134 |
| 8p23.1 | 12526350 | 13172923 | 0.006 | LOC653337,LOC646354,OR7E8P,OR7E15P,OR7E10P,LONRF1,FLJ36980,KIAA1456,DLC1 |
| 8p22 | 13202283 | 13290055 | 0.034 | DLC1 |
| 8p22 | 15467675 | 16233555 | 0.035 | **TUSC3**,LOC137012,LOC646433,MSR1,LOC646440 |
| 8p22 | 18381158 | 18619920 | 0.041 | LOC653754,PSD3 |
| 8p21.3 | 19129945 | 20545220 | 0.001 | LOC442382,SH2D4A,ChGn,C8orf35,LPL,SLC18A1,ATP6V1B2,LZTS1,RNU3P2 |
| 8p21.3 | 20557869 | 21757654 | 0.013 | LOC646608,GFRA2,LOC653765,OR6R2P |
| 8p21.3 | 21793664 | 22549069 | 0.003 | DOK2,XPO7,NPM2,FGF17,EPB49,RAI16,NUDT18,HR,C8orf20,LGI3,SFTPC,BMP1,PHYHIP,LOC646654,POLR3D,PIWIL2,SLC39A14,PPP3CC,SORBS3,PDLIM2,C8orf58,KIAA1967,BIN3 |
| 8p21.3 | 22555957 | 23458187 | 0.008 | BIN3,EGR3,PEBP4,RHOBTB2,TNFRSF10B,TNFRSF10C,TNFRSF10D,TNFRSF10A,LOC389641,CHMP7,R3HCC1,LOXL2,ENTPD4,LOC646708,SLC25A37 |
| 8p21.2 | 23463719 | 23463719 | <0.001 | SLC25A37 |
| 8p21.2 | 25373608 | 25465999 | 0.004 | CDCA2 |
| 8p21.2 | 25473693 | 25721789 | 0.004 | NA |
| 8p21.2 | 25744347 | 26058435 | 0.003 | EBF2 |
| 8p21.2 | 26063811 | 27083769 | <0.001 | PPP2R2A,LOC157489,BNIP3L,LOC440258,PNMA2,LOC338097,DPYSL2,ADRA1A,LOC646818 |
| 8p21.2 | 27092641 | 27403111 | 0.001 | STMN4,TRIM35,PTK2B,CHRNA2 |
| 8p21.1 | 27414422 | 27759128 | 0.036 | EPHX2,GULOP,CLU,SCARA3,LOC646843,CCDC25,ESCO2,PBK |
| 8p21.1 | 28341990 | 31035169 | 0.006 | FBXO16,FZD3,LOC653787,EXTL3,RC74,FLJ21616,KIF13B,DUSP4,LOC646909,MAP2K1P1,TMEM66,LEPROTL1,DCTN6,LOC392209,LOC642319,LOC92755,RBPMS,LOC642356,GTF2E2,GSR,LOC642388,UBXD6,PPP2CB,TEX15,LOC441344,PURG,WRN |
| 13q12.3 | 31857240 | 32647306 | 0.029 | **BRCA2**,IFIT1P,CG018,LOC88523,PFAAP5,APRIN,LOC122038,KL,STARD13 |
| 13q13.3 | 39762538 | 40237618 | 0.021 | LOC646982,LOC646990,FOXO1A,MRPS31 |
| 13q14.11 | 40253776 | 40850091 | <0.001 | SLC25A15,CYCSP34,LOC643729,ELF1,WBP4,KBTBD6,CALM2P3,KBTBD7,MTRF1,RAC1P3,NARG1L |
| 13q14.11 | 40856723 | 43329268 | <0.001 | LOC647000,OR7E36P,OR7E155P,LOC647007,RGC32,KIAA0564,DGKH,MAPK6PS3,LOC341651,LOC647031,AKAP11,FABP3P2,TNFSF11,FLJ40919,EPSTI1,DNAJC15,PIG38,LOC647049,LOC647050,FLJ31846 |
| 13q14.11 | 43332991 | 43345243 | 0.001 | FLJ31846 |
| 13q14.11 | 43348346 | 46241573 | <0.001 | FLJ31846,FLJ38725,LOC400126,MGC5590,C13orf21,TSC22D1,LOC400129,LOC144817,LOC647075,NUFIP1,KIAA1704,GTF2F2,KCTD4,TPT1,LOC644007,SLC25A30,LOC647080,LOC647085,COG3,FLJ32682,LOC390402,NURIT,LOC283514,LOC647092,KIAA0853,CPB2,LCP1,LOC220416,LOC390403,C13orf18,LOC653817,OR7E101P,LOC81993,LRCH1 |
| 13q14.13 | 46261024 | 46545433 | 0.004 | ESD,HTR2A |
| 13q14.13 | 46662596 | 47796150 | 0.001 | LOC647113,SUCLA2,NUDT15,MED4,ITM2B,**RB1** |
| 13q14.2 | 47796308 | 48565255 | 0.001 | RB1,P2RY5,RCBTB2,LOC644250,CYSLTR2,LOC338099,LOC647131,FNDC3A,RAD17P2 |
| 13q14.2 | 48570055 | 48570055 | <0.001 | FNDC3A |
| 13q14.2 | 48577158 | 48651886 | 0.023 | FNDC3A |
| 13q14.2 | 48665291 | 53172163 | <0.001 | FNDC3A,LOC387924,MLNR,CDADC1,CAB39L,SETDB2,PHF11,RCBTB1,ARL11,EBPL,KPNA3,LOC220429,C13orf1,RFP2OS,RFP2,KCNRG,DLEU2,DLEU1,FLJ31945,FAM10A4,LOC647154,DLEU7,FLJ11712,GUCY1B2,LOC647166,LOC341674,FLJ30707,LOC647174,DDX26,LOC220433,LOC647181,WDFY2,FLJ13639,FLJ37307,DKFZP434K1172,LOC400135,ATP7B,LOC440138,UTP14C,LOC387927,FKSG49,NEK3,THSD1P,LOC644623,THSD1,VPS36,CKAP2,LOC220115,LOC653821,LOC144983,SUGT1,LECT1,LOC121981,PCDH8,OLFM4,LOC647207 |
| 13q14.3 | 53286053 | 55668871 | <0.001 | LOC647213,LOC387930,LOC647221 |
| 13q21.1 | 55673052 | 55675988 | 0.001 | NA |
| 13q21.1 | 55684077 | 57304063 | 0.001 | LOC647226,FLJ40296,LOC653823,LOC653824,LOC653825,LOC653826,LOC647245,PCDH17,LOC647248 |
| 13q21.1 | 57308436 | 57313488 | 0.02 | NA |
| 13q21.1 | 57326578 | 58264156 | 0.005 | LOC387931,LOC341689 |
| 13q21.1 | 58270722 | 59441434 | 0.001 | DIAPH3 |
| 13q21.1 | 59571180 | 62793221 | <0.001 | LOC440142,TDRD3,LOC390407,LOC647256,LOC647257,PCDH20,LOC647258,LOC647259,LOC647260,LOC647261 |
| 13q21.31 | 63152858 | 63623521 | 0.017 | OR7E156P,LOC647262,LOC401737,LOC647264,OR7E104P,LOC647266 |
| 13q21.31 | 64482043 | 64938903 | 0.05 | STARP1,LOC647269 |
| 13q21.32 | 66399798 | 67050860 | 0.006 | PCDH9,LOC400141,LOC647271 |
| 13q21.32 | 67054339 | 67391185 | 0.037 | LOC647272,LOC390411,OR7E111P,OR7E33P |
| 13q21.33 | 69437327 | 69639399 | 0.006 | KLHL1 |
| 13q21.33 | 70374817 | 71325768 | 0.005 | LOC647277,DACH1 |
| 13q21.33 | 71328893 | 71329368 | 0.03 | DACH1 |
| 13q21.33 | 71336188 | 73919504 | <0.001 | DACH1,LOC440145,FLJ22624,KIAA1008,C13orf24,LOC338091,KLF5,LOC387934,LOC647279,LOC647281,KLF12,LOC400145,LOC647283 |
| 13q22.1 | 73928013 | 74499864 | 0.044 | LOC122145 |
| 16q21 | 64154321 | 68916485 | 0.026 | LOC283867,CDH5,BEAN,TK2,CKLF,CMTM1,CMTM2,CMTM3,CMTM4,DYNC1LI2,FLJ35894,APPBP1,CA7,LOC644978,PDP2,CDH16,RRAD,FAM96B,CES2,FLJ21736,FLJ37464,CBFB,LIN10,MGC4655,TRADD,FBXL8,HSF4,NOL3,LOC653319,LOC283849,E2F4,ELMO3,LRRC29,LOC653563,HSPC171,FHOD1,SLC9A5,PLEKHG4,KCTD19,LRRC36,CGI-38,ZDHHC1,HSD11B2,ATP6V0D1,AGRP,FAM65A,CTCF,RLTPR,ACD,PARD6A,C16orf48,LOC388284,MGC11335,RANBP10,TSNAXIP1,C16orf56,THAP11,NUTF2,LOC645138,RCD-8,UNQ2446,PSKH1,CTRL,PSMB10,LCAT,SLC12A4,DPEP3,DPEP2,DDX28,DUS2L,NFATC3,RBM35B,LYPLA3,SLC7A6,SLC7A6OS,PRMT7,SMPD3,LOC643895,ZFP90,CDH3,CDH1,LOC645198,FLJ12688,FLJ12331,HAS3,DERPC,CIRH1A,SNTB2,VPS4A,COG8,PDF,NIP7,TMED6,TERF2,CYB5-M,NFAT5,NQO1,NOB1P,LOC644035,WWP2,LOC348174,LOC645282,LOC645287,LOC283970,LOC440349,LOC645299,LOC645307,PDPR,MGC34761,LOC645325,EXOSC6,AARS,DDX19B |
| 16q22.1 | 70223486 | 76929958 | <0.001 | MARVELD3,PHLPPL,AP1G1,BOAT,LOC146517,LOC55565,KIAA0174,PKD1L3,LOC645443,DHODH,HP,HPR,TXNL4B,DHX38,PMFBP1,LOC390739,LOC645478,LOC342374,**ATBF1**,C16orf47,LOC441506,LOC645656,LOC401859,PSMD7,LOC440386,LOC440348,LOC441773,LOC497190,GLG1,LOC645713,RFWD3,LOC645726,MLKL,FA2H,WDR59,ZNRF1,LDHD,ZFP1,LOC441774,CTRB2,CTRB1,BCAR1,CFDP1,LOC124491,CHST6,LOC645799,CHST5,GABARAPL2,ADAT1,KARS,TERF2IP,LOC645821,LOC401860,LOC124496,LOC401861,LOC441775,CNTNAP4,LOC645873,MON1B,ADAMTS18,LOC645919,KIAA1576,CLEC3A,LOC342419,WWOX |
| 16q23.1 | 76943009 | 77617653 | 0.001 | WWOX,LOC645947,LOC645957 |
| 16q23.1 | 77621174 | 78264125 | 0.002 | WWOX,MAF |
| 16q23.1 | 78273703 | 78447460 | 0.048 | LOC440389 |
| 16q23.1 | 78452403 | 79558131 | <0.001 | DYNLRB2,CDYL2 |
| 16q23.2 | 79567994 | 81262670 | <0.001 | DC13,BM039,ASCIZ,C16orf46,GCSH,PKD1L2,BCMO1,GAN,CMIP,PLCG2,HSPC105,HSD17B2,MPHOSPH6,CDH13 |
| 16q23.3 | 82328664 | 84263652 | <0.001 | CDH13,HSBP1,MLYCD,OKL38,EFCBP2,LOC146167,MBTPS1,HSDL1,LRRC50,TAF1C,LOC161931,KCNG4,WFDC1,KIAA0703,KIAA1609,COTL1,C16orf44,USP10,CRISPLD2,LOC123862,ZDHHC7,KIAA0513,FAM92B,LOC123855,MGC22001,KIAA0182 |
| 16q24.1 | 84494244 | 85492716 | 0.011 | IRF8,DKFZp434O0320,LOC401864,FOXF1,LOC401865,FLJ12998,FLJ30679,FOXC2,FOXL1 |
| 16q24.1 | 85498166 | 86180580 | 0.001 | FBXO31,MAP1LC3B,ZCCHC14 |
| 16q24.1 | 86305699 | 86312893 | 0.028 | KLHDC4 |
| 16q24.1 | 86493061 | 86632428 | 0.017 | CA5A,BANP |
| 16q24.1 | 86639405 | 86678930 | 0.003 | BANP |
| 16q24.1 | 86687750 | 87412581 | 0.003 | LOC646638,ZNF469,ZFPM1,FLJ45530,NHN1,IL17C,CYBA,MVD,SNAI3,RNF166,LOC348180,FAM38A,FLJ45121,FLJ40448,CDT1,APRT,GALNS |
| 16q24.2 | 87844146 | 88123607 | 0.011 | ANKRD11,SPG7 |
| 17p13.2 | 5150699 | 7384709 | 0.001 | RABEP1,NUP88,RIP,C1QBP,DHX33,DERL2,MIS12,NALP1,LOC643333,LOC643340,KIAA0523,AIPL1,FAM64A,PITPNM3,KIAA0753,LOC643501,TXNL5,MED31,LOC342531,SLC13A5,BIRC4BP,FBXO39,TEKT1,ALOX12P2,ALOX12,MGC71993,C17orf49,BCL6B,SLC16A13,SLC16A11,CLEC10A,ASGR2,ASGR1,DLG4,ACADVL,DVL2,PHF23,GABARAP,DULLARD,DERP6,CLDN7,SLC2A4,YBX2,EIF5A,GPS2,KIAA1787,LOC390760,CENTB1,KCTD11,TMEM95,TNK1,PLSCR3,C17orf61,NLGN2,LOC374768,C17orf74,TMEM102,FGF11,CHRNB1,ZBTB4,LOC643664,POLR2A |
| 17p13.1 | 7394416 | 7501560 | 0.01 | TNFSF12,TNFSF12-TNFSF13,TNFSF13,SENP3,EIF4A1,CD68,MPDU1,SOX15,FXR2,SAT2,SHBG,ATP1B2 |
| 17p13.1 | 7524779 | 7981116 | 0.003 | **TP53**,WDR79,EFNB3,DNHD3,JMJD3,TMEM88,LSMD1,CYB5D1,CHD3,LOC284023,KCNAB3,TRAPPC1,LIP8,GUCY2D,ALOX15B,ALOX12B,ALOXE3,TRR1,HES7 |
| 17p13.1 | 8399249 | 13377432 | <0.001 | MYH10,LOC643933,CCDC42,LOC388333,FLJ35773,C17orf38,PIK3R5,NTN1,STX8,WDR16,USP43,DHRS7C,LOC644070,GLP2R,RCV1,GAS7,LOC644008,RPS27AP1,MYH13,MYH8,MYH4,MYH1,MYH2,MYH3,SCO1,C17orf48,LOC388335,LOC644139,FLJ45455,DNAH9,ZNF18,MAP2K4,FLJ34690,MYOCD,KIAA0672,ELAC2,HS3ST3A1 |
| 21q22.13 | 38811655 | 41764479 | <0.001 | **ERG,**C21orf24,ETS2,FLJ45139,LOC391282,PCBP2P1,DSCR2,BRWD1,C21orf87,HMGN1,WRB,C21orf13,SH3BGR,C21orf88,B3GALT5,LOC150084,PCP4,DSCAM,LOC645279,BACE2,PLAC4,FAM3B,MX2,MX1,**TMPRSS2** |
| 21q22.2 | 41764724 | 41773324 | <0.001 | TMPRSS2 |

**Supplementary Table 5.** Details about the implicated SCAs and full list of genes covered by these SCAs, derived from individual chromosome analysis of the prostate cancer data set. (Start: Start point of SCAs. End: End point of SCAs.) (Based on hg18 human genome assembly)

| **Cyto-**  **band** | **Region** | | **P-value** | **Genes covered by the SCA regions** |
| --- | --- | --- | --- | --- |
|  | **Start** | **End** |  |  |
| ***Amplification*** | | | | |
| 1q31.1 | 185996410 | 185996586 | 0.017 | NA |
| 3q21.3 | 127195881 | 131186255 | <0.001 | SLC41A3,ALDH1L1,LOC644662,KLF15,CCDC37,ZXDC,UROC1,CHST13,C3orf22,LOC645840,LOC255330,LOC645852,CHCHD6,LOC644738,PLXNA1,FLJ40141,LOC645994,GPR175,MCM2,PODXL2,ABTB1,MGLL,LOC653521,KLHDC6,SEC61A1,RUVBL1,EEFSEC,DNAJB8,GATA2,C3orf27,FLJ40473,RPN1,RAB7,FTHL4,LOC391574,LOC646147,LOC653712,LOC646158,ACAD9,KIAA1257,LOC401087,CCDC48,GP9,RAB43,KIAA1160,ZNF9,COPG,C3orf37,H1FX,LOC339942,LOC646226,LOC132241,LOC646238,C3orf25,MBD4,IFT122,RHO,H1FOO,PLXND1,TMCC1,LOC644947,TRH |
| 3q22.1 | 131289615 | 133409207 | 0.014 | LOC644974,LOC646286,LOC440978,LOC646300,FLJ35880,LOC131873,PIK3R4,GSTO3P1,ATP2C1,ASTE1,NEK11,LOC152195,NUDT16,MRPL3,CPNE4,LOC402142 |
| 3q22.2 | 134369566 | 136172659 | 0.004 | TMEM108,BFSP2,LOC391578,LOC653740,CDV3,TOPBP1,LOC646432,TF,SRPRB,RAB6B,C3orf36,SLCO2A1,RYK,AMOTL2,LOC646493,ANAPC13,CEP63,KY,EPHB1,LOC645218 |
| 5p15.33 | 877449 | 2452908 | <0.001 | ZDHHC11,BRD9,TRIP13,NKD2,SLC12A7,SLC6A19,SLC6A18,TERT,CRR9,SLC6A3,AYTL2,LOC653102,LOC642535,LOC653378,LOC442128,MRPL36,NDUFS6,LOC389267,LOC644065,IRX4 |
| 5p15.33 | 2453149 | 3491519 | <0.001 | IRX2,CEI,LOC285577 |
| 5p15.33 | 3502329 | 4053067 | <0.001 | LOC285577,IRX1 |
| 5p15.32 | 4917168 | 6277202 | 0.002 | LOC340094,ADAMTS16,LOC442131,KIAA0947 |
| 5p15.32 | 6277626 | 7064885 | 0.004 | LOC645267,FLJ33360,TRG20,FLJ25076,NSUN2,SRD5A1,POLS |
| 5p15.31 | 7068360 | 7996960 | <0.001 | LOC645463,LOC645451,LOC442132,ADCY2,LOC134121,MGC5297,MTRR |
| 5p15.31 | 7998453 | 8986733 | <0.001 | LOC645502,LOC645583,LOC645607 |
| 5p15.31 | 9084052 | 9322658 | 0.019 | SEMA5A |
| 5p15.31 | 9610248 | 10248154 | 0.015 | TAS2R1 |
| 5p15.2 | 14364103 | 14753265 | 0.003 | TRIO,FAM105A,LOC645894,LOC391739,EEF1AL11,FAM105B |
| 5p15.1 | 18373224 | 18514272 | 0.001 | NA |
| 5p14.3 | 22667014 | 23820035 | 0.028 | CDH12,LOC391771,LOC646393,LOC646398,PRDM9 |
| 5p14.1 | 26388762 | 31038366 | 0.005 | LOC646504,CDH9,LOC643401,LOC646568,PGBD3P2,HPRTP2 |
| 8p11.23 | 37835460 | 41652324 | <0.001 | RAB11FIP1,GOT1L1,ADRB3,EIF4EBP1,ASH2L,STAR,LSM1,BAG4,DDHD2,PPAPDC1B,WHSC1L1,LETM2,FGFR1,FLJ43582,RNF5P1,LOC653218,TACC1,PLEKHA2,HTRA4,TM2D2,ADAM9,ADAM32,ADAM5,ADAM3A,LOC643197,ADAM18,ADAM2,INDO,LOC169355,C8orf4,ZMAT4,SFRP1,GOLGA7,LOC392214,SLD5,AGPAT6,FLJ25169,ANK1 |
| 8p11.21 | 41652496 | 41654876 | 0.018 | ANK1 |
| 8q13.2 | 68712691 | 69970425 | 0.014 | CPA6,DEPDC2,C8orf34 |
| 8q13.2 | 70377661 | 71115293 | 0.016 | LOC646009,SULF1,SLCO5A1 |
| 8q13.3 | 71323788 | 73443436 | <0.001 | **NCOA2**,LOC646067,LOC646063,TRAM1,LACTB2,XKR9,EYA1,MSC,TRPA1,LOC392232 |
| 8q21.11 | 74573011 | 75730305 | 0.005 | STAU2,VENTXP6,LOC646235,UBE2W,TCEB1,TMEM70,LY96,JPH1,GDAP1,LOC286157,FLJ39080 |
| 8q21.11 | 75743005 | 76215118 | 0.001 | FLJ39080,PI15,CRISPLD1 |
| 8q21.11 | 76562540 | 77348962 | 0.03 | HNF4G |
| 8q21.12 | 80049516 | 81579586 | 0.01 | LOC646399,STMN2,HEY1,MRPS28,TPD52,LOC389671,LOC402342,LOC340443,ZBTB10 |
| 8q21.13 | 81658479 | 83719671 | 0.023 | ZNF704,CKS1A,PAG1,LOC653745,LOC653746,LOC646463,FABP5,PMP2,LOC646480,FABP4,FTHL11,LOC646486,LOC646490,IMPA1,SLC10A5,ZFAND1,CHMP4C,SNX16,HNRPA1P4 |
| 8q24.21 | 127621008 | 128051271 | 0.021 | FAM84B,LOC645290 |
| 8q24.21 | 128053617 | 128328101 | 0.003 | SRRM1L |
| 8q24.21 | 128581886 | 129019911 | 0.001 | MYC,PVT1 |
| 9p23 | 10555759 | 10555977 | 0.008 | NA |
| 10q21.1 | 52980984 | 52990374 | 0.027 | PRKG1 |
| 10q21.1 | 54789249 | 54790949 | 0.015 | NA |
| 10q21.1 | 54798140 | 57111441 | <0.001 | LOC387683,PCDH15,LOC644679,LOC389970 |
| 10q21.1 | 57112632 | 59348730 | <0.001 | ZWINT,LOC644763 |
| 10q21.1 | 59348871 | 60816937 | <0.001 | MRPS35P3,IPMK,C10orf70,UBE2D1,TFAM,BICC1,LOC644871,PHYHIPL,FAM13C1 |
| 10q21.1 | 60819241 | 63329050 | <0.001 | MRPL50P4,SLC16A9,LOC644883,LOC644886,CCDC6,ANK3,CDC2,RHOBTB1,LOC644918,TMEM26,C10orf107,LOC644954 |
| 10q21.2 | 63392751 | 64529569 | 0.003 | ARID5B,PLEKHK1,ZNF365,ATQL4,C10orf22,EGR2 |
| 10q21.3 | 65181631 | 65306783 | 0.017 | NA |
| 10q21.3 | 65318842 | 65487863 | 0.002 | RPL7AP1 |
| 10q21.3 | 65488279 | 65700561 | 0.045 | LOC645084 |
| 10q21.3 | 65700653 | 66262081 | <0.001 | ANXA2P3 |
| 10q21.3 | 66264671 | 66507784 | <0.001 | LOC645123 |
| 10q21.3 | 67131633 | 69570246 | 0.004 | CTNNA3,LRRTM3,LOC340888,LOC441563,DNAJC12,LOC645161,SIRT1,HERC4,MYPN |
| 10q22.1 | 73516278 | 75002867 | 0.026 | SPOCK2,ASCC1,C10orf104,DDIT4,DNAJB12,CBARA1,LOC645547,C10orf42,OIT3,PLA2G12B,P4HA1,NUDT13,HSGT1,DNAJC9,MRPS16,TTC18,ANXA7,ZMYND17,PPP3CB,USP54 |
| 12q23.2 | 101941518 | 101941606 | <0.001 | NA |
| 17q24.3 | 68738589 | 69760107 | 0.002 | FAM104A,HLC-8,LOC642843,CDC42EP4,SDK2,C17orf54,RPL38,TTYH2 |
| 17q25.1 | 72383312 | 72918048 | 0.036 | MGAT5B,SEC14L1,CYCSP40,SEPT9 |
| 17q25.1 | 73363061 | 74299746 | <0.001 | FLJ45079,TNRC6C,TMC6,TMC8,EIF5AP2,SYNGR2,TK1,AFMID,BIRC5,EPR1,LOC283999,THA1P,SOCS3,DNAH17,DNAHL1,PSCD1,USP36 |
| 17q25.1 | 74300073 | 74324435 | 0.04 | USP36 |
| 17q25.1 | 74758786 | 75686776 | <0.001 | LOC146713,LOC643798,ENPP7,CBX2,CBX8,CBX4,TBC1D16,CCDC40 |
| 17q25.3 | 77652621 | 77906874 | 0.012 | FLJ23754,CCDC57,SLC16A3,CSNK1D,CD7,SECTM1 |
| 17q25.3 | 77909585 | 78643088 | 0.036 | FLJ35767,UTS2R,FLJ22222,HEXDC,C17orf62,NARF,FOXK2,WDR45L,RAB40B,FN3KRP,FN3K,TBCD,FLJ13841,B3GNTL1,LOC644193,METRNL |
| 18q12.3 | 39104721 | 40558284 | <0.001 | SYT4,LOC646971,LOC646988,LOC342732,SETBP1 |
| 18q21.1 | 46825128 | 46858172 | 0.003 | SMAD4 |
| 22q11.1 | 14886276 | 14886461 | 0.003 | NA |
| 22q11.1 | 16203283 | 16538344 | 0.009 | LOC644899,CLCP1,CECR2,SLC25A18,ATP6V1E1,BCL2L13 |
| 22q11.21 | 20712109 | 20712345 | 0.004 | IGL@ |
| Xq11.1 | 62199072 | 63781720 | <0.001 | LOC645251,LOC139886,ARHGEF9,HNRPDP,FLJ39827,ASB12,MTMR8,LOC392481,LOC645338,LOC442455 |
| Xq11.2 | 63783289 | 64414068 | <0.001 | KIAA1166 |
| Xq11.2 | 64415812 | 65732077 | <0.001 | LOC645374,LOC645381,LOC645388,ZC3H12B,LAS1L,FKSG43,MSN,NANOGP9,LOC645420,VSIG4,LOC392485,LOC645430,HEPH,GPR165P,LOC402408 |
| Xq12 | 65734571 | 67178644 | <0.001 | EDA2R,LOC645456,AR |
| Xq12 | 67180018 | 67265749 | <0.001 | OPHN1,PGK1P1 |
| Xq12 | 67268637 | 68627638 | <0.001 | OPHN1,LOC643374,YIPF6,STARD8,SERBP1P,EFNB1,PJA1,CYCSP43 |
| ***Deletion*** | | | | |
| 1p21.3 | 95277695 | 95278423 | 0.048 | ALG14 |
| 2q14.3 | 127735925 | 129927966 | 0.006 | ERCC3,MAP3K2,PROC,FLJ10006,MYO7B,LIMS2,GPR17,SFT2D3,WDR33,POLR2D,MGC4268,LOC646509,SAP130,UGCGL1,HS6ST1,LOC646551,LOC151121 |
| 2q21.3 | 135470744 | 136972322 | 0.013 | YSK4,RAB3GAP1,ZRANB3,LOC646959,R3HDM1,UBXD2,LCT,MCM6,LOC391448,DARS,CXCR4,LOC389053,UBBP1 |
| 2q22.1 | 137882531 | 137896118 | 0.019 | KIAA1679 |
| 2q22.1 | 138232306 | 138870154 | 0.014 | HNMT,LOC440917,LOC647002 |
| 2q22.1 | 138934468 | 139042511 | <0.001 | LOC339745 |
| 2q22.1 | 139046886 | 139244712 | 0.006 | LOC339745,NXPH2 |
| 2q22.1 | 139247553 | 139661000 | 0.029 | NXPH2,LOC647012,LOC129560 |
| 2q22.2 | 142546764 | 142680842 | 0.013 | LRP1B |
| 2q22.2 | 143214378 | 143354309 | 0.001 | KYNU |
| 2q22.2 | 143355039 | 146441687 | <0.001 | KYNU,ARHGAP15,LOC647040,GTDC1,ZFHX1B,SGCEP,LOC647045 |
| 2q22.3 | 147014448 | 149601170 | <0.001 | LOC200583,ACVR2A,ORC4L,LOC647065,MBD5,EPC2,UBBP3,LOC647079,LOC647083,KIF5C |
| 5q14.1 | 77810254 | 80790380 | 0.013 | SCAMP1,LHFPL2,ARSB,DMGDH,BHMT2,BHMT,LOC644872,JMY,LOC643932,HOMER1,LOC133748,PAPD4,CMYA5,LOC644895,MTX3,THBS4,SERINC5,LOC391803,LOC644936,SPZ1,LOC391804,CRSP8,LOC644037,ZFYVE16,UNQ9217,DP58,DBIL1,DHFR,MSH3,RASGRF2,CKMT2,ZCCHC9,ACOT12,SSBP2 |
| 5q14.3 | 88140940 | 89047631 | 0.044 | MEF2C,LOC645370 |
| 5q14.3 | 92169279 | 93241727 | 0.024 | LOC391810,LOC391811,FLJ42709,NR2F1,DKFZP564D172,FLJ25680,FLJ12078 |
| 5q15 | 93410303 | 93819651 | 0.008 | DKFZP564D172,LOC642488,KIAA0825 |
| 5q15 | 94064751 | 94877918 | <0.001 | MCTP1,LOC642563,FAM81B,KIAA0372 |
| 5q15 | 94881794 | 94883012 | 0.001 | KIAA0372 |
| 5q15 | 94885922 | 97076449 | <0.001 | KIAA0372,DKFZp313G1735,GPR150,LOC317671,SPATA9,RHOBTB3,GLRX,FIS,ELL2,LOC441097,PCSK1,CAST,ARTS-1,LOC642716,LRAP,LNPEP,LOC642737,LIX1,RIOK2,YTHDF1P |
| 5q15 | 97125441 | 97731642 | <0.001 | PSME2P,LOC402221 |
| 5q15 | 97751722 | 98094368 | <0.001 | MRPS35P2,LOC642909,LOC402222 |
| 5q15 | 98129578 | 100717024 | <0.001 | RGMB,LOC642690,CHD1,LOC441066,LOC285706,LOC643020,LOC643031,LOC643047,LOC643053,LOC643066,LOC441098,LOC643074,UNQ1912,ST8SIA4 |
| 5q21.1 | 100747776 | 102947242 | <0.001 | OR7H2P,SLCO4C1,SLCO6A1,PAM,LOC134505,FLJ20125,HISPPD1,LOC643206,LOC90355,NUDT12 |
| 5q21.2 | 103244445 | 103879121 | <0.001 | LOC643286 |
| 5q21.3 | 104891193 | 105633638 | <0.001 | LOC643388 |
| 6p25.3 | 332533 | 2508576 | <0.001 | IRF4,EXOC2,LOC642335,HUS1B,LOC442150,LOC285768,LOC642728,FOXQ1,FOXF2,LOC642910,FOXC1,GMDS |
| 6p25.2 | 2510789 | 2838760 | 0.019 | C6orf195,LOC340156,WRNIP1,SERPINB1,MGC39372,SERPINB9 |
| 6p25.1 | 6407859 | 7033889 | 0.039 | FLJ33708,LOC643875,LY86,LOC643893 |
| 6q14.1 | 80776182 | 80777751 | 0.007 | TTK |
| 6q14.1 | 80893035 | 81063517 | 0.002 | BCKDHB |
| 6q14.1 | 81063766 | 81657074 | <0.001 | BCKDHB,LOC442232 |
| 6q14.1 | 82486525 | 82753597 | 0.001 | FAM46A,LOC643628 |
| 6q14.1 | 82785254 | 83052756 | 0.001 | IBTK |
| 6q14.1 | 83052940 | 87488097 | <0.001 | TPBG,C6orf157,KIAA1117,PGM3,RWDD2,ME1,PRSS35,SNAP91,LOC401268,C6orf159,CYB5R4,LOC643761,C6orf117,C6orf84,LOC442233,TBX18,LOC643851,LOC643858,LOC643870,NT5E,SNX14,SYNCRIP,LOC643883,C6orf160,LOC643906,LOC643916,LOC643926 |
| 6q14.3 | 87514569 | 92873588 | <0.001 | LOC643962,HTR1E,LOC643971,CGA,LOC442234,ZNF292,GJB7,LOC644016,C6orf162,C6orf163,C6orf165,TAF13P,SLC35A1,RARSL,ORC3L,C6orf166,SPACA1,CNR1,LOC644119,ACTBP8,RNGTT,CYCSP16,PNRC1,SRrp35,ACY1L2,GABRR1,GABRR2,UBE2J1,RRAGD,ANKRD6,DJ122O8.2,MDN1,CASP8AP2,LOC644269,CX62,BACH2,LOC653299,MAP3K7,LOC644479 |
| 6q15 | 93057270 | 93133481 | 0.003 | NA |
| 6q16.1 | 93135005 | 97220181 | <0.001 | LOC135270,EPHA7,LOC643432,LOC644562,LOC644569,LOC644579,CYCSP17,MANEA,LOC442236,FUT9,KIAA0776,FHL5,LOC442237 |
| 6q16.1 | 97221806 | 97224557 | 0.005 | LOC442237 |
| 6q16.1 | 97225042 | 97375336 | 0.002 | LOC442237,GPR63 |
| 6q16.1 | 97378667 | 97379068 | 0.001 | GPR63 |
| 6q16.1 | 97381595 | 97734258 | <0.001 | GPR63,LOC642420,C6orf66,KIAA1900,C6orf167 |
| 6q16.1 | 97739858 | 97740817 | 0.003 | C6orf167 |
| 6q16.1 | 97744252 | 97789203 | 0.01 | C6orf167 |
| 6q16.1 | 97794624 | 99205224 | <0.001 | C6orf167 |
| 6q16.1 | 99218420 | 100051064 | <0.001 | POU3F2,FBXL4,DHRS6P1,C6orf168,COQ3,C6orf111,USP45 |
| 6q16.2 | 100053339 | 100311390 | 0.032 | USP45,LOC642765,CCNC,PRDM13 |
| 6q16.2 | 100429891 | 101130678 | 0.003 | GPR145,LOC642491,LOC442238,LOC442239,SIM1,ASCC3,LOC153893 |
| 6q16.3 | 101131125 | 101149744 | 0.004 | ASCC3 |
| 6q16.3 | 101158222 | 101755257 | <0.001 | ASCC3,LOC653171 |
| 6q16.3 | 101844266 | 102422014 | 0.001 | GRIK2 |
| 6q16.3 | 104842863 | 109283510 | <0.001 | HACE1,LOC643075,LIN28B,BVES,POPDC3,PREP,LOC643132,PRDM1,ATG5,AIM1,RTN4IP1,QRSL1,LOC553137,C6orf203,KIAA1553,C6orf210,FLJ10159,SCML4,SEC63,LOC643297,LOC642741,OSTM1,NR2E1,SNX3,LACE1,FOXO3A,LOC442240,ARMC2 |
| 6q21 | 109335761 | 110583124 | 0.02 | ARMC2,ATP5J2P2,SESN1,C6orf182,LOC389422,FLJ37396,LOC442241,C6orf184,LOC442242,C6orf185,CD164,PPIL6,SMPD2,MICAL1,ZBTB24,FLJ25791,FLJ42177,C6orf199,KIAA0274,GPR6,WASF1 |
| 6q21 | 110592139 | 110592843 | 0.007 | WASF1 |
| 6q21 | 112585470 | 114335991 | 0.006 | LAMA4,LOC442247,LOC442249,LOC643859,LOC389424,LOC643884,LOC389425,LOC442251,MARCKS,FLJ34503 |
| 7q36.1 | 150963275 | 153288413 | 0.011 | PRKAG2,LOC644090,LOC644134,GALNTL5,GALNT11,LOC644227,MLL3,LOC155100,FABP5L3,LOC644271,LOC644309,XRCC2,LOC644333,ACTR3B,FLJ42291,LOC644571,LOC653748 |
| 8p23.3 | 103381 | 4153141 | <0.001 | OR4F21,LOC644102,LOC644113,LOC644128,ZNF596,LOC644147,FBXO25,C8orf42,LOC389607,ERICH1,C8orf68,LOC401442,LOC644319,LOC644327,LOC644421,DLGAP2,CLN8,C8orf61,ARHGEF10,LOC644668,KBTBD11,LOC644711,MYOM2,CSMD1 |
| 8p23.2 | 4185927 | 13293011 | <0.001 | CSMD1,LOC392179,LOC392180,MCPH1,ANGPT2,AGPAT5,XKR5,DEFB1,LOC392181,LOC645265,DEFA6,DEFA4,DEFA8P,LOC645285,DEFA1,LOC645303,LOC653600,LOC645316,DEFA3,LOC645336,DEFA5,LOC645344,LOC441320,OR7E125P,LOC645353,LOC349196,FAM90A3,LOC645362,FAM90A4,LOC645378,LOC441314,LOC653618,FAM90A5,LOC645391,LOC645392,FAM66E,LOC401447,LOC645402,LOC402329,LOC283202,LOC645413,HSPDP3,DEFB103A,SPAG11,DEFB104A,DEFB106A,DEFB105A,LOC441316,LOC645489,FAM90A6P,LOC645493,LOC645509,FAM90A7,LOC645525,LOC645535,LOC645550,LOC645541,LOC645558,LOC645567,LOC645572,OR7E157P,LOC645599,LOC645627,LOC645633,LOC645649,LOC645642,LOC645651,LOC645660,LOC441326,LOC645673,LOC441323,LOC645681,FAM90A8,LOC645689,LOC441325,LOC645701,LOC645709,LOC645718,FAM90A9,LOC645732,FAM90A10,LOC645743,LOC645750,LOC653666,LOC653667,LOC653669,LOC653423,LOC653671,LOC645808,DEFB4,LOC653425,LOC645826,LOC392187,LOC392188,LOC645836,LOC441340,FAM90A11P,LOC645863,FAM90A12,LOC645875,LOC645879,LOC645888,OR7E96P,LOC645906,LOC286042,DKFZp761P0423,CLDN23,MFHAS1,MRPS18CP2,LOC645960,THEX1,RNU7P4,PPP1R3B,LOC645986,TNKS,MSRA,LOC346702,UNQ9391,RP1L1,LOC203076,SOX7,PINX1,XKR6,C8orf15,C8orf16,LOC392193,MTMR9,AMAC1L2,TDH,C8orf13,BLK,GATA4,C8orf49,NEIL2,FDFT1,CTSB,OR7E158P,OR7E161P,DEFB137,DEFB136,DEFB134,LOC646244,LOC646253,OR7E160P,LOC646266,LOC440053,LOC392196,LOC392197,DUB3,FAM90A2P,LOC646287,LOC646290,LOC653726,FAM86B1,ZNF705CP,LOC653727,LOC646304,LOC389633,LOC646318,LOC646323,LOC653333,LOC646344,LOC653337,LOC646354,OR7E8P,OR7E15P,OR7E10P,LONRF1,FLJ36980,KIAA1456,DLC1 |
| 8p22 | 13297590 | 13300340 | 0.001 | DLC1 |
| 8p22 | 13304506 | 15704610 | <0.001 | DLC1,C8orf48,SGCZ,**TUSC3** |
| 8p22 | 15708398 | 16596237 | <0.001 | LOC137012,LOC646433,MSR1,LOC646440,MRPL49P2,LOC646444 |
| 8p22 | 16610888 | 17202048 | <0.001 | FGF20,EFHA2,ZDHHC2,CNOT7,VPS37A,MTMR7 |
| 8p22 | 17202356 | 17210656 | 0.003 | MTMR7 |
| 8p22 | 17211136 | 25040250 | <0.001 | MTMR7,LOC646479,SLC7A2,PDGFRL,MTUS1,FGL1,PCM1,ASAH1,MRPS18CP3,NAT1,AACP,LOC392206,NAT2,LOC653754,PSD3,LOC442382,SH2D4A,ChGn,C8orf35,LPL,SLC18A1,ATP6V1B2,LZTS1,RNU3P2,LOC646608,GFRA2,LOC653765,OR6R2P,DOK2,XPO7,NPM2,FGF17,EPB49,RAI16,NUDT18,HR,C8orf20,LGI3,SFTPC,BMP1,PHYHIP,LOC646654,POLR3D,PIWIL2,SLC39A14,PPP3CC,SORBS3,PDLIM2,C8orf58,KIAA1967,BIN3,FLJ14107,EGR3,PEBP4,RHOBTB2,TNFRSF10B,TNFRSF10C,TNFRSF10D,TNFRSF10A,LOC389641,CHMP7,R3HCC1,LOXL2,ENTPD4,LOC646708,SLC25A37,LOC653778,LOC646721,NKX3-1,NKX2-6,LOC646731,STC1,LOC646740,ADAM28,ADAMDEC1,ADAM7,NEF3,NEFL |
| 8p21.2 | 25047022 | 25047022 | 0.001 | NA |
| 8p21.2 | 25047045 | 32142834 | <0.001 | DOCK5,GNRH1,KCTD9,CDCA2,EBF2,PPP2R2A,LOC157489,BNIP3L,LOC440258,PNMA2,LOC338097,DPYSL2,ADRA1A,LOC646818,STMN4,TRIM35,PTK2B,CHRNA2,EPHX2,GULOP,CLU,SCARA3,LOC646843,CCDC25,ESCO2,PBK,SCARA5,HMFN0672,ELP3,LOC389644,PNOC,ZNF395,FBXO16,FZD3,LOC653787,EXTL3,RC74,FLJ21616,KIF13B,DUSP4,LOC646909,MAP2K1P1,TMEM66,LEPROTL1,DCTN6,LOC392209,LOC642319,LOC92755,RBPMS,LOC642356,GTF2E2,GSR,LOC642388,UBXD6,PPP2CB,TEX15,LOC441344,PURG,WRN,LOC642513,LOC653104 |
| 8p12 | 32418529 | 38389971 | <0.001 | NRG1,FUT10,RBM13,C8orf41,RNF122,DUSP26,LOC642685,VENTXP5,LOC388460,CYCSP3,LOC137107,LOC653122,UNC5D,LOC642855,MRPS7P1,FKSG2,LOC642879,LOC642950,ZNF703,SPFH2,PROSC,GPR124,BRF2,RAB11FIP1,GOT1L1,ADRB3,EIF4EBP1,ASH2L,STAR,LSM1,BAG4,DDHD2,PPAPDC1B,WHSC1L1,LETM2,FGFR1 |
| 8p11.22 | 38436403 | 38456390 | 0.01 | FGFR1 |
| 8p11.22 | 38460773 | 39079937 | <0.001 | FLJ43582,RNF5P1,LOC653218,TACC1,PLEKHA2,HTRA4,TM2D2,ADAM9 |
| 8p11.22 | 39080637 | 39365833 | 0.011 | ADAM9,ADAM32,ADAM5 |
| 8p11.22 | 39507457 | 39693018 | 0.012 | LOC643197,ADAM18 |
| 8p11.21 | 39703325 | 40302955 | 0.001 | ADAM18,ADAM2,INDO,LOC169355,C8orf4 |
| 8p11.21 | 40312067 | 42226263 | <0.001 | ZMAT4,SFRP1,GOLGA7,LOC392214,SLD5,AGPAT6,FLJ25169,ANK1,LOC643484,MYST3,AP3M2,PLAT |
| 8p11.21 | 42229343 | 42278211 | 0.026 | IKBKB |
| 8p11.21 | 42278323 | 42386034 | <0.001 | IKBKB,POLB,DKK4,VDAC3 |
| 10q23.31 | 89669027 | 89688352 | 0.009 | **PTEN** |
| 10q23.31 | 89716234 | 89720628 | 0.021 | PTEN |
| 10q23.31 | 89725578 | 90330693 | 0.008 | C10orf59 |
| 10q23.31 | 90338800 | 90347139 | 0.026 | LIPL1 |
| 11q23.2 | 114417286 | 117124558 | <0.001 | IGSF4,LOC441623,LOC644842,LOC283143,LOC440069,MGC13125,ZNF259,APOA5,APOA4,APOC3,APOA1,KIAA0999,LOC645044,LOC653303,PAFAH1B2,SIDT2,TAGLN,PCSK7,DKFZp547C195,BACE1,CEP164,DSCAML1 |
| 12p13.31 | 6152117 | 7226252 | 0.019 | CD9,LOC653324,PLEKHG6,TNFRSF1A,SCNN1A,LTBR,LOC390283,LOC390284,TNFRSF7,TAPBPL,VAMP1,PKP2P1,MRPL51,CNAP1,GAPDH,HOM-TES-103,NOL1,CHD4,GPR92,ACRBP,ING4,ZNF384,DKFZp547D2210,COPS7A,MLF2,PTMS,LAG3,CD4,GPR162,LEPREL2,GNB3,CDCA3,USP5,TPI1,SPSB2,LOC283345,B7,ENO2,ATN1,GRCC10,PTPN6,PHB2,EMG1,C3F,LOC390285,C1S,LOC643676,LOC653342,C1R,C1RL,RBP5,CLSTN3 |
| 12p13.31 | 9521824 | 9525138 | 0.027 | LOC644125 |
| 12p13.31 | 9529177 | 9604942 | <0.001 | LOC644125 |
| 12p13.2 | 10474635 | 10487384 | 0.008 | KLRC2 |
| 12p13.2 | 10865315 | 10869669 | 0.033 | TAS2R10 |
| 12p13.2 | 11434674 | 15648173 | <0.001 | LOC653247,LOC440084,LOC644346,LOC644359,LOC644375,ETV6,BCL2L14,LOC643287,LRP6,MORF4LP4,MANSC1,LOH12CR1,DUSP16,LOC644467,CREBL2,GPR19,CDKN1B,DKFZP434F0318,DDX47,LOC440086,LOC387841,GPRC5A,GPRC5D,HEBP1,KIAA1467,GSG1,LOC644574,EMP1,FLJ33810,GRIN2B,LOC644693,ATF7IP,FLJ22662,GUCY2C,HIST4H4,H2AFJ,WBP11,MGC47869,LOC440087,ART4,MGP,FLJ32115,ARHGDIB,PDE6H,RERG,PTPRO |
| 13q12.3 | 31851388 | 32648181 | <0.001 | BRCA2,IFIT1P,CG018,LOC88523,PFAAP5,APRIN,LOC122038,KL,STARD13 |
| 13q13.1 | 32648293 | 32652744 | 0.033 | STARD13 |
| 13q13.1 | 32652973 | 32774437 | 0.017 | STARD13 |
| 13q13.1 | 33048828 | 33707212 | 0.005 | RFC3 |
| 13q13.1 | 33765548 | 34535203 | 0.008 | NBEA |
| 13q13.3 | 37768286 | 39552176 | 0.003 | UFM1,LOC646918,FREM2,LOC646929,STOML3,LOC646933,C13orf23,LOC387921,LOC646937,LOC646944,LHFP,COG6,LOC646953 |
| 13q13.3 | 39760155 | 53172163 | <0.001 | LOC646982,LOC646990,FOXO1A,MRPS31,SLC25A15,CYCSP34,LOC643729,ELF1,WBP4,KBTBD6,CALM2P3,KBTBD7,MTRF1,RAC1P3,NARG1L,LOC647000,OR7E36P,OR7E155P,LOC647007,RGC32,KIAA0564,DGKH,MAPK6PS3,LOC341651,LOC647031,AKAP11,FABP3P2,TNFSF11,FLJ40919,EPSTI1,DNAJC15,PIG38,LOC647049,LOC647050,FLJ31846,FLJ38725,LOC400126,MGC5590,C13orf21,TSC22D1,LOC400129,LOC144817,LOC647075,NUFIP1,KIAA1704,GTF2F2,KCTD4,TPT1,LOC644007,SLC25A30,LOC647080,LOC647085,COG3,FLJ32682,LOC390402,NURIT,LOC283514,LOC647092,KIAA0853,CPB2,LCP1,LOC220416,LOC390403,C13orf18,LOC653817,OR7E101P,LOC81993,LRCH1,ESD,HTR2A,LOC647113,SUCLA2,NUDT15,MED4,ITM2B,RB1,P2RY5,RCBTB2,LOC644250,CYSLTR2,LOC338099,LOC647131,FNDC3A,RAD17P2,LOC387924,MLNR,CDADC1,CAB39L,SETDB2,PHF11,RCBTB1,ARL11,EBPL,KPNA3,LOC220429,C13orf1,RFP2OS,RFP2,KCNRG,DLEU2,DLEU1,FLJ31945,FAM10A4,LOC647154,DLEU7,FLJ11712,GUCY1B2,LOC647166,LOC341674,FLJ30707,LOC647174,DDX26,LOC220433,LOC647181,WDFY2,FLJ13639,FLJ37307,DKFZP434K1172,LOC400135,ATP7B,LOC440138,UTP14C,LOC387927,FKSG49,NEK3,THSD1P,LOC644623,THSD1,VPS36,CKAP2,LOC220115,LOC653821,LOC144983,SUGT1,LECT1,LOC121981,PCDH8,OLFM4,LOC647207 |
| 13q14.3 | 53275683 | 58264530 | <0.001 | LOC647213,LOC387930,LOC647221,LOC647226,FLJ40296,LOC653823,LOC653824,LOC653825,LOC653826,LOC647245,PCDH17,LOC647248,LOC387931,LOC341689 |
| 13q21.1 | 58270722 | 59469992 | <0.001 | DIAPH3 |
| 13q21.1 | 59480757 | 59523908 | 0.001 | DIAPH3 |
| 13q21.1 | 59524639 | 64285473 | <0.001 | LOC440142,TDRD3,LOC390407,LOC647256,LOC647257,PCDH20,LOC647258,LOC647259,LOC647260,LOC647261,OR7E156P,LOC647262,LOC401737,LOC647264,OR7E104P,LOC647266,LOC647267 |
| 13q21.31 | 64293200 | 64464084 | 0.007 | LGMN2P |
| 13q21.31 | 64481734 | 64956089 | <0.001 | STARP1,LOC647269 |
| 13q21.31 | 65199390 | 65262511 | 0.012 | LOC387933 |
| 13q21.31 | 65277462 | 65321594 | 0.014 | LOC647270 |
| 13q21.32 | 66386763 | 67401818 | <0.001 | PCDH9,LOC400141,LOC647271,LOC647272,LOC390411,OR7E111P,OR7E33P |
| 13q21.32 | 68168627 | 73919898 | <0.001 | KLHL1,LOC647277,DACH1,LOC440145,FLJ22624,KIAA1008,C13orf24,LOC338091,KLF5,LOC387934,LOC647279,LOC647281,KLF12,LOC400145,LOC647283 |
| 13q22.1 | 73922614 | 74507207 | <0.001 | LOC122145 |
| 13q22.1 | 74640162 | 75565887 | <0.001 | LOC647288,TBC1D4,COMMD6,UCHL3,LMO7 |
| 13q22.2 | 76484426 | 76704181 | 0.017 | FBXL3,MYCBP2 |
| 13q22.2 | 76707255 | 77361850 | 0.049 | MYCBP2,SCEL,LOC647295,FLJ30046 |
| 14q24.3 | 73852268 | 74312883 | 0.001 | C14orf115,RPS2P2,LOC646658,NPC2,RAP1AP,HBLD1,LTBP2,KIAA0317,C14orf111,YLPM1 |
| 16q12.1 | 49709835 | 50311459 | <0.001 | SALL1,UNGP1,LOC642659 |
| 16q12.1 | 50316651 | 50656309 | 0.005 | LOC388276 |
| 16q12.1 | 50659056 | 59861627 | <0.001 | LOC388276,TNRC9,LOC643714,LOC146253,LOC643748,LOC390730,CHD9,LOC441770,LOC643802,RBL2,FTS,KIAA1005,FTO,IRX3,LOC643911,LOC388279,IRX5,IRX6,MMP2,AYTL1,CAPNS2,SLC6A2,LOC390732,CES4,CES1,CES7,GNAO1,AMFR,NUDT21,FLJ10826,BBS2,MT4,MT3,MT2A,MT1L,MT1E,MT1M,MT1J,MT1A,MTM,LOC441771,MT1B,MT1F,MT1G,MT1H,LOC644314,MT1X,NUP93,SLC12A3,HERPUD1,CETP,NOD27,LOC644349,CPNE2,NIP30,KIAA1972,ARL2BP,PLLP,CCL22,CX3CL1,CCL17,CIAPIN1,COQ9,POLR2C,DOK4,MGC10992,GPR114,LOC255519,GPR56,GPR97,C16orf50,KATNB1,KIFC3,LOC388282,CNGB1,TEPP,ZNF319,FLJ13154,MMP15,GTL3,CSNK2A2,LOC644544,HSPC065,Klkbl4,FLJ13912,NDRG4,FLJ21148,CNOT1,FLJ10815,GOT2,LOC644600,LOC644649,LOC388283,LOC644702,LOC643358 |
| 16q21 | 59861689 | 59863206 | 0.007 | NA |
| 16q21 | 59864559 | 69396839 | <0.001 | CDH8,LOC390735,CDH11,LOC283867,CDH5,BEAN,TK2,CKLF,CMTM1,CMTM2,CMTM3,CMTM4,DYNC1LI2,FLJ35894,APPBP1,CA7,LOC644978,PDP2,CDH16,RRAD,FAM96B,CES2,FLJ21736,FLJ37464,CBFB,LIN10,MGC4655,TRADD,FBXL8,HSF4,NOL3,LOC653319,LOC283849,E2F4,ELMO3,LRRC29,LOC653563,HSPC171,FHOD1,SLC9A5,PLEKHG4,KCTD19,LRRC36,CGI-38,ZDHHC1,HSD11B2,ATP6V0D1,AGRP,FAM65A,CTCF,RLTPR,ACD,PARD6A,C16orf48,LOC388284,MGC11335,RANBP10,TSNAXIP1,C16orf56,THAP11,NUTF2,LOC645138,RCD-8,UNQ2446,PSKH1,CTRL,PSMB10,LCAT,SLC12A4,DPEP3,DPEP2,DDX28,DUS2L,NFATC3,RBM35B,LYPLA3,SLC7A6,SLC7A6OS,PRMT7,SMPD3,LOC643895,ZFP90,CDH3,CDH1,LOC645198,FLJ12688,FLJ12331,HAS3,DERPC,CIRH1A,SNTB2,VPS4A,COG8,PDF,NIP7,TMED6,TERF2,CYB5-M,NFAT5,NQO1,NOB1P,LOC644035,WWP2,LOC348174,LOC645282,LOC645287,LOC283970,LOC440349,LOC645299,LOC645307,PDPR,MGC34761,LOC645325,EXOSC6,AARS,DDX19B,DDX19A,DDX19-DDX19L,ST3GAL2,LOC645346,FUK,COG4,SF3B3,MGC34647,LOC92154,VAC14 |
| 16q22.1 | 69762696 | 69776096 | 0.001 | HYDIN |
| 16q22.1 | 69776974 | 88815037 | <0.001 | HYDIN,FLJ11171,CALB2,LOC390738,ZNF23,LOC645396,ZNF19,CHST4,TAT,MARVELD3,PHLPPL,AP1G1,BOAT,LOC146517,LOC55565,KIAA0174,PKD1L3,LOC645443,DHODH,HP,HPR,TXNL4B,DHX38,PMFBP1,LOC390739,LOC645478,LOC342374,**ATBF1**,C16orf47,LOC441506,LOC645656,LOC401859,PSMD7,LOC440386,LOC440348,LOC441773,LOC497190,GLG1,LOC645713,RFWD3,LOC645726,MLKL,FA2H,WDR59,ZNRF1,LDHD,ZFP1,LOC441774,CTRB2,CTRB1,BCAR1,CFDP1,LOC124491,CHST6,LOC645799,CHST5,GABARAPL2,ADAT1,KARS,TERF2IP,LOC645821,LOC401860,LOC124496,LOC401861,LOC441775,CNTNAP4,LOC645873,MON1B,ADAMTS18,LOC645919,KIAA1576,CLEC3A,LOC342419,WWOX,LOC645947,LOC645957,MAF,LOC440389,DYNLRB2,CDYL2,DC13,BM039,ASCIZ,C16orf46,GCSH,PKD1L2,BCMO1,GAN,CMIP,PLCG2,HSPC105,HSD17B2,MPHOSPH6,CDH13,HSBP1,MLYCD,OKL38,EFCBP2,LOC146167,MBTPS1,HSDL1,LRRC50,TAF1C,LOC161931,KCNG4,WFDC1,KIAA0703,KIAA1609,COTL1,C16orf44,USP10,CRISPLD2,LOC123862,ZDHHC7,KIAA0513,FAM92B,LOC123855,MGC22001,KIAA0182,Pfs2,MGC17624,COX4NB,LOC646365,COX4I1,LOC401863,IRF8,DKFZp434O0320,LOC401864,FOXF1,LOC401865,FLJ12998,FLJ30679,FOXC2,FOXL1,FBXO31,MAP1LC3B,ZCCHC14,JPH3,KLHDC4,SLC7A5,CA5A,BANP,LOC646638,ZNF469,ZFPM1,FLJ45530,NHN1,IL17C,CYBA,MVD,SNAI3,RNF166,LOC348180,FAM38A,FLJ45121,FLJ40448,CDT1,APRT,GALNS,HSPC176,LOC390748,CBFA2T3,LOC642452,LOC197322,FLJ36701,CDH15,LOC146429,LOC642377,FLJ31875,LOC642533,ANKRD11,SPG7,RPL13,HBII-202,CPNE7,DPEP1,PCOLN3,C16orf55,CDK10,MGC26885,C16orf7,ZFP276,FANCA,SPIRE2,NULP1,MC1R,TUBB3,FLJ20186,MGC16385,AFG3L1,MGC3101,GAS8,C16orf3,PRDM7,LOC197331,LOC653138,LOC642780,LOC642788,LOC642715 |
| 17p13.3 | 527 | 2064746 | <0.001 | LOC653051,RPH3AL,LOC400566,MGC45871,VPS53,FAM57A,GEMIN4,LOC642370,LOC642378,C17orf25,RNMTL1,NXN,TIMM22,ABR,MRPL14P1,TUSC5,YWHAE,CRK,MYO1C,SKIP,PITPNA,SLC43A2,RILP,SCARF1,PRPF8,MGC14376,WDR81,SERPINF2,SERPINF1,SMYD4,RPA1,LOC642502,RTN4RL1,DPH1,OVCA2,LOC642670,HIC1,C17orf31 |
| 17p13.3 | 2064761 | 2065365 | <0.001 | C17orf31 |
| 17p13.3 | 2066568 | 2192515 | 0.002 | C17orf31,LOC653034,SRR,LOC440396,TSR1,RUTBC1 |
| 17p13.3 | 2192615 | 2192666 | 0.001 | RUTBC1 |
| 17p13.3 | 2193109 | 2258380 | 0.003 | RUTBC1,MNT,LOC284009 |
| 17p13.3 | 2259105 | 2265300 | 0.002 | LOC284009 |
| 17p13.3 | 2265461 | 2286836 | 0.011 | LOC284009,METT10D |
| 17p13.3 | 2291521 | 3082528 | 0.01 | METT10D,LOC642746,PAFAH1B1,KIAA0664,LOC642801,GARNL4,LOC653127,OR1D4,OR1D2,OR1E3P,OR1G1,OR1P1P,OR1A2,OR1A1 |
| 17p13.3 | 3082586 | 3083042 | 0.001 | NA |
| 17p13.3 | 3291835 | 3296925 | <0.001 | SPATA22 |
| 17p13.3 | 3297306 | 4175753 | 0.003 | SPATA22,ASPA,TRPV3,TRPV1,CARKL,CTNS,TAX1BP3,TMEM93,P2RX5,ITGAE,GSG2,HSA277841,LOC653191,CAMKK1,P2RX1,ATP2A3,ZZEF1,CYB5D2,ANKFY1,UBE2G1 |
| 17p13.2 | 4180021 | 13956377 | <0.001 | UBE2G1,MGC29671,MYBBP1A,GGT6,LOC124974,FLJ42461,ALOX15,PELP1,ARRB2,MGC88387,CXCL16,LOC643165,ZMYND15,TM4SF5,VMO1,LOC388323,PSMB6,PLD2,MINK1,CHRNE,GP1BA,SLC25A11,RNF167,PFN1,ENO3,SPAG7,CAMTA2,INCA1,KIF1C,GPR172B,ZFP3,ZNF232,LOC643247,USP6,ZNF594,UNQ5783,RABEP1,NUP88,RIP,C1QBP,DHX33,DERL2,MIS12,NALP1,LOC643333,LOC643340,KIAA0523,AIPL1,FAM64A,PITPNM3,KIAA0753,LOC643501,TXNL5,MED31,LOC342531,SLC13A5,BIRC4BP,FBXO39,TEKT1,ALOX12P2,ALOX12,MGC71993,C17orf49,BCL6B,SLC16A13,SLC16A11,CLEC10A,ASGR2,ASGR1,DLG4,ACADVL,DVL2,PHF23,GABARAP,DULLARD,DERP6,CLDN7,SLC2A4,YBX2,EIF5A,GPS2,KIAA1787,LOC390760,CENTB1,KCTD11,TMEM95,TNK1,PLSCR3,C17orf61,NLGN2,LOC374768,C17orf74,TMEM102,FGF11,CHRNB1,ZBTB4,LOC643664,POLR2A,TNFSF12,TNFSF12-TNFSF13,TNFSF13,SENP3,EIF4A1,CD68,MPDU1,SOX15,FXR2,SAT2,SHBG,ATP1B2,**TP53**,WDR79,EFNB3,DNHD3,JMJD3,TMEM88,LSMD1,CYB5D1,CHD3,LOC284023,KCNAB3,TRAPPC1,LIP8,GUCY2D,ALOX15B,ALOX12B,ALOXE3,TRR1,HES7,PER1,VAMP2,TMEM107,C17orf59,AURKB,C17orf44,C17orf68,PFAS,SLC25A35,RANGNRF,ARHGEF15,ODF4,LOC643891,LOC124751,RPL26,LOC643904,NDEL1,MYH10,LOC643933,CCDC42,LOC388333,FLJ35773,C17orf38,PIK3R5,NTN1,STX8,WDR16,USP43,DHRS7C,LOC644070,GLP2R,RCV1,GAS7,LOC644008,RPS27AP1,MYH13,MYH8,MYH4,MYH1,MYH2,MYH3,SCO1,C17orf48,LOC388335,LOC644139,FLJ45455,DNAH9,ZNF18,MAP2K4,FLJ34690,MYOCD,KIAA0672,ELAC2,HS3ST3A1,LOC644361,LOC653475,COX10 |
| 17p12 | 13956530 | 13968181 | 0.002 | COX10 |
| 17p12 | 13969167 | 16663527 | <0.001 | COX10,CDRT15,HS3ST3B1,LOC388339,FLJ45831,LOC440402,LOC441781,PMP22,TEKT3,CDRT4,LOC653497,FAM18B2,LOC653500,CDRT1,TRIM16,ZNF286,UBE2SP1,MGC51025,LOC644694,LOC644703,IL6STP,MEIS3P1,LOC644720,ADORA2B,TTC19,NCOR1,PIGL,PRR6,UBB,TRPV2,C17orf45,FLJ35696,ZNF287,LOC644422,ZNF624,RNASEH1P2,LOC147226,LOC252841,LOC266619,LOC353194 |
| 17p11.2 | 16664774 | 17091866 | 0.008 | LOC353194,LOC400578,LOC147228,LOC339186,CLPSMCR,LOC96597,TNFRSF13B,M-RIP,LOC201164,FLCN,LOC653540,LOC441783,COPS3 |
| 17p11.2 | 17092663 | 18191252 | <0.001 | COPS3,NT5M,LOC388344,LOC644909,MED9,RASD1,PEMT,RAI1,LOC644931,SREBF1,TOM1L2,LRRC48,ATPAF2,C17orf39,DRG2,MYO15A,OFOXD,LLGL1,FLII,SMCR7,TOP3A,SMCR8,SHMT1 |
| 17p11.2 | 18191787 | 18274640 | 0.028 | SHMT1,LOC645023,LOC645027,FLJ35934,LOC339240,LOC339241 |
| 17p11.2 | 18438029 | 19707618 | <0.001 | FLJ36492,FLJ40244,LOC645139,LOC645143,FOXO3B,LOC653524,FBXW10,FAM18B,LOC645151,LOC653577,PRPSAP2,SLC5A10,LOC644819,LOC644815,GRAP,LOC400581,LOC388436,EPN2,EPPB9,MAPK7,MFAP4,ZNF179,LOC125208,FLJ10847,LOC645221,ALDH3A2,FLJ31196,ALDH3A1,LOC441784,ULK2 |
| 17p11.2 | 19708360 | 21485816 | 0.003 | ULK2,AKAP10,LOC645255,SPECC1,KIAA0565,LOC147150,LOC347717,FAM106B,LOC347716,LOC339256,LOC284194,LOC339257,LOC353196,LOC644940,LOC644945,LOC284196,LOC339258,COTL1P2,LOC256223,MEIS3P2,LOC645288,LOC645294,LOC645300,LOC645313,LOC644988,MGC87631,RNASEH1P1,LOC645350,USP22,LOC645364,DHRS7B,TMEM11,MGC33894,MAP2K3,LOC645427,KCNJ12,LOC440417,LOC645454,C17orf51,LOC645479 |
| 20p13 | 9583 | 26253492 | <0.001 | DEFB125,DEFB126,DEFB127,LOC642293,DEFB129,LOC642315,DEFB32,C20orf96,ZCCHC3,SOX12,C20orf98,TRIB3,C20orf18,TBC1D20,CSNK2A1,TCF15,SRXN1,SCRT2,C20orf54,C20orf55,RPS10L,ANGPT4,RSPO4,PSMF1,ACTGP3,C20orf46,LOC400831,LOC642636,SNPH,SDCBP2,LOC642671,FKBP1A,NSFL1C,PTPNS1L3,LOC642697,PTPNS1L2,SIRPB1,SIRPB2,LOC441938,PTPNS1,PDYN,RPL7P2,STK35,LOC388780,TGM3,TGM6,SNRPB,ZNF343,TMC2,NOL5A,IDH3B,KIAA1442,RPL19P1,CPXM,C20orf141,LOC389286,C20orf81,VPS16,PTPRA,GNRH2,MRPS26,OXT,AVP,LOC642991,UBOX5,FLJ13149,ProSAPiP1,C20orf116,ITPA,SLC4A11,UBE2V1P1,ATRN,SF3A3P,LOC643059,GFRA4,ADAM33,SN,HSPA12B,C20orf27,C20orf28,CENPB,CDC25B,C20orf29,VISA,PANK2,LOC653211,RNF24,FTLL1,RPL21P2,SMOX,ADRA1D,RPL7AL2,RPS4L2,PRNP,PRND,PRNT,RASSF2,LOC643305,SLC23A2,C20orf30,PCNA,CDS2,GPR73L1,LOC643406,LOC149837,RPS18P1,KIAA1434,EIF4EP1,FLJ25067,CHGB,LOC391230,CGI-09,MCM8,C20orf155,C20orf75,C20orf42,LOC643503,LOC149844,BMP2,pFUSIP1,HAO1,TXNDC13,PHKBP1,PLCB1,LOC643231,PLCB4,C20orf103,PAK7,ANKRD5,SNAP25,RPL23AP6,MKKS,C20orf94,JAG1,FAT1P1,RPS11P1,LOC441940,PGAM3P,BTBD3,PA2G4P2,C20orf38,C20orf82,TASP1,GAPDHP2,MRPS36P6,C20orf6,C20orf7,C20orf50,LOC643914,C20orf133,RPS3P1,SCYE1P,FLRT3,RNF11B,RPS10P2,ENSAP,LOC613266,PPIAP17,C20orf23,RPLP0P1,RPL7AL3,SNRPB2,OTOR,PCSK2,BFSP1,RPS27AP2,DSTN,RRBP1,C20orf179,SNX5,C20orf72,PTMAP3,OVOL2,RPL15P1,CSRP2BP,LOC644140,LOC653424,ZNF133,MGC44328,C20orf12,POLR3F,RBBP9,RPS19P1,SEC23B,LOC388789,HARS2,DUXAP7,LOC402011,HSPC072,C20orf79,SLC24A3,LOC644298,RPL12L3,RIN2,NAT5,CRNKL1,C20orf26,LOC643659,RPL17P1,INSM1,C20orf74,EIF4E2P1,MRPS11P1,C20orf19,RPS15AP1,XRN2,LOC644524,GSTM3P,NKX2-2,PAX1,SLC25A6P1,RPL41P1,ST13P,C20orf56,FOXA2,KRT18P3,CYB5P4,SSTR4,THBD,C1QR1,LOC200261,NXT1,ZNF336,NAPB,CSTL1,CST11,CST8,LOC164380,LOC128820,CST9L,CST9,CST3,CST4,CST1,CSTP2,CST2,CST5,CSTP1,LOC645076,GGTLA4,C20orf39,CST7,C20orf3,ACSS1,VSX1,LOC391239,ENTPD6,LOC653605,PYGB,C20orf22,PPIAP2,PSF1,KIAA0980,HDHD4,ZNF337,LOC645356,LOC388358,C20orf189,C20orf91,C20orf191,LOC645461,LOC284801 |
| 20q13.33 | 60448248 | 60569397 | <0.001 | GATA5,FLJ30313,C20orf166 |
| 20q13.33 | 61630925 | 61690093 | <0.001 | PTK6,SRMS,C20orf195,PRIC285,GMEB2 |
| 21q22.13 | 38804196 | 41794254 | <0.001 | **ERG,**C21orf24,ETS2,FLJ45139,LOC391282,PCBP2P1,DSCR2,BRWD1,C21orf87,HMGN1,WRB,C21orf13,SH3BGR,C21orf88,B3GALT5,LOC150084,PCP4,DSCAM,LOC645279,BACE2,PLAC4,FAM3B,MX2,MX1,TMPRSS2 |
| 21q22.2 | 41794281 | 41794281 | 0.01 | **TMPRSS2** |
| 22q13.2 | 43869623 | 45940645 | <0.001 | NUP50,C22orf9,UPK3A,C22orf8,SMC1L2,RIBC2,FBLN1,ATXN10,WNT7B,FLJ10945,LOC642648,FLJ27365,LOC642736,PPARA,LOC653142,LOC150383,PKDREJ,FLJ20699,GTSE1,TRMU,CELSR1,DIP,CERK,TBC1D22A,LOC642757,LOC642769 |
