# Supplementary material for: Genome-wide identification of significant aberrations in cancer genome
Source: BMC Genomics. 2012 Jul 27;13:342. doi: 10.1186/1471-2164-13-342 (PMC3428679; doi:10.1186/1471-2164-13-342)
Supplement: Additional file 4 — Table 6 and Suplementary Table 7. Details about the implicated SCAs and full list of genes covered by these SCAs, derived from the lung adenocarcinoma data set. [file 1471-2164-13-342-S4.doc]

**Supplementary Table 6.** Details about the implicated SCAs and full list of genes covered by these SCAs, derived from genome-wide analysis of the lung adenocarcinoma data set. (Start: Start point of SCAs. End: End point of SCAs.) (Based on hg18 human genome assembly)

| **Cyto-**  **band** | **Region** | | | **P-value** | | **Genes covered by the SCA regions** | |
| --- | --- | --- | --- | --- | --- | --- | --- |
|  | **Start** | **End** | |  |  |  |  |
| ***Amplification*** | | | | | | | |
| 1p31.1 | 77219051 | 77219148 | | 0.002 | | ST6GALNAC5 | |
| 1q32.3 | 212361064 | 212361947 | | <0.001 | | NA | |
| 5p15.33 | 208367 | 3150468 | | <0.001 | | KIAA1909,LOC389257,LOC133957,SDHA,PDCD6,AHRR,LOC116349,EXOC3,SLC9A3,CEP72,TPPP,LOC653082,LOC643702,LOC643740,ZDHHC11,LOC653350,BRD9,TRIP13,NKD2,SLC12A7,SLC6A19,SLC6A18,**TERT**,CRR9,SLC6A3,AYTL2,LOC653102,LOC642535,LOC653378,LOC442128,MRPL36,NDUFS6,LOC389267,LOC644065,IRX4,IRX2,CEI | |
| 5p15.33 | 3555198 | 4333629 | | 0.005 | | LOC285577,IRX1 | |
| 5p15.31 | 6471689 | 12445539 | | <0.001 | | FLJ25076,NSUN2,SRD5A1,POLS,LOC645463,LOC645451,LOC442132,ADCY2,LOC134121,MGC5297,MTRR,LOC645502,LOC645583,LOC645607,SEMA5A,TAS2R1,LOC134145,CCT5,LOC134147,MARCH6,ROPN1L,LOC645735,LOC345711,LOC645763,DAP,**CTNND2** | |
| 5p15.2 | 12517763 | 15344398 | | <0.001 | | LOC645817,LOC391738,DNAH5,TRIO,FAM105A,LOC645894,LOC391739,EEF1AL11,FAM105B,ANKH,LOC642954 | |
| 5p15.1 | 17444228 | 20846434 | | 0.028 | | LOC285697,LOC340096,LOC646032,LOC391742,LOC391745,LOC391746,LOC646066,LOC391747,LOC402199,LOC391749,LOC285563,LOC402200,LOC402201,LOC646103,LOC402203,LOC202201,LOC402205,LOC646126,LOC402207,LOC402208,LOC646152,LOC646165,LOC391761,LOC646188,LOC391763,LOC391764,LOC391765,LOC391766,LOC391767,LOC391768,LOC391769,LOC391770,LOC646241,LOC646273,CDH18,LOC646280,LOC266786,LOC646296 | |
| 7p21.2 | 14380867 | 19017513 | | 0.001 | | DGKB,FLJ16237,MEOX2,LOC442511,SOSTDC1,LOC442512,LOC317727,ANKMY2,BZW2,TSPAN13,AGR2,LOC646252,BCMP11,RAD17P1,LOC646265,LOC653724,AHR,SNX13,LOC442285,LOC442658,PRPS1L1,LOC646302,HDAC9,NPM1P13 | |
| 7p21.1 | 19161066 | 19980905 | | 0.024 | | TWISTNB,MGC42090 | |
| 7p21.1 | 20370169 | 21247850 | | 0.032 | | ITGB8,LOC646385,ABCB5,SP8,LOC222901,LOC340274 | |
| 7p15.3 | 22314303 | 32358683 | | <0.001 | | LOC646468,LOC221838,IL6,RPS26e,TOMM7,LOC392872,DRCTNNB1A,KLHL7,NUPL2,GPNMB,C7orf30,IMP-3,MGC27348,TRA2A,CLK2P,LOC646524,LOC90693,MGC72075,STK31,NPY,MPP6,DFNA5,OSBPL3,CYCS,C7orf31,LOC392005,C7orf9,LOC646588,UBA52P1,NFE2L3,HNRPA2B1,CBX3,LOC442659,SNX10,LOC441204,LOC441205,LOC285941,SCAP2,LOC442660,LOC442661,LOC402643,LOC442290,HOXA1,HOXA2,HOXA3,LOC643903,HOXA4,HOXA5,HOXA6,HOXA7,HOXA9,HOXA10,HOXA11,LOC646692,HOXA13,EVX1,LOC392008,LOC401316,LOC442663,LOC442664,HIBADH,LOC646726,TAX1BP1,JAZF1,LOC402644,CREB5,LOC401317,KIAA0644,CPVL,LOC644086,CHN2,NANOGP4,LOC646745,LOC222171,LOC646762,LOC646767,DPY19L2P3,LOC644150,SCRN1,FKBP14,PLEKHA8,Ells1,ZNRF2,DKFZp586I1420,CARD4,C7orf24,LOC401320,GARS,CRHR2,INMT,FLJ22374,AQP1,GHRHR,ADCYAP1R1,NEUROD6,LOC223075,C7orf16,PDE1C | |
| 7p12.1 | 50629718 | 52385353 | | 0.002 | | GRB10,COBL,LOC642663 | |
| 7p12.1 | 52395032 | 52492660 | | 0.003 | | NA | |
| 7p12.1 | 52496765 | 53748861 | | 0.007 | | LOC642878,LOC392027,DKFZp564N2472,LOC653175,FLJ45974 | |
| 7p11.2 | 53796372 | 56118914 | | <0.001 | | FLJ45974,LOC222005,MGC33530,LOC392030,SEC61G,LOC643168,**EGFR**,CALM1P2,LANCL2,ECOP,LOC643336,LOC442308,LOC643348,FKBP9L,LOC653281,LOC643357,LOC643362,PSPHL,FLJ44060,LOC643399,FLJ39963,MRPS17,GBAS,PSPH,CCT6A,SUMF2,PHKG1 | |
| 7p11.2 | 56325172 | 57336046 | | 0.003 | | LOC441228,LOC154937,LOC643497,LOC643504,LOC254027,LOC346296,LOC643546,LOC401357,LOC643574,LOC643595,LOC402508,LOC340221,LOC402509,LOC643620,ZNF479,LOC643629,LOC643632 | |
| 8q21.13 | 80482123 | 81501697 | | 0.002 | | STMN2,HEY1,MRPS28,TPD52,LOC389671,LOC402342,LOC340443 | |
| 8q21.13 | 81573890 | 81715677 | | <0.001 | | ZBTB10,LOC389672,LOC646437,ZNF704 | |
| 8q21.13 | 81790560 | 82281412 | | 0.004 | | ZNF704,PAG1,LOC653745,LOC653746 | |
| 8q24.13 | 124498576 | 124746924 | | 0.002 | | C8orf32,FBXO32,C8ORFK36 | |
| 8q24.21 | 128055665 | 128283681 | | 0.012 | | SRRM1L | |
| 8q24.21 | 128350890 | 128351093 | | <0.001 | | NA | |
| 8q24.21 | 128351336 | 128352364 | | 0.003 | | NA | |
| 8q24.21 | 128415540 | 131910999 | | <0.001 | | POU5F1P1,**MYC**,PVT1,LOC441378,CCDC26,MLZE,FAM49B,DDEF1,ADCY8 | |
| 11q13.3 | 68457277 | 68521012 | | 0.046 | | IGHMBP2,MRGPRD | |
| 11q13.3 | 68561382 | 69172454 | | <0.001 | | TPCN2,MYEOV,LOC390218,**CCND1** | |
| 12p12.1 | 24055630 | 24766389 | | 0.028 | | SOX5,FLJ32894 | |
| 12p12.1 | 24803508 | 26119126 | | <0.001 | | BCAT1,LOC441630,LOC645167,LOC645177,LOC645186,LRMP,CASC1,LOC144363,**KRAS**,FLJ36004,LOC645233,RASSF8 | |
| 12p12.1 | 26151126 | 26176273 | | 0.04 | | BHLHB3 | |
| 12q13.2 | 56207455 | 56520529 | | <0.001 | | MBD6,DCTN2,KIF5A,PIP5K2C,DTX3,GEFT,SLC26A10,B4GALNT1,LOC441641,OS9,CENTG1,TSPAN31,**CDK4**,MARCH9,CYP27B1,METTL1,DKFZP586D0919,TSFM,AVIL,CTDSP2 | |
| 12q13.2 | 56542277 | 57235376 | | 0.008 | | XRCC6BP1,LOC338805 | |
| 12q14.1 | 61896783 | 61896809 | | 0.013 | | NA | |
| 12q14.1 | 62693493 | 62962104 | | 0.04 | | SRGAP1,FLJ32549,LOC115749 | |
| 12q15 | 64277415 | 69714336 | | <0.001 | | LOC645253,HMGA2,LOC645270,MGC14817,TMBIM4,IRAK3,RBMS1P,LOC390340,HELB,GRIP1,LOC645305,GGTA1P,CAND1,LOC645328,DYRK2,LOC341333,IFNG,IL26,IL22,MDM1,LOC160410,LOC387867,RAP1B,LOC645422,LOC246723,NUP107,SLC35E3,**MDM2**,CPM,CPSF6,LYZ,YEATS4,FRS2,CCT2,LRRC10,VMD2L3,MGC13168,LOC645495,RAB3IP,LOC645507,C12orf28,CNOT2,KCNMB4,PTPRB,PTPRR | |
| 14q12 | 30069424 | 31579294 | | 0.007 | | SYF2P,KIAA1333,SCFD1,UBE2CP1,RPL12P5,RPL27P1,COCH,STRN3,AP4S1,HECTD1,NARSP,ATP5GP4,C14orf126,LOC644223,GPR33,NUBPL | |
| 14q12 | 31714140 | 31888792 | | 0.049 | | LOC644295,AKAP6 | |
| 14q12 | 32206647 | 32256360 | | 0.016 | | AKAP6 | |
| 14q13.3 | 32542174 | 40394768 | | <0.001 | | NPAS3,EGLN3,LOC644347,LOC644360,C14orf147,LOC644384,C14orf11,SNX6,RPL23AP9,CFL2,RPL12P6,BAZ1A,LOC390466,LOC338902,LOC441682,SRP54,C14orf24,C14orf10,KIAA0391,CDC10P,MRP63P8,DPRXP3,PSMA6,LOC122589,NFKBIA,DNAJC8P1,LOC122592,INSM2,GARNL1,NUTF2P2,BRMS1L,LOC644584,LOC644616,MBIP,STELLAR,TITF1,PHKBP2,NKX2-8,FLJ42220,LOC644719,PAX9,SLC25A21,MIPOL1,LOC644765,FOXA1,TTC6,SSTR1,CLEC14A,LOC390470,LOC283547,SEC23A,PPIAP4,SIP1,TRAPPC6B,PNN,YTHDF2P,MIA2,CTAGE5,COILP,FBXO33,LOC644898 | |
| 14q21.1 | 40893640 | 40894194 | | 0.05 | | NA | |
| 15q26.1 | 91980364 | 91980436 | | 0.005 | | NA | |
| 17p12 | 10832202 | 10836404 | | <0.001 | | NA | |
| 17q12 | 34950738 | 35394453 | | 0.027 | | NEUROD2,PPP1R1B,STARD3,TCAP,PNMT,PERLD1,**ERBB2**,C17orf37,GRB7,ZNFN1A3,ZPBP2,GSDML,ORMDL3,GSDM1,PSMD3 | |
| 19q13.11 | 34102452 | 35390533 | | <0.001 | | UQCRFS1,LOC284395,LOC342865,POP4,PLEKHF1,C19orf12,**CCNE1**,LOC126170,C19orf2,TAF2GL | |
| 19q13.11 | 35417874 | 35438593 | | 0.004 | | NA | |
| 19q13.11 | 35471315 | 35877699 | | 0.01 | | ZNF536 | |
| 22q11.21 | 19079060 | 19785571 | | 0.001 | | ZNF74,SCARF2,KLHL22,LOC643102,PCQAP,LOC645280,SLC9A3P2,LOC150207,LOC645289,POM121L4P,DKFZp434N035,LOC653603,PIK4CA,SERPIND1,SNAP29,CRKL,LOC400890,AIFL,LZTR1,THAP7,MGC16703,LOC645341,P2RXL1,SLC7A4,LOC653608,LOC400891,LOC645348 | |
| Xq27.3 | 142110655 | 143129472 | | 0.021 | | SPANX-N3,MYCL3,SLITRK4,SPANX-N2,UBE2NL | |
| Xq27.3 | 143170135 | 143223402 | | 0.038 | | RRM2P4 | |
| Xq27.3 | 143543451 | 154479679 | | <0.001 | | CYCSP44,SPANX-N1,LOC347422,LOC642408,SLITRK2,LOC653092,CXorf1,LOC653103,LOC642615,FMR1,FMR1NB,FTHL8,LOC642751,LOC642768,AFF2,LOC642804,IDS,LOC653157,IDSP1,LOC642875,CXorf40A,LOC642889,LOC653229,LW-1,FAM11A,LOC392556,LOC642915,MAGEA11,LOC642925,LOC642932,LOC653169,LOC653176,MAGEA9,MAGEA7,MAGEA8,LOC642980,CXorf40B,LOC643293,LOC643015,LOC389901,CXorf6,LOC653195,MTM1,MTMR1,LOC653204,CD99L2,LOC653286,HMGB3,LOC392557,GPR50,LOC286456,LOC203547,PASD1,PRRG3,FATE1,CNGA2,MAGEA4,GABRE,LOC401620,MAGEA5,MAGEA10,GABRA3,LOC402429,GABRQ,LOC643372,MAGEA6,CSAG3A,CSAG2,LOC653310,LOC653297,MAGEA12,CSAG1,MAGEA2,MAGEA2B,LOC643425,MAGEA3,psMAGEA,CETN2,NSDHL,LOC643469,ZNF185,PNMA5,PNMA3,PNMA6A,LOC653313,MAGEA1,LOC139046,ZNF275,LOC643656,LOC392559,LOC139735,TREX2,UIP1,LOC389904,BGN,ATP2B3,FAM58A,LOC340598,DUSP9,LOC347544,PNCK,SLC6A8,BCAP31,ABCD1,PLXNB3,STK23,IDH3G,SSR4,PDZK4,CYCSP45,L1CAM,LOC643736,AVPR2,ARHGAP4,ARD1A,RENBP,HCFC1,CXorf12,IRAK1,MECP2,OPN1LW,LOC653363,OPN1MW,LOC653367,LOC653368,CXorf2,TKTL1,FLNA,EMD,RPL10,RNU70,DNASE1L1,TAZ,ATP6AP1,GDI1,FAM50A,PLXNA3,DXS9879E,UBL4A,SLC10A3,FAM3A,G6PD,IKBKG,LOC643894,LOC653387,CTAG1A,CTAG1B,LOC340600,deltaNEMO,CTAG2,OR3B1P,GAB3,DKC1,MPP1,LOC158957,F8,H2AFB1,F8A1,LOC553820,FUNDC2,MTCP1,BRCC3,VBP1,RAB39B,CLIC2,PHF10P1,LOC553939,LOC401622,H2AFB2,F8A2,F8A3,H2AFB3,LOC401623,TMLHE | |
| ***Deletion*** | | | | | | | |
| 1p35.2 | 30408227 | | 30408889 | | 0.011 | | NA |
| 1q23.3 | 162386509 | | 162386527 | | <0.001 | | NA |
| 2p24.3 | 14657379 | | 14660619 | | <0.001 | | NA |
| 2q37.3 | 242692632 | | 242730382 | | <0.001 | | LOC653224,LOC653487 |
| 3p23 | 31478036 | | 31478423 | | <0.001 | | NA |
| 3p23 | 31478724 | | 31478724 | | 0.002 | | NA |
| 3p21.1 | 53003415 | | 53013826 | | <0.001 | | SFMBT1 |
| 4p16.1 | 9950895 | | 9952251 | | 0.009 | | NA |
| 4p13 | 42698103 | | 42747028 | | 0.035 | | LOC389207 |
| 4p13 | 42987706 | | 42988442 | | <0.001 | | NA |
| 5p15.33 | 3710491 | | 3710787 | | 0.012 | | NA |
| 6p22.1 | 29979615 | | 30006876 | | <0.001 | | 3.8-1.3,HCP5P6,HCG4P6,P5-05,HLA-K |
| 6q14.1 | 77497537 | | 77497656 | | <0.001 | | NA |
| 7q11.22 | 69782155 | | 69782593 | | <0.001 | | AUTS2 |
| 8p23.2 | 2587479 | | 4154189 | | 0.004 | | **CSMD1** |
| 8p23.2 | 5591708 | | 5591735 | | <0.001 | | NA |
| 8q24.21 | 128350890 | | 128351093 | | <0.001 | | NA |
| 9p23 | 9450595 | | 9479179 | | 0.049 | | NA |
| 9p22.2 | 17626427 | | 17626674 | | 0.016 | | SH3GL2 |
| 9p21.3 | 21252516 | | 21352014 | | 0.041 | | IFNAP22,IFNA5,KLHL9,IFNA6 |
| 9p21.3 | 21397375 | | 21806214 | | <0.001 | | IFNA8,LOC646581,IFNA1,IFNWP19,IFNE1,LOC402359,MTAP |
| 9p21.3 | 21845096 | | 21919666 | | <0.001 | | MTAP |
| 9p21.3 | 21934818 | | 21981752 | | 0.009 | | C9orf53,**CDKN2A** |
| 9p21.3 | 21999960 | | 22166961 | | <0.001 | | **CDKN2B** |
| 9p21.3 | 22194445 | | 22273153 | | <0.001 | | NA |
| 9p21.3 | 22375189 | | 22754293 | | 0.003 | | DMRTA1,FLJ35282,LOC646609 |
| 9p21.3 | 22791249 | | 22880200 | | <0.001 | | FLJ35282,LOC646611 |
| 9p21.3 | 22910664 | | 23021511 | | 0.011 | | NA |
| 9p21.3 | 23113107 | | 24607036 | | 0.041 | | LOC402360,ELAVL2,LOC646636 |
| 10q21.2 | 62228536 | | 62228666 | | <0.001 | | NA |
| 11p15.4 | 4232580 | | 4232709 | | <0.001 | | NA |
| 11p15.3 | 12293888 | | 12293899 | | <0.001 | | MICALCL |
| 12p13.2 | 10806814 | | 10806833 | | <0.001 | | LOC644286 |
| 12q21.31 | 82715458 | | 82715606 | | <0.001 | | NA |
| 13q12.12 | 23431606 | | 23432112 | | <0.001 | | NA |
| 13q33.1 | 102320968 | | 102321068 | | <0.001 | | **ERCC5** |
| 15q11.1 | 19840684 | | 19841990 | | <0.001 | | NA |
| 16q23.1 | 77434802 | | 77434919 | | <0.001 | | WWOX |
| 17p12 | 10832202 | | 10836404 | | <0.001 | | NA |
| 17q22 | 51518046 | | 51520028 | | 0.004 | | NA |
| 17q25.1 | 74757811 | | 74757985 | | <0.001 | | LOC146713 |
| 18q11.2 | 24449765 | | 24450128 | | <0.001 | | NA |
| 18q21.31 | 55293097 | | 55293760 | | 0.017 | | **CCBE1** |
| 18q22.1 | 62060664 | | 62060963 | | <0.001 | | NA |
| 18q22.1 | 64902105 | | 64903434 | | 0.005 | | NA |
| 19q13.33 | 48395295 | | 48422999 | | 0.01 | | PSG11,PSG4,CEACAMP10 |
| 19q13.42 | 53881227 | | 53888534 | | <0.001 | | NA |
| 20q13.31 | 55040991 | | 55041191 | | 0.005 | | NA |
| 21q22.2 | 41332614 | | 41332735 | | <0.001 | | NA |

**Supplementary Table 7.** Details about the implicated SCAs and full list of genes covered by these SCAs, derived from individual chromosome analysis of the lung adenocarcinoma data set. (Start: Start point of SCAs. End: End point of SCAs.) (Based on hg18 human genome assembly)

| **Cyto-**  **band** | **Region** | | **P-value** | **Genes covered by the SCA regions** |
| --- | --- | --- | --- | --- |
|  | **Start** | **End** |  |  |
| ***Amplification*** | | | | |
| 1p31.1 | 77219051 | 77219148 | <0.001 | ST6GALNAC5 |
| 1q21.1 | 143482819 | 153696619 | <0.001 | PDE4DIP,SEC22L1,NUDT4P1,NOTCH2NL,LOC644474,HFE2,TXNIP,POLR3GL,ANKRD34,LIX1L,RBM8A,GNRHR2,PEX11B,ITGA10,ANKRD35,PIAS3,NUDT17,POLR3C,ZNF364,CD160,PDZK1,LOC653519,LOC653397,LOC644796,NBPF11,LOC644034,LOC653401,LOC644821,NBPF12,LOC644833,LOC644060,LOC441904,LOC644861,PRKAB2,PDIA3P,FMO5,LOC644131,CHD1L,OR13Z1P,OR13Z2P,BCL9,LOC644880,ACP6,GJA5,LOC391092,GJA8,GPR89,LOC644930,NBPF9,LOC644262,LOC653438,LOC644963,FLJ39739,LOC645026,FAM91A3P,LOC645052,PPIAL4,NBPF14,LOC653564,LOC388687,NBPF10,LOC642516,LOC645116,NBPF15,LOC388686,LOC645126,LOC644291,LOC645142,LOC645146,LOC645154,LOC645159,DRD5P2,LOC645166,LOC388689,LOC401967,LOC645184,RNU1P9,LOC645239,LOC388692,LOC645262,LOC644410,LOC653593,LOC440686,LOC653594,LOC653598,LOC440685,FAM91A2,LOC653496,LOC644634,LOC199882,FCGR1A,HIST2H2BF,LOC653604,HIST2H3A,HIST2H2AA,HIST2H2BC,LOC653610,LOC653611,HIST2H4,HIST2H2BE,HIST2H2AC,HIST2H2AB,BOLA1,SV2A,SF3B4,MTMR11,ZA20D1,LOC441907,VPS45A,PLEKHO1,ANP32E,CA14,APH1A,C1orf54,C1orf51,MRPS21,PRPF3,KIAA0460,TARSL1,ECM1,ADAMTSL4,C1orf138,MCL1,ENSA,GOLPH3L,HORMAD1,CTSS,CTSK,**ARNT**,CYCSP51,SETDB1,LASS2,ANXA9,FAM63A,PRUNE,BNIPL,C1orf56,CDC42SE1,MLLT11,MGC29891,SEMA6C,TNFAIP8L2,LYSMD1,SCNM1,TMOD4,VPS72,PIP5K1A,PSMD4,ZNF687,PIK4CB,RFX5,SELENBP1,PSMB4,POGZ,CGN,TUFT1,SNX27,TNRC4,FLJ36032,LOC645469,LOC645473,MRPL9,OAZ3,TDRKH,LRRN6D,RORC,THEM5,LOC645499,THEM4,LOC391099,S100A10,LOC441908,S100A11,LOC645527,LOC645539,THHL1,THH,HDHD1CP,RPTN,HRNR,FLG,FLG2,CRNN,LCE5A,C1orf42,LCE3E,LCE3D,LCE3C,LCE3B,LCE3A,LCE2D,LCE2C,LCE2B,LCE2A,LCE4A,C1orf45,LCE1F,LCE1E,LCE1D,LCE1C,LCE1B,LCE1A,SMCP,IVL,SPRR4,SPRR1A,SPRR3,LOC645810,LOC645813,SPRR1B,SPRR2D,SPRR2A,SPRR2B,SPRR2E,SPRR2F,SPRR2C,SPRR2G,LELP1,LOC391102,LOR,PGLYRP3,PGLYRP4,S100A9,S100A12,LOC645900,S100A8,LOC645908,S100A7L1,LOC645922,S100A7,S100A6,S100A5,S100A4,S100A3,S100A2,S100A16,S100A14,S100A13,S100A1,C1orf77,SNAPAP,ILF2,NPR1,C1orf60,SLC27A3,LOC343052,GATAD2B,LOC645965,KIAA0476,LOC645977,CRTC2,SLC39A1,CREB3L4,JTB,RAB13,RPS27,NUP210L,TPM3,C1orf189,C1orf43,UBAP2L,HAX1,LOC653690,AQP10,ATP8B2,MRPS33P1,IL6R,SHE,TDRD10,UBE2Q1,CHRNB2,ADAR,KCNN3,PMVK,PBXIP1,PYGO2,SHC1,CKS1B,FLAD1,LENEP,ZBTB7B,DCST2,DCST1,ADAM15,EFNA4,EFNA3,EFNA1,RAG1AP1,DPM3,KRTCAP2,TRIM46,MUC1,THBS3,MTX1,GBAP,MTX1P,GBA,C1orf2,SCAMP3,CLK2,HCN3,PKLR,FDPS,C1orf104,RUSC1,ASH1L,LOC645682 |
| 1q23.1 | 156873345 | 157646599 | 0.019 | SPTA1,OR6K1P,OR6K2,OR6K3,OR6K4P,OR6K5P,OR6K6,OR6N1,OR6N2,OR2AQ1P,OR10AA1P,MNDA,LOC646377,PYHIN1,LOC646381,IFI16,AIM2,IGSF4B,FY,LOC127150,OR10J2P,FCER1A,OR10J3,OR10J7P,OR10J8P,OR10J9P |
| 1q23.1 | 158365802 | 159117169 | 0.004 | ATP1A2,ATP1A4,CASQ1,PEA15,WDR42A,LOC653750,LOC388707,PEX19,COPA,LOC474338,NCSTN,NHLH1,VANGL2,SLAMF6,CD84,SLAMF1,CD48,SLAMF7,LY9,CD244,ITLN1 |
| 1q23.3 | 160959095 | 162648690 | 0.002 | DDR2,HSD17B7,C1orf110,RGS4,LOC646617,RGS5,CDCA1,LOC646634 |
| 1q23.3 | 162691625 | 174470888 | <0.001 | PBX1,LMX1A,RXRG,LRRC52,LOC401974,MGST3,ALDH9A1,LOC440700,TMCO1,LOC391126,UCK2,LOC401975,LOC646676,FAM78B,LOC284685,LOC116123,LOC646688,LOC646690,LOC646693,POGK,TADA1L,C1orf32,MAEL,GPA33,DUSP27,LOC391130,POU2F1,CD3Z,SRD5BP1,CREG1,RCSD1,MPZL1,SAC,BRP44,IQWD1,LOC653763,GPR161,TIPRL,RPL34P1,SFT2D2,MGC12538,TBX19,LOC441914,XCL2,XCL1,DPT,bA375F2.1,LOC646757,LOC391132,ATP1B1,NME7,BLZF1,C1orf114,SLC19A2,LOC646771,F5,SELP,SELL,SELE,LOC646776,C1orf156,C1orf112,SCYL3,KIFAP3,MRPS10P1,SIGLECP14,C1orf184,LOC646796,SCYL1BP1,PRRX1,LOC646669,C1orf129,LOC646804,FMO3,FMO6,FMO2,FMO1,FMO4,TOP1P1,CYCSP53,BAT2D1,MYOC,LOC391135,VAMP4,LOC391136,KIAA0859,LOC391137,LOC127099,DNM3,C1orf105,PIGC,C1orf9,FASLG,LOC441915,TNFSF18,LOC391139,LOC646861,TNFSF4,LOC646870,PRDX6,SLC9A11,ANKRD45,KLHL20,C1orf155,DARS2,GAS5,ZBTB37,SERPINC1,RC3H1,KIAA0492,RABGAP1L,GPR52,CACYBP,MRPS14,LOC646891,TNN,KIAA0040,LOC646905,TNR,RFWD2,LOC391140 |
| 1q25.2 | 179012256 | 179015228 | 0.046 | XPR1 |
| 1q32.3 | 212361064 | 212361947 | <0.001 | NA |
| 2p25.1 | 10320573 | 10320747 | 0.007 | NA |
| 2p15 | 61881898 | 62984845 | 0.006 | FLJ13305,LOC647082,CCT4,COMMD1,B3GNT1,LOC647093,TMEM17,EHBP1 |
| 3q26.31 | 171616341 | 172210360 | 0.006 | CLDN11,FLJ37228,SLC7A14,LOC402149,LOC440990,EIF5A2,LOC442096,SLC2A2 |
| 4q12 | 57179959 | 60216021 | <0.001 | HOP,LOC391655,LOC285453,SPINK2,REST,C4orf14,POLR2B,IGFBP7,LOC255130,SRIL |
| 5p15.33 | 208367 | 12445539 | <0.001 | KIAA1909,LOC389257,LOC133957,SDHA,PDCD6,AHRR,LOC116349,EXOC3,SLC9A3,CEP72,TPPP,LOC653082,LOC643702,LOC643740,ZDHHC11,LOC653350,BRD9,TRIP13,NKD2,SLC12A7,SLC6A19,SLC6A18,**TERT**,CRR9,SLC6A3,AYTL2,LOC653102,LOC642535,LOC653378,LOC442128,MRPL36,NDUFS6,LOC389267,LOC644065,IRX4,IRX2,CEI,LOC285577,IRX1,LOC340094,ADAMTS16,LOC442131,KIAA0947,LOC645267,FLJ33360,TRG20,FLJ25076,NSUN2,SRD5A1,POLS,LOC645463,LOC645451,LOC442132,ADCY2,LOC134121,MGC5297,MTRR,LOC645502,LOC645583,LOC645607,SEMA5A,TAS2R1,LOC134145,CCT5,LOC134147,MARCH6,ROPN1L,LOC645735,LOC345711,LOC645763,DAP,**CTNND2** |
| 5p15.2 | 12517763 | 15455950 | 0.001 | LOC645817,LOC391738,DNAH5,TRIO,FAM105A,LOC645894,LOC391739,EEF1AL11,FAM105B,ANKH,LOC642954 |
| 5p15.1 | 16112968 | 25426220 | 0.049 | LOC441061,ZNF622,FLJ20152,MYO10,LOC643003,FLJ34047,BASP1,LOC646012,FTHL10,LOC285697,LOC340096,LOC646032,LOC391742,LOC391745,LOC391746,LOC646066,LOC391747,LOC402199,LOC391749,LOC285563,LOC402200,LOC402201,LOC646103,LOC402203,LOC202201,LOC402205,LOC646126,LOC402207,LOC402208,LOC646152,LOC646165,LOC391761,LOC646188,LOC391763,LOC391764,LOC391765,LOC391766,LOC391767,LOC391768,LOC391769,LOC391770,LOC646241,LOC646273,CDH18,LOC646280,LOC266786,LOC646296,LOC646326,LOC646335,LOC646351,CDH12,LOC643288,LOC643300,PMCHL1,LOC391771,LOC646393,LOC646398,PRDM9,LOC439936,LOC503540,CDH10,LOC646435,LOC643379 |
| 5p13.2 | 34629580 | 34640393 | 0.001 | NA |
| 5p13.2 | 37258798 | 37616194 | 0.01 | LOC441068,NUP155,WDR70 |
| 6p22.1 | 30260012 | 30911233 | 0.027 | TRIM26,HLA-L,FLJ45422,LOC646491,HCG18,TRIM39,RPP21,MICC,LOC646520,RANP1,HLA-E,GNL1,PRR3,LOC646536,ABCF1,PPP1R10,MRPS18B,C6orf134,PTMAP1,C6orf136,DHX16,KIAA1949,NRM,LOC646546,MDC1,TUBB,FLOT1,IER3,LOC646553 |
| 6p21.1 | 43788317 | 44110351 | 0.004 | VEGF,MGC45491 |
| 7p21.2 | 14380867 | 19017513 | <0.001 | DGKB,FLJ16237,MEOX2,LOC442511,SOSTDC1,LOC442512,LOC317727,ANKMY2,BZW2,TSPAN13,AGR2,LOC646252,BCMP11,RAD17P1,LOC646265,LOC653724,AHR,SNX13,LOC442285,LOC442658,PRPS1L1,LOC646302,HDAC9,NPM1P13 |
| 7p21.1 | 19161066 | 19980905 | 0.001 | TWISTNB,MGC42090 |
| 7p21.1 | 20370169 | 21247850 | 0.001 | ITGB8,LOC646385,ABCB5,SP8,LOC222901,LOC340274 |
| 7p15.3 | 22314303 | 32358683 | <0.001 | LOC646468,LOC221838,IL6,RPS26e,TOMM7,LOC392872,DRCTNNB1A,KLHL7,NUPL2,GPNMB,C7orf30,IMP-3,MGC27348,TRA2A,CLK2P,LOC646524,LOC90693,MGC72075,STK31,NPY,MPP6,DFNA5,OSBPL3,CYCS,C7orf31,LOC392005,C7orf9,LOC646588,UBA52P1,NFE2L3,HNRPA2B1,CBX3,LOC442659,SNX10,LOC441204,LOC441205,LOC285941,SCAP2,LOC442660,LOC442661,LOC402643,LOC442290,HOXA1,HOXA2,HOXA3,LOC643903,HOXA4,HOXA5,HOXA6,HOXA7,HOXA9,HOXA10,HOXA11,LOC646692,HOXA13,EVX1,LOC392008,LOC401316,LOC442663,LOC442664,HIBADH,LOC646726,TAX1BP1,JAZF1,LOC402644,CREB5,LOC401317,KIAA0644,CPVL,LOC644086,CHN2,NANOGP4,LOC646745,LOC222171,LOC646762,LOC646767,DPY19L2P3,LOC644150,SCRN1,FKBP14,PLEKHA8,Ells1,ZNRF2,DKFZp586I1420,CARD4,C7orf24,LOC401320,GARS,CRHR2,INMT,FLJ22374,AQP1,GHRHR,ADCYAP1R1,NEUROD6,LOC223075,C7orf16,PDE1C |
| 7p12.1 | 50629718 | 56118914 | <0.001 | GRB10,COBL,LOC642663,LOC642878,LOC392027,DKFZp564N2472,LOC653175,FLJ45974,LOC222005,MGC33530,LOC392030,SEC61G,LOC643168,EGFR,CALM1P2,LANCL2,ECOP,LOC643336,LOC442308,LOC643348,FKBP9L,LOC653281,LOC643357,LOC643362,PSPHL,FLJ44060,LOC643399,FLJ39963,MRPS17,GBAS,PSPH,CCT6A,SUMF2,PHKG1 |
| 7p11.2 | 56325172 | 57336046 | <0.001 | LOC441228,LOC154937,LOC643497,LOC643504,LOC254027,LOC346296,LOC643546,LOC401357,LOC643574,LOC643595,LOC402508,LOC340221,LOC402509,LOC643620,ZNF479,LOC643629,LOC643632 |
| 8q21.13 | 80482123 | 82281412 | 0.005 | STMN2,HEY1,MRPS28,TPD52,LOC389671,LOC402342,LOC340443,ZBTB10,LOC389672,LOC646437,ZNF704,CKS1A,PAG1,LOC653745,LOC653746 |
| 8q24.13 | 124498576 | 131910999 | <0.001 | C8orf32,FBXO32,C8ORFK36,ANXA13,FAM91A1,C8orf54,FLJ32770,LOC392268,LOC442396,LOC645063,LOC392269,TMEM65,TRMT12,RNF139,TATDN1,NDUFB9,MTSS1,ZNF572,SQLE,KIAA0196,C8orf36,TRIB1,LOC645274,FAM84B,LOC645290,SRRM1L,POU5F1P1,MYC,PVT1,LOC441378,CCDC26,MLZE,FAM49B,DDEF1,ADCY8 |
| 10p14 | 9835332 | 9836744 | 0.015 | LOC644495 |
| 11q13.3 | 68177770 | 70461200 | 0.003 | GAL,MTL5,CPT1A,MRPL21,IGHMBP2,MRGPRD,MRGPRF,TPCN2,MYEOV,LOC390218,CCND1,FLJ42258,ORAOV1,FGF19,FGF4,FGF3,TMEM16A,FADD,PPFIA1,CTTN,SHANK2,LOC653621,LOC220070,LOC645416 |
| 12p12.1 | 24055630 | 26685022 | 0.002 | SOX5,FLJ32894,BCAT1,LOC441630,LOC645167,LOC645177,LOC645186,LRMP,CASC1,LOC144363,KRAS,FLJ36004,LOC645233,RASSF8,BHLHB3,SSPN,ITPR2 |
| 12q13.2 | 55942972 | 57235376 | <0.001 | KIAA1002,INHBC,INHBE,GLI1,ARHGAP9,MARS,DDIT3,MBD6,DCTN2,KIF5A,PIP5K2C,DTX3,GEFT,SLC26A10,B4GALNT1,LOC441641,OS9,CENTG1,TSPAN31,CDK4,MARCH9,CYP27B1,METTL1,DKFZP586D0919,TSFM,AVIL,CTDSP2,XRCC6BP1,LOC338805 |
| 12q14.1 | 61896783 | 70622255 | 0.001 | DPY19L2,LOC390338,TMEM5,LOC341315,SRGAP1,FLJ32549,LOC115749,XPOT,LOC653581,TBK1,RASSF3,GNS,KIAA0984,WIF1,LEMD3,MSRB3,LOC645253,HMGA2,LOC645270,MGC14817,TMBIM4,IRAK3,RBMS1P,LOC390340,HELB,GRIP1,LOC645305,GGTA1P,CAND1,LOC645328,DYRK2,LOC341333,IFNG,IL26,IL22,MDM1,LOC160410,LOC387867,RAP1B,LOC645422,LOC246723,NUP107,SLC35E3,**MDM2**,CPM,CPSF6,LYZ,YEATS4,FRS2,CCT2,LRRC10,VMD2L3,MGC13168,LOC645495,RAB3IP,LOC645507,C12orf28,CNOT2,KCNMB4,PTPRB,PTPRR,TSPAN8,LGR5,PSRC2,THAP2,TMEM19,RAB21,TBC1D15,TPH2 |
| 13q33.3 | 108850730 | 111141242 | 0.049 | IRS2,COL4A1,COL4A2,RAB20,LOC644583,FLJ10769,FLJ12118,ING1,LOC644627,LOC283487,ANKRD10,PARP1P1,LOC644725,ARHGEF7,LOC121792,MGC35169 |
| 14q11.2 | 19487234 | 23376204 | <0.001 | OR4K16P,OR4K15,OR4Q2P,OR4K14,OR4K13,OR4U1P,OR4L1,OR4T1P,OR4K17,OR4N5,OR11G1P,OR11P1P,OR11G2,OR11H5P,OR11H6,OR11H7P,OR11H4,TTC5,CCNB1IP1,RPPH1,PARP2,TEP1,LOC123103,LOC441672,OSGEP,APEX1,TMEM55B,NP,RNASE10,RNASE9,RNASE11,RNASE12,LOC643145,LOC254028,OR6S1,RNASE4,ANG,RANBP20P,FAM12A,FAM12B,RNASE6,RNASE1,RNASE3,LOC643332,RNASE2,FLJ20859,SLC39A2,NDRG2,C14orf8,RNASE13,RNASE7,RNASE8,FLJ10357,ZNF219,LOC643382,LOC554207,OR5AU1,LOC401744,HNRPC,RPGRIP1,MRPS17P6,SUPT16H,CHD8,UBA52P2,EIF4EBP1P,RAB2B,C14orf92,TRA@,TRAV1-1,TRAV1-2,TRAV2,TRAV3,TRAV4,TRAV5,TRAV6,TRAV7,TRAV8-1,TRAV9-1,TRAV10,TRAV11,TRAV12-1,TRAV8-2,TRAV8-3,TRAV13-1,TRAV12-2,TRAV8-4,TRAV8-5,TRAV13-2,TRAV14DV4,TRAV9-2,TRAV15,TRAV12-3,TRAV8-6,TRAV16,TRAV17,TRAV18,TRAV19,TRAV20,TRAV21,TRAV22,TRAV23DV6,TRDV1,TRAV24,TRAV25,TRAV26-1,TRAV8-7,TRAV27,TRAV28,TRAV29DV5,TRAV30,TRAV31,TRAV32,TRAV33,TRAV26-2,TRAV34,TRAV35,TRAV36DV7,TRAV37,TRAV38-1,TRAV38-2DV8,TRAV39,TRAV40,TRAV41,TRD@,TRDV2,TRDD1,TRDD2,TRDD3,TRDJ1,TRDJ4,TRDJ2,TRDJ3,TRDC,TRDV3,TRAJ61,TRAJ60,TRAJ59,TRAJ58,TRAJ57,TRAJ56,TRAJ55,TRAJ54,TRAJ53,TRAJ52,TRAJ51,TRAJ50,TRAJ49,TRAJ48,TRAJ47,TRAJ46,TRAJ45,TRAJ44,TRAJ43,TRAJ42,TRAJ41,TRAJ40,TRAJ39,TRAJ38,TRAJ37,TRAJ36,TRAJ35,TRAJ34,TRAJ33,TRAJ32,TRAJ31,TRAJ30,TRAJ29,TRAJ28,TRAJ27,TRAJ26,TRAJ25,TRAJ24,TRAJ23,TRAJ22,TRAJ21,TRAJ20,TRAJ19,TRAJ18,TRAJ17,TRAJ16,TRAJ15,TRAJ14,TRAJ13,TRAJ12,TRAJ11,TRAJ10,TRAJ9,TRAJ8,TRAJ7,TRAJ6,TRAJ5,TRAJ4,TRAJ3,TRAJ2,TRAJ1,TRAC,DAD1,ABHD4,OR6J1,RPL26P2,OR6E1P,LOC643639,OXA1L,SLC7A7,MRPL52,MMP14,LRP10,FLJ38964,RBM23,SKB1,TRR,C14orf94,JUB,C14orf93,PSMB5,FLJ16369,CDH24,ACIN1,C14orf119,LOC643712,CEBPE,SLC7A8,RPL39P2,HMGN2P,KIAA1443,PPP1R3E,BCL2L2,PABPN1,SLC22A17,EFS,LOC643759,IL17E,CMTM5,MYH6,MYH7,C14orf120,ZFHX2,ZNF409,THTPA,AP1G2,JPH4,DHRS2,BRD7P,LOC643819 |
| 14q12 | 28133356 | 29296120 | <0.001 | BTF3P2,FOXG1B,LOC387978,LOC644095,PRKD1 |
| 14q12 | 30069424 | 40894194 | <0.001 | SYF2P,KIAA1333,SCFD1,UBE2CP1,RPL12P5,RPL27P1,COCH,STRN3,AP4S1,HECTD1,NARSP,ATP5GP4,C14orf126,LOC644223,GPR33,NUBPL,C14orf128,ARHGAP5,LOC644295,AKAP6,MTCO1P2,NPAS3,EGLN3,LOC644347,LOC644360,C14orf147,LOC644384,C14orf11,SNX6,RPL23AP9,CFL2,RPL12P6,BAZ1A,LOC390466,LOC338902,LOC441682,SRP54,C14orf24,C14orf10,KIAA0391,CDC10P,MRP63P8,DPRXP3,PSMA6,LOC122589,NFKBIA,DNAJC8P1,LOC122592,INSM2,GARNL1,NUTF2P2,BRMS1L,LOC644584,LOC644616,MBIP,STELLAR,TITF1,PHKBP2,NKX2-8,FLJ42220,LOC644719,PAX9,SLC25A21,MIPOL1,LOC644765,FOXA1,TTC6,SSTR1,CLEC14A,LOC390470,LOC283547,SEC23A,PPIAP4,SIP1,TRAPPC6B,PNN,YTHDF2P,MIA2,CTAGE5,COILP,FBXO33,LOC644898,LOC644919 |
| 14q21.1 | 41384303 | 41400655 | 0.002 | LRFN5 |
| 14q22.1 | 52946657 | 53701181 | 0.018 | BMP4,ATP5C2,LOC645560 |
| 15q22.2 | 59690766 | 59690895 | 0.017 | NA |
| 15q26.1 | 91980364 | 91980436 | <0.001 | NA |
| 17p12 | 10832202 | 10836404 | <0.001 | NA |
| 17q11.2 | 31471515 | 31496575 | 0.007 | NA |
| 17q12 | 33754231 | 35394453 | <0.001 | SOCS7,ARHGAP23,SNIP,LOC642705,MLLT6,PCGF2,PSMB3,PIP5K2B,CCDC49,LOC388381,RPL23,LASP1,LOC642453,FBXO47,LOC642785,FLJ43826,LOC642808,PLXDC1,ARL5C,CACNB1,RPL19,STAC2,LOC642851,FBXL20,PPARBP,CRKRS,NEUROD2,PPP1R1B,STARD3,TCAP,PNMT,PERLD1,**ERBB2**,C17orf37,GRB7,ZNFN1A3,ZPBP2,GSDML,ORMDL3,GSDM1,PSMD3 |
| 17q21.31 | 41564110 | 41708649 | 0.004 | KIAA1267,LOC644246,LOC644253,LOC644256,LOC644264 |
| 17q22 | 51766507 | 51767107 | 0.049 | ANKFN1 |
| 18q11.2 | 21613135 | 21881136 | 0.036 | **SS18** |
| 18q11.2 | 22222293 | 22238939 | 0.027 | TAF4B |
| 19q13.11 | 34102452 | 36082303 | 0.006 | UQCRFS1,LOC284395,LOC342865,POP4,PLEKHF1,C19orf12,CCNE1,LOC126170,C19orf2,TAF2GL,LOC642948,ZNF536 |
| 20q11.21 | 29419108 | 29789374 | 0.01 | DEFB118,DEFB119,DEFB121,DEFB123,REM1,HM13,ID1,COX4I2,BCL2L1 |
| 20q11.22 | 32306194 | 37480974 | <0.001 | ASIP,AHCY,ITCH,CDC42P1,FDXP1,FLJ38773,DYNLRB1,MAP1LC3A,CDC91L1,LOC653678,TP53INP2,NCOA6,HMG4L,GGTL3,ACSS2,GSS,MYH7B,TRPC4AP,C20orf31,PROCR,C20orf127,MMP24,ITGB4BP,C20orf128,C20orf44,GDF5,CEP250,LOC343705,C20orf173,C20orf47,FER1L4,RPL37P1,SPAG4,CPNE1,RBM12,NFS1,C20orf52,RNPC2,PHF20,SCAND1,C20orf152,LOC644799,HMG4L2,EPB41L1,LOC646017,C20orf4,DLGAP4,MYL9,TGIF2,C20orf24,SLA2,NDRG3,C20orf172,C20orf117,KIAA0889,C20orf118,SAMHD1,RBL1,RPS3AP3,C20orf132,RPN2,GHRH,MANBAL,SRC,RPL7AL4,BLCAP,NNAT,PPIAP3,GLRXP,FLJ42133,CTNNBL1,C20orf102,KIAA0406,C20orf77,TGM2,KIAA1755,BPI,LBP,LOC388796,LOC128439,KIAA1219,RPS3P2,SMAF1,LOC391247,C20orf95,SLC32A1,ACTR5,PPP1R16B,C20orf129,DHX35,NPM1P19,LOC642900 |
| 20q13.13 | 48523002 | 48552963 | 0.015 | COX6CP2 |
| 20q13.2 | 51553048 | 51925779 | 0.035 | ZNF217,LOC391257 |
| 20q13.31 | 55553075 | 56208537 | 0.001 | PCK1,ZBP1,TMEPAI,LOC645322,C20orf85,LOC645365 |
| 20q13.33 | 59344243 | 62374173 | 0.001 | CDH4,TAF4,FAM61B,PSMA7,SS18L1,GTPBP5,HRH3,FLJ44790,OSBPL2,ADRM1,LAMA5,LOC645951,RPS21,CABLES2,C20orf151,GATA5,FLJ30313,C20orf166,RPL7P3,FLJ32154,SLCO4A1,NTSR1,C20orf20,OGFR,COL9A3,TCFL5,ARF4P2,DIDO1,C20orf11,C20orf59,BHLHB4,LOC646031,C20orf51,YTHDF1,BIRC7,C20orf58,ARFGAP1,COL20A1,CHRNA4,KCNQ2,EEF1A2,C20orf149,PTK6,SRMS,C20orf195,PRIC285,GMEB2,STMN3,RTEL1,TNFRSF6B,ARFRP1,ZGPAT,LIME1,SLC2A4RG,BTBD4,C20orf135,TPD52L2,DNAJC5,UCKL1,GM632,SAMD10,C20orf14,LOC284739,SOX18,TCEA2,RGS19,OPRL1,LOC198437,LOC646132,GPR8,MYT1,PCMTD2 |
| 22q11.1 | 16596539 | 16871076 | 0.003 | BID,MICAL3,LOC642566 |
| 22q11.1 | 17710168 | 17959242 | 0.02 | HIRA,MRPL40,LOC128977,UFD1L,CDC45L,CLDN5 |
| 22q11.21 | 18584041 | 20363012 | 0.011 | RTN4R,LOC440792,DGCR6L,LOC653184,LOC643002,LOC643009,LOC645205,LOC643024,KIAA1666,LOC645223,LOC440795,LOC653590,FLJ26056,LOC643069,LOC653203,USP41,ZNF74,SCARF2,KLHL22,LOC643102,PCQAP,LOC645280,SLC9A3P2,LOC150207,LOC645289,POM121L4P,DKFZp434N035,LOC653603,PIK4CA,SERPIND1,SNAP29,CRKL,LOC400890,AIFL,LZTR1,THAP7,MGC16703,LOC645341,P2RXL1,SLC7A4,LOC653608,LOC400891,LOC645348,FLJ42953,LOC376818,LOC284861,LOC653257,LOC645367,LOC645376,LOC643318,LOC653617,LOC653264,LOC653270,LOC645390,LOC388853,LOC440804,HIC2,LOC645426,LOC220686,LOC375133,LOC150221,UBE2L3,LOC150223,FLJ36046,SDF2L1,PPIL2 |
| ***Deletion*** | | | | |
| 1p35.2 | 30408227 | 30408889 | 0.001 | NA |
| 1q23.3 | 162262997 | 162386527 | <0.001 | NA |
| 1q42.2 | 233611648 | 233611670 | 0.027 | TBCE |
| 2p24.3 | 14657379 | 14660619 | <0.001 | NA |
| 2q36.1 | 223756194 | 223804101 | <0.001 | NA |
| 2q37.3 | 242634423 | 242730382 | <0.001 | LOC653224,LOC653487 |
| 3p23 | 31478036 | 31478724 | <0.001 | NA |
| 3p21.1 | 53003415 | 53013826 | <0.001 | SFMBT1 |
| 3q24 | 147793976 | 147804569 | 0.02 | PLSCR5 |
| 4p16.1 | 9950895 | 9952251 | <0.001 | NA |
| 4p13 | 42698103 | 42988442 | <0.001 | LOC389207 |
| 4q28.2 | 129348047 | 129348079 | <0.001 | LARP2 |
| 5p15.33 | 3710491 | 3710787 | <0.001 | NA |
| 5p15.1 | 15772222 | 15773478 | <0.001 | FBXL7 |
| 5q11.2 | 58658855 | 58764672 | 0.003 | PDE4D |
| 5q23.1 | 119043649 | 119043712 | 0.012 | NA |
| 6p22.1 | 29979615 | 30006876 | <0.001 | 3.8-1.3,HCP5P6,HCG4P6,P5-05,HLA-K |
| 6p21.33 | 31447975 | 31448054 | 0.002 | LOC442200 |
| 6p21.31 | 34822187 | 34822300 | 0.031 | NA |
| 6q14.1 | 77497537 | 77497656 | <0.001 | NA |
| 6q24.2 | 143902033 | 143902292 | 0.004 | NA |
| 6q26 | 162388858 | 162580130 | 0.019 | PARK2 |
| 7q11.22 | 69532764 | 69615810 | <0.001 | AUTS2 |
| 7q11.22 | 69782155 | 69782593 | <0.001 | AUTS2 |
| 8p23.2 | 2587479 | 4158386 | 0.032 | CSMD1 |
| 8p23.2 | 5591708 | 5591735 | <0.001 | NA |
| 8q24.21 | 128350890 | 128351093 | <0.001 | NA |
| 9p23 | 9450595 | 9479179 | 0.01 | NA |
| 9p22.2 | 17626427 | 17626674 | 0.002 | SH3GL2 |
| 9p21.3 | 21252516 | 24607036 | <0.001 | IFNAP22,IFNA5,KLHL9,IFNA6,IFNA13,IFNA2,IFNWP12,IFNA8,LOC646581,IFNA1,IFNWP19,IFNE1,LOC402359,MTAP,C9orf53,CDKN2A,CDKN2B,LOC646605,DMRTA1,FLJ35282,LOC646609,LOC646611,LOC402360,ELAVL2,LOC646636 |
| 10q11.23 | 52764624 | 52765125 | 0.028 | PRKG1 |
| 10q21.2 | 62228536 | 62228666 | <0.001 | NA |
| 11p15.4 | 4232580 | 4232709 | <0.001 | NA |
| 11p15.3 | 12293888 | 12293899 | <0.001 | MICALCL |
| 11p15.1 | 18905796 | 18913773 | 0.017 | MRGPRX1 |
| 11q22.1 | 101173276 | 101173540 | 0.006 | NA |
| 11q23.1 | 112314862 | 112348635 | <0.001 | NCAM1 |
| 11q23.3 | 119744397 | 119779421 | <0.001 | ARHGEF12 |
| 12p13.2 | 10806814 | 10806833 | <0.001 | LOC644286 |
| 12q21.31 | 82715458 | 82715606 | <0.001 | NA |
| 13q12.12 | 23431606 | 23432112 | <0.001 | NA |
| 13q14.2 | 47917390 | 47933694 | 0.041 | RB1 |
| 13q33.1 | 102320968 | 102321068 | <0.001 | ERCC5 |
| 14q21.1 | 38271727 | 38272441 | <0.001 | NA |
| 14q32.12 | 91925273 | 91938114 | 0.005 | SLC24A4 |
| 15q11.1 | 19827281 | 20059872 | <0.001 | OR4Q1P,OR4H6P,OR4M2,OR4N4,OR4N3P,LOC388076,IGHV1OR15-1,VSIG6,LOC646370,LOC646372,LOC646379,LOC646396 |
| 15q13.1 | 30297184 | 30848487 | 0.011 | LOC644110,LOC644153,LOC653125,FAM7A1,LOC440266,LOC644208,LOC400352,LOC644231,LOC642711,LOC440267,ARHGAP11A,SGNE1,LOC642756,GREM1,FMN1 |
| 15q26.1 | 90532323 | 90532460 | <0.001 | NA |
| 16q21 | 64436543 | 64436948 | 0.005 | NA |
| 16q22.2 | 72306271 | 72307496 | 0.021 | NA |
| 16q23.1 | 77434802 | 77434919 | <0.001 | WWOX |
| 17p12 | 10832202 | 10836404 | <0.001 | NA |
| 17p11.2 | 19458875 | 19478062 | 0.014 | LOC645221 |
| 17q22 | 51518046 | 51520028 | <0.001 | NA |
| 17q22 | 52133816 | 52134480 | 0.019 | NA |
| 17q22 | 53179948 | 53180469 | 0.036 | NA |
| 17q25.1 | 74757811 | 74757985 | <0.001 | LOC146713 |
| 18q11.2 | 24449765 | 24450128 | <0.001 | NA |
| 18q21.31 | 55293097 | 55293760 | <0.001 | CCBE1 |
| 18q22.1 | 62060664 | 62060963 | <0.001 | NA |
| 18q22.1 | 64902105 | 64903434 | <0.001 | NA |
| 19q13.33 | 48395295 | 48422999 | <0.001 | PSG11,PSG4,CEACAMP10 |
| 19q13.42 | 53881227 | 53888534 | <0.001 | NA |
| 20p13 | 1335621 | 1345731 | <0.001 | NA |
| 20p12.1 | 14843953 | 14852170 | 0.002 | C20orf133 |
| 20q13.31 | 55040991 | 55041191 | <0.001 | NA |
| 21q21.1 | 18615170 | 18936414 | <0.001 | PRSS7 |
| 21q21.2 | 24424204 | 24424999 | <0.001 | NA |
| 21q22.2 | 41332614 | 41332735 | <0.001 | NA |
| 22q12.1 | 26368374 | 26368467 | 0.013 | NA |
