# Supplementary material for: Genome-wide identification of significant aberrations in cancer genome
Source: BMC Genomics. 2012 Jul 27;13:342. doi: 10.1186/1471-2164-13-342 (PMC3428679; doi:10.1186/1471-2164-13-342)
Supplement: Additional file 5 — Table S8 and Table S9. Details about the implicated SCAs and full list of genes covered by these SCAs, derived from the glioblastoma data set. [file 1471-2164-13-342-S5.doc]

**Supplementary Table 8.** Details about the implicated SCAs and full list of genes covered by these SCAs, derived from genome-wide analysis of the glioblastoma data set. (Start: Start point of SCAs. End: End point of SCAs.) (Based on hg18 human genome assembly)

| **Cyto-**  **band** | **Region** | | **P-value** | **Genes covered by the SCA regions** |
| --- | --- | --- | --- | --- |
|  | **Start** | **End** |  |  |
| ***Amplification*** | | | | |
| 1q32.1 | 200943828 | 201665711 | <0.001 | SYT2,JARID1B,LOC163556,LOC647059,LOC148709,RABIF,KLHL12,ADIPOR1,LOC647243,CYB5R1,LOC401980,C1orf37,LOC647246,PPFIA4,MYOG,ADORA1,MYBPH,CHI3L1,CHIT1,BTG2,FMOD |
| 3q26.1 | 167359422 | 167359953 | 0.006 | NA |
| 4q12 | 52600433 | 56745024 | <0.001 | SPATA18,USP46,LOC643783,FLJ12684,LOC152578,RASL11B,SCFD2,FIP1L1,LNX1,LOC441016,LOC402176,CHIC2,LOC643901,LOC643915,GSH2,LOC442108,**PDGFRA**,KIT,KDR,SRD5A2L,TPARL,CLOCK,PDCL2,NMU,LOC644145,EXOC1,LOC644173,CEP135,LOC644187 |
| 4q12 | 56851338 | 57182948 | 0.004 | MRPL22P1,AASDH,PPAT,LOC644196,PAICS,SRP72,ARL9,GLDCP |
| 4q26 | 116626978 | 116627057 | 0.002 | NA |
| 6q23.2 | 132691401 | 132692996 | <0.001 | MOXD1 |
| 7p12.1 | 53364706 | 53756309 | <0.001 | FLJ45974 |
| 7p12.1 | 53789668 | 53879044 | 0.001 | FLJ45974 |
| 7p11.2 | 53891130 | 55360264 | <0.001 | LOC222005,MGC33530,LOC392030,SEC61G,LOC643168,**EGFR**,CALM1P2 |
| 7p11.2 | 55631083 | 55698217 | 0.002 | LOC643336,LOC442308,LOC643348 |
| 7q31.2 | 115873505 | 116895489 | <0.001 | CAV2,CAV1,**MET**,CAPZA2,ST7OT1,ST7,ST7OT4,ST7OT2,WNT2,ASZ1 |
| 9p12 | 41596599 | 41675369 | <0.001 | LOC642686 |
| 12p13.32 | 93683 | 5379126 | 0.025 | IQSEC3,SLC6A12,SLC6A13,JARID1A,MGC13183,B4GALNT3,NINJ2,WNK1,HSN2,RAD52,LOC642821,RAB6IP2,LOC653152,FBXL14,WNT5B,ADIPOR2,CACNA2D4,LOC644564,DCP1B,CACNA1C,LOC283440,LOC341511,FKBP4,MDS028,NRIP2,FOXM1,MGC13204,TULP3,TEAD4,LOC387825,TSPAN9,LOC643057,LOC643119,LOC643152,HRMT1L4,MGC4266,PARP11,HIN1L,LOC399988,LOC390281,**CCND2**,C12orf5,FGF23,FGF6,C12orf4,RAD51AP1,DYRK4,AKAP3,NDUFA9,GALNT8,KCNA6,KCNA1,LOC390282,KCNA5,LOC387826 |
| 12p13.31 | 6450843 | 6706803 | 0.003 | PKP2P1,MRPL51,CNAP1,GAPDH,HOM-TES-103,NOL1,CHD4,GPR92,ACRBP,ING4,ZNF384,DKFZp547D2210,COPS7A |
| 12p12.1 | 25134382 | 25537084 | 0.037 | LRMP,CASC1,LOC144363,**KRAS**,FLJ36004 |
| 12q13.2 | 56263307 | 56351715 | <0.001 | KIF5A,PIP5K2C,DTX3,GEFT,SLC26A10,B4GALNT1 |
| 12q13.3 | 56672392 | 56883837 | 0.001 | **CDK4** |
| 12q14.1 | 57216047 | 57257083 | 0.008 | NA |
| 12q14.3 | 67484379 | 67695739 | <0.001 | **MDM2**,CPM |
| 12q15 | 67957425 | 67957878 | 0.002 | NA |
| 15q11.1 | 19204681 | 19985304 | 0.015 | LOC646219,LOC646243,POTE15,LOC646257,LOC440225,OR11J2P,OR11J5P,LOC646271,LOC283804,LOC440226,LOC400968,LOC642548,OR11J1P,OR11H3P,OR11K1P,OR4Q1P,OR4H6P,OR4M2,OR4N4,OR4N3P,LOC388076,IGHV1OR15-1,VSIG6,LOC646370,LOC646372,LOC646379 |
| ***Deletion*** | | | | |
| 1p31.1 | 79787684 | 79787955 | <0.001 | NA |
| 2p21 | 44600493 | 44601507 | <0.001 | C2orf34 |
| 3p26.3 | 1510440 | 1511278 | <0.001 | NA |
| 3p26.2 | 3547161 | 3547242 | 0.006 | NA |
| 3p13 | 74171712 | 74172859 | 0.013 | NA |
| 4p15.32 | 15743369 | 15743714 | <0.001 | NA |
| 4q13.1 | 63875220 | 63875266 | 0.026 | NA |
| 4q28.1 | 126166023 | 126166261 | <0.001 | NA |
| 4q28.1 | 126837833 | 126839188 | 0.001 | NA |
| 4q34.3 | 177930796 | 177930985 | <0.001 | **VEGFC** |
| 5q15 | 96185799 | 96186078 | 0.001 | LOC642716 |
| 5q21.1 | 102672053 | 102672108 | <0.001 | NA |
| 5q22.2 | 111759059 | 111759533 | <0.001 | EPB41L4A |
| 6q16.1 | 93272279 | 93272358 | 0.013 | NA |
| 8p23.1 | 9224652 | 9225005 | <0.001 | LOC645986 |
| 9p24.3 | 1141090 | 1141269 | 0.006 | NA |
| 9p23 | 9589213 | 9589647 | 0.024 | NA |
| 9p23 | 9896906 | 9897092 | 0.026 | NA |
| 9p23 | 10538235 | 10885645 | 0.049 | LOC646087 |
| 9p23 | 11080005 | 11081095 | <0.001 | NA |
| 9p22.1 | 19726871 | 19817538 | 0.03 | SLC24A2 |
| 9p22.1 | 19830148 | 19830148 | 0.002 | NA |
| 9p22.1 | 19866496 | 19991563 | <0.001 | LOC646505 |
| 9p21.3 | 20105657 | 20241120 | 0.001 | NA |
| 9p21.3 | 20271390 | 27248185 | <0.001 | SMNP,MLLT3,KIAA1797,PTPLAD2,LOC646525,IFNB1,IFNW1,IFNA21,LOC392289,IFNA4,IFNA7,IFNA10,G13P1,LOC392291,IFNA16,IFNA17,LOC392292,IFNA14,IFNAP22,IFNA5,KLHL9,IFNA6,IFNA13,IFNA2,IFNWP12,IFNA8,LOC646581,IFNA1,IFNWP19,IFNE1,LOC402359,MTAP,C9orf53,**CDKN2A,CDKN2B**,LOC646605,DMRTA1,FLJ35282,LOC646609,LOC646611,LOC402360,ELAVL2,LOC646636,LOC646646,TUSC1,FLJ16323,C9orf82,PLAA,IFT74,LRRC19,TEK,C9orf14 |
| 9p21.2 | 27262672 | 28064589 | <0.001 | C9orf14,C9orf11,MOBKL2B,IFNK,C9orf72,LOC392298,LRRN6C |
| 9p21.1 | 28074987 | 28074987 | 0.022 | LRRN6C |
| 9p21.1 | 28089507 | 30373201 | <0.001 | LRRN6C,LOC646700,LOC653777,LOC286239,LOC646734 |
| 9p21.1 | 30425441 | 30926664 | 0.019 | LOC401497,LOC441391,LOC442405,LOC442406,LOC646753 |
| 9p21.1 | 31605043 | 31668988 | 0.049 | NA |
| 9p12 | 41596599 | 41675369 | 0.002 | LOC642686 |
| 10q21.1 | 55689588 | 55689938 | <0.001 | PCDH15 |
| 10q21.1 | 57863069 | 57863159 | <0.001 | NA |
| 10q23.2 | 88927564 | 88974858 | 0.001 | FAM35A,LOC653230 |
| 10q23.31 | 89402131 | 90332561 | <0.001 | PAPSS2,ATAD1,CFLP1,**PTEN**,C10orf59 |
| 10q23.31 | 90361944 | 90491738 | 0.011 | LOC389992,LIPF,LOC643414 |
| 10q23.31 | 90608793 | 90610385 | <0.001 | ANKRD22 |
| 12p12.2 | 20905406 | 20905430 | <0.001 | SLCO1B3 |
| 12q21.31 | 80609736 | 80610058 | 0.009 | PPFIA2 |
| 13q14.2 | 46808486 | 46973019 | <0.001 | NA |
| 13q21.33 | 71822299 | 71822493 | 0.001 | NA |
| 13q31.2 | 89128921 | 89128921 | 0.001 | NA |
| 13q33.2 | 105672579 | 105672794 | <0.001 | NA |
| 14q13.2 | 36125323 | 36172320 | 0.001 | NA |
| 17q21.31 | 44823714 | 44840245 | <0.001 | **PHB** |
| Xp22.33 | 202103 | 2330871 | <0.001 | PPP2R3B,SHOX,LOC645013,LOC653560,CSF2RA,IL3RA,SLC25A6,CXYorf2,ASMTL,P2RY8,DXYS155E,ASMT,LOC645080,DHRSX |

**Supplementary Table 9.** Details about the implicated SCAs and full list of genes covered by these SCAs, derived from individual chromosome analysis of the glioblastoma data set. (Start: Start point of SCAs. End: End point of SCAs.) (Based on hg18 human genome assembly)

| **Cyto-**  **band** | **Region** | | **P-value** | **Genes covered by the SCA regions** |
| --- | --- | --- | --- | --- |
|  | **Start** | **End** |  |  |
| ***Amplification*** | | | | |
| 1p12 | 119563124 | 119563514 | 0.011 | LOC343495 |
| 1q31.1 | 190739868 | 190740099 | 0.035 | NA |
| 1q32.1 | 200925185 | 201665711 | <0.001 | SYT2,JARID1B,LOC163556,LOC647059,LOC148709,RABIF,KLHL12,ADIPOR1,LOC647243,CYB5R1,LOC401980,C1orf37,LOC647246,PPFIA4,MYOG,ADORA1,MYBPH,CHI3L1,CHIT1,BTG2,FMOD |
| 2p24.3 | 15895946 | 16669661 | <0.001 | LOC130678,**MYCN**,LOC391353,FAM49A |
| 2p24.2 | 18301967 | 18302709 | 0.023 | NA |
| 3p24.3 | 20472299 | 20472299 | 0.003 | NA |
| 3p24.1 | 29306824 | 29307495 | 0.005 | RBMS3 |
| 3p21.31 | 45178235 | 45178235 | <0.001 | NA |
| 3q26.1 | 167359422 | 167359953 | <0.001 | NA |
| 4q11 | 52600433 | 59197382 | <0.001 | SPATA18,USP46,LOC643783,FLJ12684,LOC152578,RASL11B,SCFD2,FIP1L1,LNX1,LOC441016,LOC402176,CHIC2,LOC643901,LOC643915,GSH2,LOC442108,PDGFRA,KIT,KDR,SRD5A2L,TPARL,CLOCK,PDCL2,NMU,LOC644145,EXOC1,LOC644173,CEP135,LOC644187,MRPL22P1,AASDH,PPAT,LOC644196,PAICS,SRP72,ARL9,GLDCP,HOP,LOC391655,LOC285453,SPINK2,REST,C4orf14,POLR2B,IGFBP7,LOC255130,SRIL |
| 4q26 | 116626978 | 116627057 | <0.001 | NA |
| 4q31.23 | 150420181 | 150420273 | 0.001 | NA |
| 5q15 | 96185799 | 96186078 | <0.001 | LOC642716 |
| 5q21.3 | 109399376 | 109400056 | <0.001 | NA |
| 6p21.1 | 42688559 | 42898352 | <0.001 | UBR2,RDS,LOC442211,TBCC,FLJ38717,KIAA0240 |
| 6q12 | 63742722 | 63745031 | <0.001 | NA |
| 6q23.2 | 132691401 | 132692996 | <0.001 | MOXD1 |
| 7p12.1 | 53364706 | 55698217 | 0.007 | FLJ45974,LOC222005,MGC33530,LOC392030,SEC61G,LOC643168,**EGFR**,CALM1P2,LANCL2,ECOP,LOC643336,LOC442308,LOC643348 |
| 7q31.2 | 115873505 | 116895489 | <0.001 | CAV2,CAV1,**MET**,CAPZA2,ST7OT1,ST7,ST7OT4,ST7OT2,WNT2,ASZ1 |
| 8p23.1 | 6250239 | 6250491 | 0.001 | NA |
| 8p23.1 | 8976535 | 8977133 | 0.008 | NA |
| 8q24.23 | 137681615 | 137813119 | 0.001 | NA |
| 9p12 | 41219831 | 41675369 | <0.001 | LOC653114,LOC642629,LOC642639,LOC653120,LOC653123,ZNF658B,LOC642686 |
| 9q22.33 | 99830334 | 99830462 | <0.001 | NA |
| 10q21.1 | 57176910 | 57177032 | <0.001 | NA |
| 11q23.1 | 111062718 | 111426718 | <0.001 | SNF1LK2,PPP2R1B,ALG9,C11orf1,LOC644338,CRYAB,HSPB2,MGC14839,DIXDC1,DLAT,PPIHP1 |
| 12q13.2 | 56263307 | 56883837 | 0.016 | KIF5A,PIP5K2C,DTX3,GEFT,SLC26A10,B4GALNT1,LOC441641,OS9,CENTG1,TSPAN31,**CDK4**,MARCH9,CYP27B1,METTL1,DKFZP586D0919,TSFM,AVIL,CTDSP2,XRCC6BP1,LOC338805 |
| 12q14.3 | 67484379 | 67695739 | 0.001 | **MDM2**,CPM |
| 13q31.2 | 89066906 | 89066906 | <0.001 | NA |
| 14q11.1 | 18205576 | 18360294 | <0.001 | LOC441666 |
| 14q21.1 | 42241131 | 42309661 | 0.035 | NA |
| 15q11.1 | 19204681 | 20686180 | 0.016 | LOC646219,LOC646243,POTE15,LOC646257,LOC440225,OR11J2P,OR11J5P,LOC646271,LOC283804,LOC440226,LOC400968,LOC642548,OR11J1P,OR11H3P,OR11K1P,OR4Q1P,OR4H6P,OR4M2,OR4N4,OR4N3P,LOC388076,IGHV1OR15-1,VSIG6,LOC646370,LOC646372,LOC646379,LOC646396,LOC646401,ABCB10P,GOLGA8D,LOC653736,LOC646411,LOC283767,LOC390544,TUBGCP5,CYFIP1,NIPA2,NIPA1,LOC283683,LOC400320 |
| 17q12 | 34584689 | 34584909 | 0.027 | CACNB1 |
| 19q13.41 | 52544997 | 52546246 | <0.001 | DHX34 |
| 21q21.1 | 20518399 | 20519081 | <0.001 | NA |
| 21q22.11 | 35497145 | 35569980 | <0.001 | NA |
| 22q11.1 | 14914113 | 16763636 | 0.003 | LOC644734,ABCD1P4,LOC644758,LOC644768,LOC644773,LOC644784,KCNMB3L,CESK1,FABP5P1,psiTPTE22,LOC644802,ADPRTL4,VWFP,XKR3,ZNF402P,LOC644845,LOC644851,LOC440786,LOC644857,GAB4,VN1R9P,CECR7,IL17R,CECR6,CECR5,CECR1,RPL32L2,LOC644899,CLCP1,CECR2,SLC25A18,ATP6V1E1,BCL2L13,BID,MICAL3,LOC642566 |
| ***Deletion*** | | | | |
| 1p32.2 | 57053184 | 57053943 | 0.007 | C1orf168 |
| 1p31.1 | 79787684 | 79787955 | <0.001 | NA |
| 1q21.3 | 150658038 | 150658038 | 0.03 | NA |
| 2p21 | 44600493 | 44601507 | <0.001 | C2orf34 |
| 2q33.3 | 205318165 | 205319367 | 0.001 | ALS2CR19 |
| 3p26.3 | 1510440 | 1511278 | <0.001 | NA |
| 3p26.2 | 3547161 | 3547242 | <0.001 | NA |
| 3p13 | 74171712 | 74172859 | <0.001 | NA |
| 4p15.33 | 13125113 | 13125345 | 0.03 | NA |
| 4p15.32 | 15743369 | 15743714 | <0.001 | NA |
| 4q13.1 | 63316180 | 63316346 | 0.015 | NA |
| 4q13.1 | 63875220 | 63875266 | <0.001 | NA |
| 4q21.22 | 83302365 | 83309366 | 0.03 | NA |
| 4q28.1 | 126166023 | 126166261 | <0.001 | NA |
| 4q28.1 | 126837833 | 126839188 | <0.001 | NA |
| 4q28.3 | 131998808 | 131999387 | 0.013 | NA |
| 4q34.3 | 177930796 | 177930985 | <0.001 | VEGFC |
| 5q14.3 | 85715451 | 85715539 | 0.028 | NA |
| 5q15 | 96185799 | 96186078 | <0.001 | LOC642716 |
| 5q15 | 97146613 | 97155593 | 0.016 | NA |
| 5q21.1 | 102672053 | 102672108 | <0.001 | NA |
| 5q22.2 | 111759059 | 111759533 | <0.001 | EPB41L4A |
| 6q16.1 | 93272279 | 93272358 | <0.001 | NA |
| 7p21.1 | 17445757 | 17446243 | 0.034 | NA |
| 7q22.3 | 104694484 | 104843430 | <0.001 | SRPK2 |
| 7q35 | 144688085 | 144711054 | 0.02 | NA |
| 8p23.2 | 4125908 | 4132275 | 0.002 | **CSMD1** |
| 8p23.1 | 9224652 | 9225005 | <0.001 | LOC645986 |
| 9p23 | 11080005 | 11081095 | <0.001 | NA |
| 9p22.1 | 19830148 | 30373201 | <0.001 | LOC646505,SMNP,MLLT3,KIAA1797,PTPLAD2,LOC646525,IFNB1,IFNW1,IFNA21,LOC392289,IFNA4,IFNA7,IFNA10,G13P1,LOC392291,IFNA16,IFNA17,LOC392292,IFNA14,IFNAP22,IFNA5,KLHL9,IFNA6,IFNA13,IFNA2,IFNWP12,IFNA8,LOC646581,IFNA1,IFNWP19,IFNE1,LOC402359,MTAP,C9orf53,**CDKN2A,CDKN2B**,LOC646605,DMRTA1,FLJ35282,LOC646609,LOC646611,LOC402360,ELAVL2,LOC646636,LOC646646,TUSC1,FLJ16323,C9orf82,PLAA,IFT74,LRRC19,TEK,C9orf14,C9orf11,MOBKL2B,IFNK,C9orf72,LOC392298,LRRN6C,LOC646700,LOC653777,LOC286239,LOC646734 |
| 9p12 | 41596599 | 41675369 | 0.043 | LOC642686 |
| 10q21.1 | 55689588 | 55689938 | <0.001 | PCDH15 |
| 10q21.1 | 57863069 | 57863159 | <0.001 | NA |
| 10q23.2 | 88927564 | 90610385 | <0.001 | FAM35A,LOC653230,LOC643202,LOC439994,FAM22A,LOC118945,MINPP1,PAPSS2,ATAD1,CFLP1,PTEN,C10orf59,LIPL1,LOC389992,LIPF,LOC643414,LOC643418,LIPL3,ANKRD22 |
| 11p12 | 37612068 | 37612801 | 0.017 | NA |
| 11q22.2 | 102232825 | 102248188 | 0.003 | MMP12 |
| 12p13.31 | 7757402 | 7779822 | <0.001 | DPPA3,CLEC4C |
| 12p12.2 | 20905406 | 20905430 | <0.001 | SLCO1B3 |
| 12p12.1 | 25624364 | 25625036 | 0.002 | NA |
| 12q12 | 41993388 | 41998556 | <0.001 | NA |
| 12q21.31 | 80609736 | 80610058 | <0.001 | PPFIA2 |
| 12q21.31 | 81160680 | 81161074 | <0.001 | NA |
| 13q12.11 | 21863424 | 21863812 | 0.028 | NA |
| 13q14.13 | 46808486 | 46973019 | <0.001 | NA |
| 13q21.2 | 62248424 | 62248579 | 0.02 | NA |
| 13q21.33 | 71822299 | 71822493 | <0.001 | NA |
| 13q22.2 | 76565412 | 76566942 | 0.007 | MYCBP2 |
| 13q31.2 | 89128921 | 89128921 | <0.001 | NA |
| 13q33.2 | 105672579 | 105672794 | <0.001 | NA |
| 14q13.2 | 36125323 | 36172320 | <0.001 | NA |
| 15q21.2 | 51989931 | 51990382 | <0.001 | NA |
| 16p13.2 | 8431941 | 8434944 | 0.014 | NA |
| 16p13.12 | 13717133 | 13717163 | 0.021 | NA |
| 17q21.31 | 44823714 | 44840245 | <0.001 | PHB |
| 17q22 | 51308788 | 51309721 | 0.045 | NA |
| 19q13.33 | 48043004 | 48163405 | <0.001 | LOC653492,PSG1,PSG6,PSG7,CEACAMP7 |
| 19q13.33 | 48326010 | 48326010 | <0.001 | PSG11 |
| 21q22.11 | 34195324 | 34195637 | <0.001 | NA |
| 22q11.23 | 24031997 | 24244046 | 0.019 | LOC91353,LOC646137,LOC646141,LRP5L,LOC646180,CRYBB2P1 |
